# Effect of Antigen Valency on Autoreactive B-Cell Targeting

**DOI:** 10.1021/acs.molpharmaceut.3c00527

**Published:** 2023-10-20

**Authors:** M. J. van Weijsten, K. R. Venrooij, L.P.W.M. Lelieveldt, T. Kissel, E. van Buijtenen, F. J. van Dalen, M. Verdoes, R.E.M. Toes, K. M. Bonger

**Affiliations:** † Institute for Molecules and Materials, Radboud University, Heyendaalseweg 135, 6525 AJ Nijmegen, The Netherlands; ‡ Department of Rheumatology, Leiden University Medical Center, Albinusdreef 2, 2333 ZA Leiden, The Netherlands; § Department of Medical BioSciences, Radboudumc, Geert Grooteplein Zuid 28, 6525 GA Nijmegen, The Netherlands; ∥ Institute for Chemical Immunology, 6525 GA Nijmegen, The Netherlands

**Keywords:** B-cell receptor targeting, autoimmune disease, multivalency, immunotherapy, cell response

## Abstract

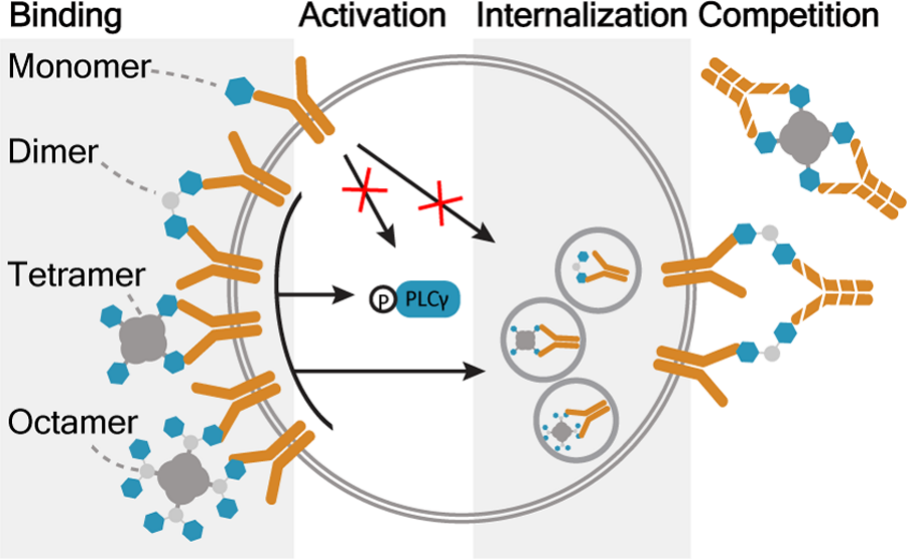

Many autoimmune diseases are characterized by B cells that mistakenly recognize autoantigens and produce antibodies toward self-proteins. Current therapies aim to suppress the immune system, which is associated with adverse effects. An attractive and more specific approach is to target the autoreactive B cells selectively through their unique B-cell receptor (BCR) using an autoantigen coupled to an effector molecule able to modulate the B-cell activity. The cellular response upon antigen binding, such as receptor internalization, impacts the choice of effector molecule. In this study, we systematically investigated how a panel of well-defined mono-, di-, tetra-, and octavalent peptide antigens affects the binding, activation, and internalization of the BCR. To test our constructs, we used a B-cell line expressing a BCR against citrullinated antigens, the main autoimmune epitope in rheumatoid arthritis. We found that the dimeric antigen construct has superior targeting properties compared to those of its monomeric and multimeric counterparts, indicating that it can serve as a basis for future antigen-specific targeting studies for the treatment of RA.

## Introduction

Rheumatoid arthritis (RA) is an autoimmune disease affecting approximately 1% of the Western population and involves joint inflammation, stiffness, and bone erosion.^1^ Though the exact cause of RA is yet unclear, autoreactive B cells are known to play a key role in its disease pathogenesis.^2^

Specific autoreactive B cells can make up to 0.2% of the total B-cell population and are characterized by a B-cell receptor (BCR) that recognizes a specific self-antigen.^3^ The majority of the patients with established RA recognize and produce anticitrullinated protein antibodies (ACPAs) that can be detected years before the onset of the disease and their presence is associated with disease onset and severity.^2,4^

RA is currently treated with disease-modifying antirheumatic drugs (DMARDs), including biologic DMARDs against the general B-cell marker CD20. Treatment with anti-CD20 eliminates the naïve and memory B-cell population, which often effectively reduces inflammation but also leaves the patient vulnerable to adverse effects, including infections.^5,6^

Antigen-specific immunotherapy aims to abrogate potential side effects from immunosuppressive therapy and has been a topic of interest for several decades.^7−10^ In this approach, an autoantigen is coupled to an effector molecule that can modulate the immune- or cellular response, such as a complement activating peptide, antibody Fragment crystallizable (Fc) tail, engagers for inhibitory receptors, or a potent toxin.^11−14^ Several of the reported strategies reduced or eliminated the autoreactive B-cell compartment selectively without affecting the protective B cells.

In our studies, we previously explored a sequential prodrug approach for antigen-specific B-cell elimination.^15^ Here, we modified a cyclic citrullinated peptide (CCP) antigen with biotin and added this construct to a tetrameric streptavidin protein that was conjugated to a toxin. Using this tetravalent antigen–effector construct, we demonstrated antigen-specific cell death on an immortalized B-cell clone derived from patients with RA.^16^ However, as streptavidin is based on a bacterial-derived protein, its use as a scaffold for cell targeting is not desired as it may induce unwanted immune adverse effects.^17,18^

In order to obtain low-valent targeting constructs that are more accessible and can be used in a therapeutic setting, we opted to develop a simpler and nonprotein-derived scaffold molecule. While it is well-established that antigen valency has a large impact on BCR targeting,^19,20^ the exact requirements of the BCR for internalization or activation remain disputed and seem to depend on the type of antigen, antigen structure, and spacing.^21−23^ Moreover, only a few studies have systematically investigated the effect of synthetic low-valent antigens on BCR avidity, signaling, and internalization of the antigen.^24,25^

In this study, we investigated the effect of well-defined low-valent antigen molecules in a model cell line of RA expressing a BCR derived from a patient with RA directed against citrullinated antigens. We synthesized monomers and dimers of citrullinated peptide antigen CCP4 and evaluated the effect of the constructs on binding avidity, BCR-induced signaling, and antigen internalization and localization. In addition, we modified the monomers and dimers with a biotin moiety to create streptavidin-based tetramers and octamers, respectively, which we evaluated for comparison. As free-circulating ACPAs are present in high concentrations in patients with RA, we further evaluated how the presence of ACPA affects the binding of the constructs to the B cells. We show that our dimeric and higher oligomeric constructs bind to the BCR with high avidity and several orders of magnitude higher avidity compared to the monomeric antigen. In addition, the dimer and oligomers induced BCR clustering, as evidenced by the induction of BCR signaling and receptor internalization. Further, we demonstrated that the dimer was less affected by circulating antibodies compared to higher, streptavidin-bound oligomeric constructs. Our results indicate that a dimeric antigen with short spacing between the targeting antigens serves as a promising targeting construct for antigen-specific delivery of effector molecules into autoreactive B cells.

## Experimental Section

For detailed synthesis procedures, see the Supporting Information.

### N2-(2-Aminoethyl)-N4,N6-di(prop-2-yn-1-yl)-1,3,5-triazine-2,4,6-triamine (**3**)

Triazin **2** (98.0 mg, 0.30 mmol, 1.0 equiv) was dissolved in anhydrous DMSO (2 mL) at RT. Subsequently, DIPEA (0.1 mL, 0.6 mmol, 2 equiv) and propargyl amine (0.29 mL, 4.5 mmol, 15 equiv) were added. The mixture was heated at 80 °C for 4 h and subsequently concentrated in vacuo. The reaction mixture was diluted with 6 mL of DCM. Then, TFA (6 mL) was slowly added. The reaction was stirred at room temperature for 2 h and 15 min. Next, the reaction mixture was coevaporated with chloroform and concentrated in vacuo. The product was purified using automated flash column chromatography (0–100% ACN in MQ + 0.1% TFA, 30 mL/min), product fractions were lyophilized, and N2-(2-aminoethyl)-N4,N6-di(prop-2-yn-1-yl)-1,3,5-triazine-2,4,6-triamine (60.3 mg, 0.176 mmol, 58.6%) was obtained as a light-yellow solid. Rf-value: 0.24 (1% Et_3_N in 10% MeOH in DCM) ^1^H NMR (400 MHz, D_2_O) δ 4.35–4.12 (m, 4H), 3.92–3.66 (m, 2H), 3.34–3.20 (m, 2H), 2.76–2.60 (m, 2H). ^13^C NMR (101 MHz, D_2_O) δ: 163.05, 162.69, 72.52, 39.13, 38.30, 29.94. LRMS (ESI+) *m*/*z* calculated for C_10_H_16_N_7_ [M + H]^+^ 246.29, found 246.30.

### N2-(2-Aminoethyl)-N4,N6-di(prop-2-yn-1-yl)-1,3,5-triazine-2,4,6-triamine (**4**)

The triazin **3** (37.23 mg, 104.2 μmol, 1 equiv) was dissolved in anhydrous DMF in a flame-dried round-bottom flask under argon, filled with activated mol sieves (3A). DIPEA (89.5 μL, 67.34 mg, 0.521 mmol, 5 equiv) was then added and the solution was left overnight under argon, stirring at 100 rpm. The following day, Biotin-NHS (43.0 mg, 0.126 μmol, 1.21 equiv) was added to the reaction vessel. After 2 h the reaction mixture filtered and diluted with 3 mL of MQ. After stirring for 30 min at 700 rpm, the reaction mixture was concentrated in vacuo to yield an off-white solid (33.2 mg). This product was used as crude in the subsequent reaction. LCMS (ESI+) *m*/*z* calcd for C_21_H_30_N_9_O_2_S [M + H]^+^ 472.223776, found 472.48.

### Cyclic Citrullinated Peptide 4 (CCP4, **5**)

This peptide was synthesized with Fmoc-based SPPS and cyclized, following the procedure of Lelieveldt et al.^15^ HPLC: Rt. 15.133 min. HRMS (ESI+) *m*/*z* calcd for C_81_H_128_N_27_O_20_S [M + H]^+^ 1830.95496, found 1830.95469.

### General Procedure for Modification of CCP4 by NHS Chemistry

In a flame-dried flask under argon, anhydrous DMSO of DMF, CCP4 **5**, and 1 equiv of an NHS-bearing molecule were added. Subsequently, 10 equiv of anhydrous DIPEA was added. The mixture was left to stir for 30–90 min in the dark and then diluted with MQ:ACN:TFA (8:2:0.1), centrifuged (4500*g*, 5 min, RT), and the supernatant was purified with RP HPLC. The product fractions were concentrated in vacuo and lyophilized.

### General Procedure CuAAC to Synthesize Dimeric CCP4

MQ and triazin **3** or **4** were placed in a flask. Subsequently, THPTA (10 equiv) was added and the solution was bubbled through with argon for 30 min. CuSO_4_·5H_2_O (2 equiv) was then added and then CCP4-N_3_ (**6**, 2 equiv). Finally, sodium ascorbate (10 equiv) was added, and the reaction was monitored using liquid chromatography–mass spectrometry (LCMS) or high-performance liquid chromatography (HPLC). Upon completion, the mixture was diluted with MQ/ACN/TFA (8:2:0.1), centrifuged (4500*g*, 5 min, RT), and the supernatant was purified with RP HPLC. The product fractions were concentrated in vacuo and lyophilized.

### CCP4-N_3_ (**6**)

HPLC: Rt. 16.408 min. LCMS (ESI+) *m*/*z* calcd for C_83_H_129_N_30_O_21_S [M + H]^+^ 1913.97, found 1914.88. C_83_H_130_N_30_O_21_S [M + 2H]^2+^ 957.49, found 957.68. C_83_H_131_N_30_O_21_S [M + 3H]^3+^ 638.66, found 639.04. HRMS (ESI+) *m*/*z* calcd for C_83_H_129_N_30_O_21_S [M + H]^+^ 1913.96692, found 1913.97036.

### CCP4-Biotin (**7**)

HPLC: Rt. 16.256 min. LCMS (ESI+) *m*/*z* calcd for C_91_H_143_N_29_O_22_S_2_ [M + 2H]^2+^ 1029.02, found 1029.84. C_91_H_144_N_29_O_22_S_2_ [M + 3H]^3+^ 686.35, found 686.92. HRMS (ESI+) *m*/*z* calcd for C_91_H_142_N_29_O_22_S_2_ [M + H]^+^ 2057.03256, found 2057.02729.

### CCP4-AF594 (**8**)

HPLC: Rt. 18.123 min. HRMS (ESI+) *m*/*z* calcd for C_116_H_159_N_29_Na_2_O_30_S_3_ [M + 2Na]^2+^ 2580.07651, found 2580.07838. LCMS (ESI+) *m*/*z* calcd for C_116_H_161_N_29_O_30_S_3_ [M + 2H]^2+^ 1268.06, found 1268.76. C_116_H_162_N_29_O_30_S_3_ [M + 3H]^3+^ 845.71, found 846.24.

### CCP4-SCy5 (**9**)

HPLC: Rt. 17.509 min. HRMS (ESI+) *m*/*z* calcd for C_113_H_164_N_29_O_27_S_3_ [M + 2H]^2+^ 1228.07959, found 1228.07731. C_113_H_165_N_29_O_27_S_3_ [M + 3H]^3+^ 819.38995, found 819.38689.

### CCP4(Dimer)-NH_2_ (**10**)

HPLC: Rt. 15.904 min. HRMS (ESI+) *m*/*z* calcd for C_177_H_273_N_67_O_42_S_2_ [M + 2H]^2+^ 2037.53886, found 2037.53517. C_177_H_274_N_67_O_42_S_2_ [M + 3H]^3+^ 1358.69518, found 1358.69121. C_177_H_275_N_67_O_42_S_2_ [M + 4H]^4+^ 1019.27334, found 1019.26812.

### CCP4(Dimer)-Biotin (**11**)

HPLC: Rt. 16.415 min. LCMS (ESI+) *m*/*z* calcd for C_187_H_288_N_69_O_44_S_3_ [M+3H]^3+^ 1433.39, found 1434.48. C_187_H_289_N_69_O_44_S_3_ [M + 4H]^4+^ 1075.29, found 1076.12. C_187_H_290_N_69_O_44_S_3_ [M + 5H]^5+^ 860.43, found 861.12. C_187_H_291_N_69_O_44_S_3_ [M + 6H]^6+^ 717.20, found 717.68. C_187_H_292_N_69_O_44_S_3_ [M + 7H]^7+^ 614.88, found 615.44. C_187_H_293_N_69_O_44_S_3_ [M + 8H]^8+^ 538.15, found 538.56.

### CCP4(Dimer)-AF594 (**12**)

HPLC: Rt. 17.450 min. HRMS (ESI+) calcd for C_212_H_305_N_69_O_52_S_4_ [M + 2H]^2+^ 2390.11474, found 2390.07373. C_212_H_306_N_69_O_52_S_4_ [M + 3H]^3+^ 1593.74577, found 1593.74317. C_212_H_307_N_69_O_52_S_4_ [M + 4H]^4+^ 1195.56128, found 1195.55163. C_212_H_308_N_69_O_52_S_4_ [M + 5H]^5+^ 956.65059, found 956.64632.

### CCP4(Dimer)-SCy5 (**13**)

HPLC: Rt. 17.069 min. LCMS (ESI+) *m*/*z* calcd for C_209_H_310_N_69_O_49_S_4_ [M + 3H]^3+^ 1566.09, found 1566.76. C_209_H_311_N_69_O_49_S_4_ [M + 4H]^4+^ 1174.82, found 1175.60. C_209_H_312_N_69_O_49_S_4_ [M + 5H]^5+^ 940.06, found 940.76. C_209_H_313_N_69_O_49_S_4_ [M + 6H]^6+^ 783.55, found 784.08

### Synthesis CCP4 Tetramer and Octamer

Monomer-biotin or dimer-biotin was added to streptavidin (Streptavidin, Bioconnect, 016-000-113 or Streptavidin-AF594, FisherScientific, 10626153 or Streptavidin AF647, Bioconnect, 016-600-084) in a 10–40× excess to a final concentration of approximately 1 mg/mL, to create the tetramers and octamers, respectively. 10% glycerol was added to the incubation with dimer-biotin to prevent precipitation.

The mixture was incubated o/n at 4 °C with agitation. The remaining unbound peptide was removed through multiple rounds of dialysis with PBS.

### General Cell Culture

The generation of Ramos 3F3, TT, and MLD-AID KO cells was described previously.^16,26^ For an overview of the BCR sequence of the Ramos 3F3 and TT cells, see Table S1. Cells were cultured in RPMI 1640 with Hepes and GlutaMax (FisherScientific, 11544526), supplemented with 10% FCS OneShot (FisherScientific 15595309), and 1% penicillin/streptomycin. Cells were maintained between 0.25 and 2.5 × 10^6^ cells/mL.

### ELISA

A 96-well plate was coated with streptavidin, o/n, at 37° (1 μg/mL in carbonate-bicarb buffer, pH 9.6). Ten μg/mL of peptide in carbonate buffer was added to the plate and incubated for 1 h at RT. Washing steps were performed with PBS containing 0.05% Tween 20. The primary antibody (3F3 antibodies from Theresa Kissel) and secondary antibody (goat anti-Human HRP, 1:5000, Abcam, ab97225) were diluted in PBS containing 1% BSA and 0.05% Tween 20, and sequentially incubated for 1 h at 37 °C. Results were analyzed with a Tecan Spark M10 plate reader.

### Flow Cytometry

#### Binding Assays

For the tetramer and octamer binding curves, cells were incubated with tetramer-AF647 or octamer-647 (15 min, 4 °C). For the monomer and dimer binding curves, monomer-biotin or dimer-biotin was used, and a second incubation with streptavidin-647 was performed (15 min, 4 °C). The cells were fixed with 4% PFA (15 min, RT) and analyzed with the BD FACSverse. For the general gating strategy, see Figure S7.

#### p-PLCγ Assay

Cells were incubated with the indicated compounds (10 min, 4 °C and 15 min, 37 °C). Cells were fixed with 4% PFA (15 min, RT) and permeabilized with 0.3% saponin in PBS (30 min, RT). Anti-p-PLCγ (BD Biosciences, 558498) was added 1:100 (1 h, RT). Cells were washed and analyzed with the BD FACSverse. For the general gating strategy, see Figure S7.

### Competition Assay

Ramos 3F3 antibodies and the indicated constructs were incubated at a 1:1 3F3-binding-site:CCP4 ratio, at a 4 μM final concentration (1 h, 37 °C). Cells were treated with FcR blocking reagent (Miltenyi Biotech, 130-059-901) for 30 min at 4 °C. The antibody-construct samples were diluted to 100 nM CCP4 concentration and added to the cells. The cells were incubated for 15 min at 4 °C, fixed with 4% PFA (15 min, RT), and analyzed with the BD FACSverse.

### General Microscopy

Cells were incubated with the indicated constructs for an indicated amount of time. Cells were fixed with 4% PFA (15 min, RT) and transferred to an Ibidi uncoated μ-slide. Analysis was performed with a Leica SP8 AOBS microscope at 100× magnification. All images represent slices in the center of a z-stack (minimum 5 slices).

### Immunostaining

Cells were incubated with the indicated constructs for 30 min. Next, the cells were adhered to PLL-coated coverslips (15 min, 37 °C) and fixed with 4% PFA (15 min, RT). The cells were treated with 10 mM Glycine and permeabilized with 0.3% saponin or 0.1% Triton-X100 in PBS (20 min, RT). After blocking with 5% NGS in PBS + 0.1% Tween 20, the cells were incubated with anti-EEA1 (Invitrogen, MA5-14794) or anti-LAMP1 (Fisher Scientific, 14-1079-80) 1:100 (1 h, RT). Goat anti-Rabbit 555 (Fisher Scientific, 10082602) or Goat anti-Mouse 568 (Invitrogen, A11004) was added at 1:200 (45 min, RT). Cells were mounted with Mowiol and analyzed with the Leica SP8 AOBS microscope at 100× magnification.

### Colocalization Analysis

Images were analyzed with Fiji/ImageJ. Mander’s colocalization coefficient was calculated using the JaCoP plugin for each individual cell.^27^ Cells containing no antigen or antibody staining or containing autofluorescence were excluded from analysis. Thresholding was performed using the Renyi thresholding method.^28^ A minimum of 100 cells was analyzed per sample. After quantitative analysis, ten randomly selected cells were analyzed manually to confirm the batch results.

### Statistical Analysis

The results were expressed as mean with standard deviation (SD). Statistical significance was determined using Student’s *t* tests (with a correction for multiple testing if required), where *p* < 0.05 was considered significant. All statistical analysis was performed with GraphPad Prism 5.0 or 9.0 (GraphPad Software Inc., La Jolla, CA).

## Results

### Design and Synthesis of Multivalent Targeting Constructs

**Figure 1:**
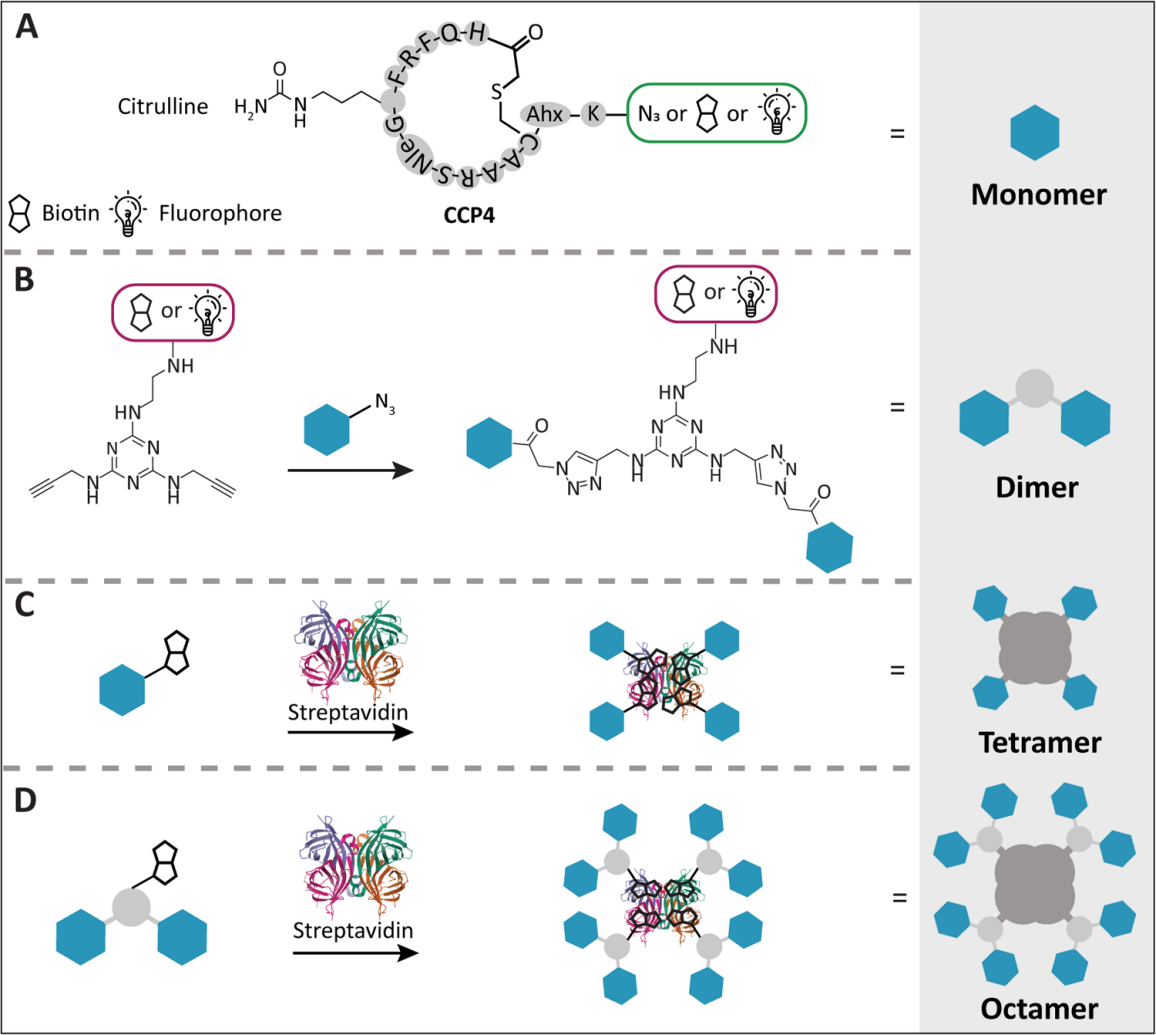
Construct design: (A) The CCP4 monomer contains a citrulline and was cyclized between the cysteine side chain and the chloroacetamide-modified N-terminus. The lysine was functionalized with a biotin, azide, or AF594 using NHS chemistry. (B) The CCP4 dimer was synthesized by reacting the alkynes of the triazin core with an azide-modified CCP4 monomer using CuAAC chemistry. The triazine amine was functionalized with either biotin or AF594 or Cy5 using NHS chemistry. (C) The tetramer was obtained by adding CCP4 monomer-biotin to streptavidin or streptavidin-AF594. (D) The octamer was obtained by adding the biotin-modified CCP4 dimer to streptavidin or streptavidin-AF594.

For our studies, we used a cyclic citrullinated peptide, CCP4, as our model antigen (Figure 1A).^16^ The CCP4 sequence is derived from CCP2, a diagnostic peptide marker used for the detection of ACPAs in patients with RA, and binds with comparable affinity as determined by ELISA (Figure S1A).^29^ The peptide was obtained by solid-phase peptide synthesis using standard synthetic protocols. We included a lysine and an aminohexanoic acid (Ahx) linker at the end of the sequence to allow selective modification of the peptide through the single free ε-amine group of lysine. After the last coupling, the N-terminus was modified with a chloroacetamide. After cleavage from the resin and deprotection of the residue side chains, the peptide was cyclized between the cysteine side chain and the modified N-terminus by dissolving the peptide in a basic buffer under diluted conditions. The peptide was further functionalized using NHS chemistry with either a biotin, an azide, or AF594- or AF647 or sulfo-Cy5 fluorophores, depending on the application.

For the dimeric peptides, we used a 1,3,5-triazin moiety that has previously been explored as a scaffold for multivalent targeting constructs.^30,31^ We envisioned that two arms of the triazin could be coupled to a CCP4 peptide, while the third group could be used for fluorophore or biotin conjugation. For this, we synthesized a triazin with two alkyne groups and one amine handle. The alkynes were conjugated to two azide-containing CCP4 peptides via a copper-catalyzed azide–alkyne cycloaddition (CuAAC) reaction. The amine was functionalized with either a biotin, AF594-, or sulfo-Cy5-fluorophore using NHS chemistry (Figure 1B). For comparison, we additionally prepared tetrameric and octameric multimers by incubating streptavidin (containing an AF549-, an AF647-, or no fluorophore) with the biotin-functionalized CCP4 monomer or dimer, respectively (Figure 1C,D).

To evaluate the BCR binding avidity, activation, and internalization of our constructs, we used an engineered B-cell line (Ramos) that stably expresses a patient-derived BCR sequence recognizing cyclic citrullinated peptides as previously described.^16^ Briefly, the genes encoding the endogenous IgM and IgD heavy and light chains and the activation-induced cytidine deaminase (AID) protein were first deleted, creating Ramos MDL-AID KO cells. Next, the MDL-AID KO cells were transduced with an IgG sequence derived from ACPA-positive B cells from a patient with RA, creating Ramos 3F3 cells.^16^ As control cell lines, we used the Ramos MDL-AID KO cells and Ramos cells transduced with an IgG sequence derived from B cells that recognize tetanus toxoid (TT), hereafter named Ramos TT cells.^32^

### Effect of Antigen Valency on Receptor Binding Avidity

**Figure 2:**
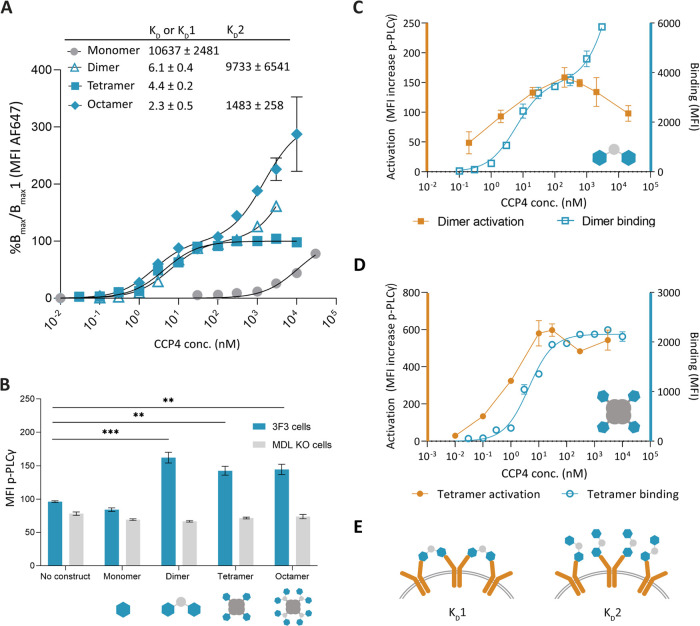
Binding of the CCP4 constructs with Ramos 3F3 cells. (A) Representative binding curves of all of the constructs normalized to either their *B*_max_ (monomer and tetramer) or their *B*_max_1 (dimer and octamer). The dimer, tetramer, and octamer show 3 orders of magnitude decrease in apparent *K*_D_ compared to the monomer. The constructs were incubated for 15 min at 4 °C. *N* = 3. (B) The dimer, tetramer, and octamer significantly increase p-PLCγ as compared to nonstimulated 3F3 cells and MDL KO cells. All constructs were incubated at 1 μM CCP4 concentration, 10 min at 4 °C and 15 min at 37 °C. *N* = 3. * *p* ≤ 0.05, ** *p* ≤ 0.01, *** *p* ≤ 0.001. (C, D) p-PLCγ increase (left axis) for the dimer (C) and tetramer (D) at different concentrations vs their respective binding curves (right axis). The constructs were incubated for 10 min at 4 °C, 15 min at 37 °C for the activation experiment, and 15 min at 4 °C for the binding experiment. *N* = 3. (E) Possible binding modes of the dimer. At low concentrations, the dimer binds with two antigens, resulting in the first saturation step and an apparent *K*_D_ value (*K*_D_1). At higher concentrations, the dimer binds with only one antigen, leading to the second saturation step and an apparent *K*_D_ value (*K*_D_2).

Multivalent binding curves are most accurately described in terms of avidity, which adds the effect of ligand valency and architecture affinity to the basic affinity of a monomeric antigen.^33,34^ We first analyzed the binding avidity of our constructs to Ramos 3F3 cells by flow cytometry (Figure 2A). We used CCP4 constructs modified with a fluorophore and titrated the constructs based on their effective CCP4 concentration to confirm that differences in binding avidity are a result of their multivalent nature. Due to the different fluorophore to CCP4 ratios between the constructs (Table S2), we normalized the data to the first saturation point of each construct (*B*_max_ for the monomer and tetramer, or *B*_max_1 for the dimer and octamer). Using these conditions, the monomer shows the weakest binding with a dissociation constant (*K*_D_) in the μM range. The dimer, tetramer, and octamer show a decreased apparent *K*_D_ of 3 orders of magnitude compared to the monomer. We observed two saturation points of the dimer and octamer, resulting in a *K*_D_1 and *K*_D_2 value (Figure S1B), indicating that the constructs bridge the BCRs at low concentrations while a saturated one-arm binding event occurs at higher concentrations (Figure 2E).^33^ We did not observe multiple saturating events when using the tetrameric construct. None of the constructs show significant binding on MDL KO cells, as expected (Figure S1C,D).

### Effect of Antigen Valency on B-Cell Activation

To investigate the effect of valency on B-cell activation, we measured the increase in the early activation marker phosphorylated PLCγ (p-PLCγ) by flow cytometry upon addition of the different construct concentrations.^35^ At a saturated binding concentration of 1 μM CCP4, we found that the dimer, tetramer, and octamer increased p-PLCγ levels compared to nontreated cells, indicating that these constructs are able to cross-link the BCR and induce signaling. The monomer does not raise p-PLCγ levels at the tested concentrations (Figures 2B and S2A–D). The MDL KO cells showed no increase in p-PLCγ when treated with 1 μM of all constructs (Figure S2A–D).

As we observed a clear two-stage saturation event using the dimeric constructs, we hypothesize that the binding of the dimer with only one of its ligands at high concentrations could also have physiological consequences, as BCR clustering might be affected. We evaluated B-cell activation by incubating Ramos 3F3 cells with either the dimer or the tetramer at various concentrations and measured the p-PLCγ levels. Indeed, the dimer induced a dose-dependent increase in p-PLCγ that decreased at higher CCP4 concentrations. The tetramer also showed a dose-dependent increase, plateauing at 100 nM (Figure 2C,D). We also titrated the dimer and tetramer on Ramos TT cells to confirm that activation was not due to aggregation effects. Though background activation increases slightly at higher concentrations, the overall background signal is low (Figure S2E,F).

### Effect of Antigen Valency on Receptor Internalization

**Figure 3:**
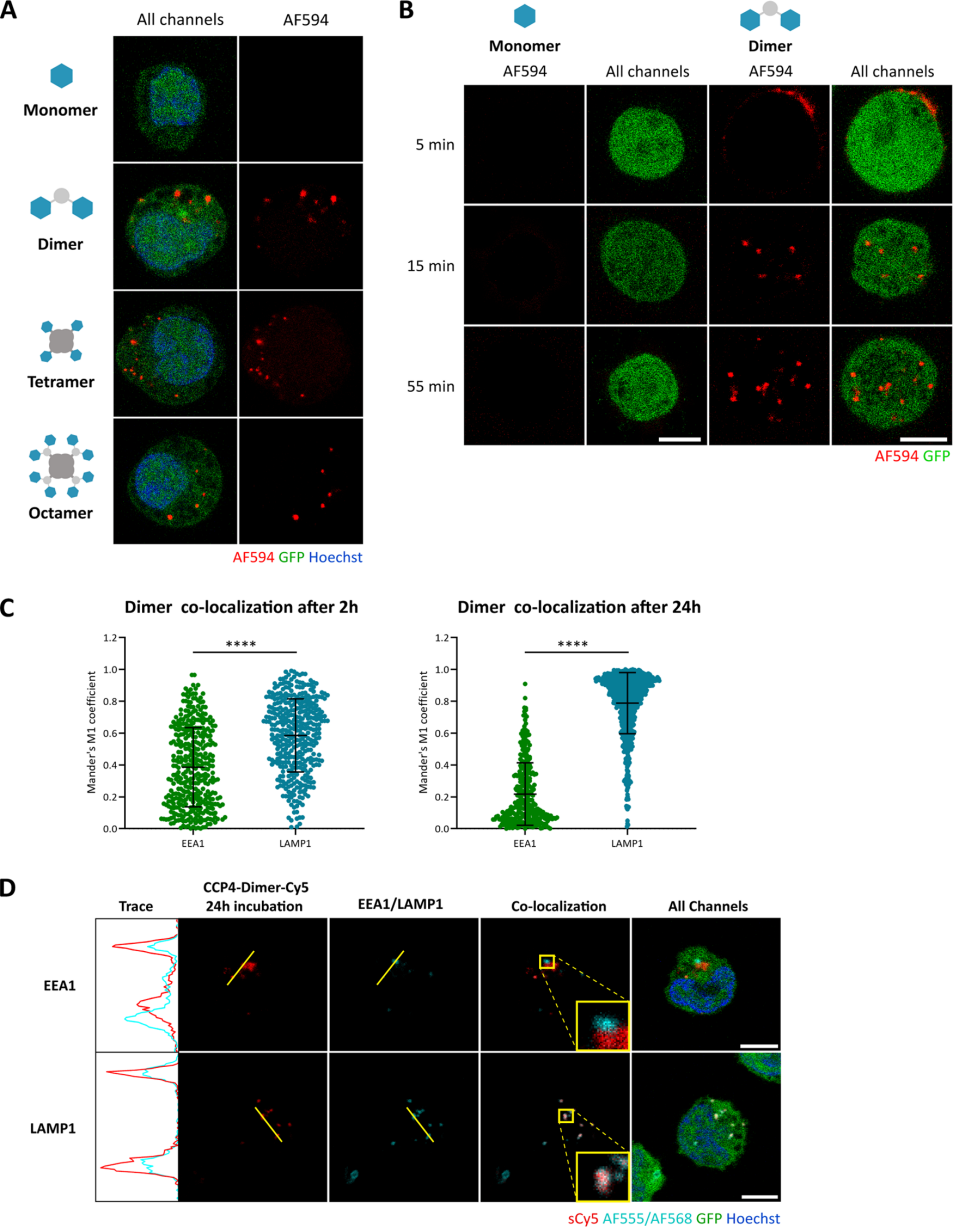
Internalization of the constructs in Ramos 3F3 cells. All constructs were incubated at a CCP4 concentration of 0.6–1 μM. Scale bars represent 5 μm. (A) The dimer, tetramer, and octamer internalize into Ramos 3F3 cells. Constructs were incubated for 10 min at 4 °C and 30 min at 37 °C, before fixation and analysis with confocal microscopy. *N* = 3. (B) The dimer internalizes after approximately 15 min, which is representative of the other constructs, as well. Images of different cells were taken every 10 min from 5 to 55 min in a live-cell imaging setup using confocal microscopy. *N* = 2. (C) EEA1 and LAMP1 colocalization with the dimer-Cy5, as indicated by Mander’s M1 coefficient (amount of dimer-cy5 signal overlapping with EEA1/LAMP1 signal). The dimer was incubated for 2 or 24 h at 37 °C, after which the cells were fixed and immunostained. A minimum of 300 cells was analyzed per condition. 2 h: *N* = 3, 24 h: *N* = 2. **** *p* ≤ 0.0001. (D) Colocalization of the dimer with EEA1 or LAMP1 after 24 h. Representative cells of the quantitative data are shown in (C). *N* = 2.

Next, we investigated the internalization of our constructs into Ramos 3F3 cells using fluorescence confocal microscopy. At 1 μM, we observed internalization into the B cell for the dimer, tetramer, and octamer, while no internalization was observed for the monomer (Figure 3A). None of the constructs showed internalization in the Ramos MDL KO cells (Figure S3A). Internalization of the monomer can possibly occur at higher concentrations, but at this point aggregation effects cannot be ruled out. We see no qualitative difference in internalization between the dimer, tetramer, and octamer (Figure S4A–D). Internalization of the dimer already starts at 10 nM (Figure S3B), making it an attractive delivery module. Next, we investigated the colocalization of the dimer over time with endosomal marker EEA1 and lysosomal marker LAMP1.^36,37^ Colocalization with EEA1 decreased between 2 and 24 h, while colocalization with LAMP1 increased (Figures 3C and S5B,C). The tetramer and octamer showed comparable colocalization with EEA1 as the dimer (Figure S5A).

### Dimer and Octamer Are Less Affected by Antibody Competition

Antigen activation induces the differentiation of mature B cells into antibody-secreting plasma cells. The secreted antibodies bind the targeting antigen, potentially leading to nonspecific Fc receptor-mediated antigen uptake by neighboring cells. We were interested in whether the binding of the constructs to the BCR would be differently affected by secreted antibodies. To investigate this, we used 3F3 antibodies which were produced in mammalian cells and contain the same IgG sequence as the BCR present in the Ramos 3F3 cells.^16^

**Figure 4:**
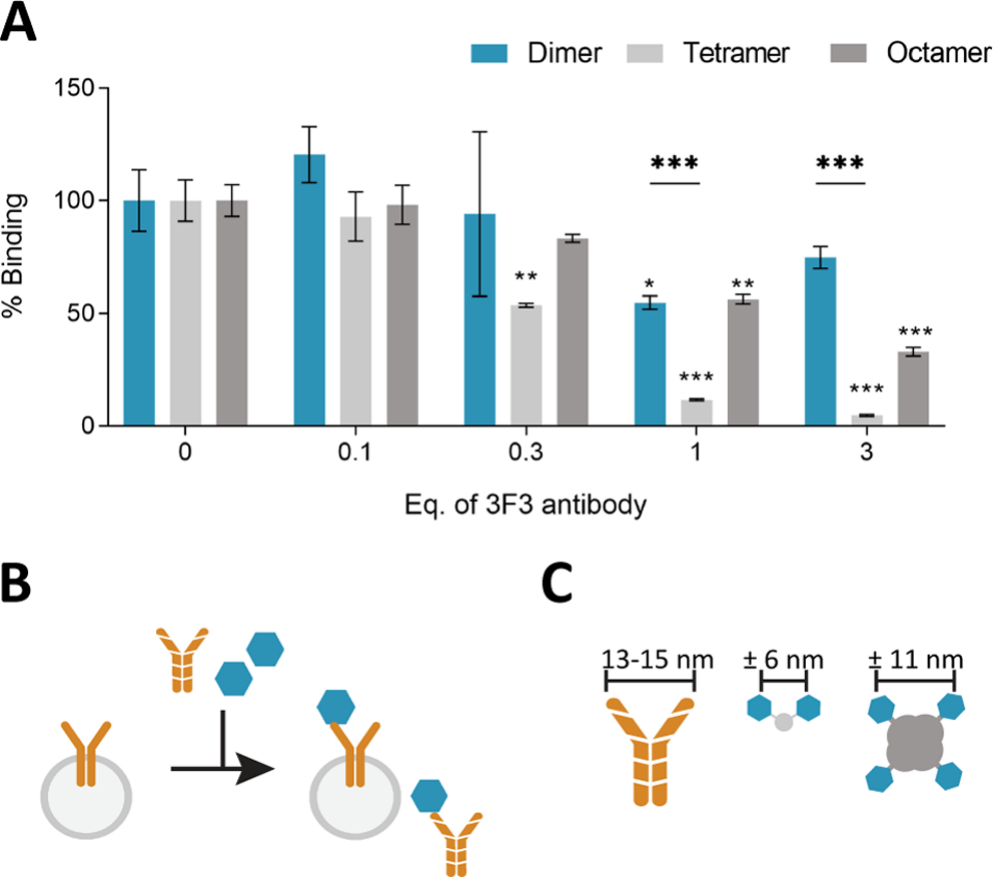
Competition assay with 3F3 antibodies. (A) The dimer and octamer show significantly less competition for BCR binding in the presence of the 3F3 antibody. The * above a bar indicates whether the decrease in binding is significant compared to the non-treated control. * *p* ≤ 0.05, ** *p* ≤ 0.01, *** *p* ≤ 0.001. *N* = 3. (B) The constructs were incubated at increasing equivalents of antibody to CCP4, at 4 μM final CCP4 concentration for 1 h. Next, the samples were diluted to 100 nM CCP4 concentration. They were added to 3F3 Ramos cells which were preincubated with an Fc-blocking reagent, and a binding assay was performed. (C) We hypothesize that the decreased competition of the dimer and octamer with circulating antibodies is caused by the smaller interantigen distance of the dimer that hampers simultaneous binding to a single antibody.

We incubated 3F3 antibodies with our constructs and then performed a binding assay on the Ramos 3F3 cells (Figure 4B), which were preincubated with an Fc-blocking reagent to prevent background binding of the antigen–antibody complexes. We used an increasing concentration of 3F3 antibody of up to 3 equiv, where 1 equiv of antibody equals two CCP4 units. 100 nM CCP4 was used for each condition since this results in saturated binding on the 3F3 cells. The highest concentration of 3F3 antibody (3 equiv, 150 nm) amounts to approximately 45 μg/mL, which is comparable to the average physiological ACPA concentration in patients with RA.^38^

Using this setup, we found that the dimer and octamer are less affected by 3F3 antibody competition than the tetramer. The tetramer showed approximately 50% reduced binding to the BCR at 0.3 equiv of competing 3F3 antibody and no binding at 1 equiv of competing antibody. Interestingly, at 1 equiv competing 3F3 antibody, the dimer and octamer retained approximately 50% of the binding avidity (Figure 4A). We believe this is due to the smaller distance between the two antigens of a dimer compared to a tetramer, which could make the dimer less likely to bind 1:1 to a 3F3 antibody (Figure 4C). We repeated the experiment on Ramos TT control cells to assess the background binding of possible antibody–antigen constructs to, for example, Fc receptors. All conditions showed a maximum of 15% background binding compared to the Ramos 3F3 cells (Figure S6A).

## Discussion

To select the most suitable effector molecule for successful antigen-specific therapy, it is essential to understand the cellular responses to the targeting construct including the binding avidity, BCR-mediated activation of signaling cascades, and receptor internalization. In this study, we systematically investigated the effect of antigen valency on these cellular responses. We synthesized several well-defined low-valency antigens of CCP4 (a monomer, dimer, tetramer, and octamer) and evaluated their properties on a B-cell line expressing an RA patient-derived BCR sequence recognizing a citrullinated antigen. We found that CCP4 multimers decreased the apparent *K*_D_ by approximately 3 orders of magnitude compared to the monomer, indicating a simultaneous engagement of multiple BCRs.^20,21,24^ The binding curve showed two saturation events for the dimer and the octamer. We hypothesize that at lower concentrations, the dimer binds with both ligands to the BCR, resulting in a low apparent *K*_D_ value (*K*_D_1). Once the free dimer concentration is higher than the local concentration of the second ligand, the dimer binds with only one ligand (*K*_D_2).^33,39^ This hypothesis is further confirmed by the observation that the apparent *K*_D_2 of the dimer is comparable to that observed for the monomer. The tetramer displayed only a single saturation point with a similar apparent *K*_D_ as that observed for the *K*_D_1 of the dimer and octamer.

Synthetic constructs with low nM affinities are promising starting points for antigen-specific BCR targeting. They can be connected to effector modules designed for targeted drug delivery or for engagement with inhibitory cell surface receptors to dampen BCR signaling. To make the proper choice of effector molecule, we evaluated the properties of the constructs by directly visualizing the internalization with confocal microscopy. We found that dimeric and higher valent antigens readily internalize into the B cell at low nM concentration within minutes, while no internalization of the monomer was observed at up to 1 μM concentration. Colocalization studies with EEA1 and LAMP1 showed that the constructs are readily transported from the endosomal to the lysosomal compartments which is consistent with previous research investigating internalization of specific antigens.^21,24^ The movement of our constructs to the lysosomes opens possibilities for antigen-specific therapy with for instance lysosome-specific enzyme-cleavable linkers.^40^ In this therapy, antigens are connected to cathepsin-sensitive linker–toxin constructs to selectively eliminate autoreactive B cells after internalization and localization to the lysosomal compartment. Such an approach is currently of high interest to enhance antibody therapy by constructing antibody–drug conjugates for, e.g., cancer treatment.^40^

Consistent with the internalization properties, we also found that multivalent constructs could activate cell signaling through the BCR. At 1 μM, our monomer does not cause an increase in p-PLCγ, whereas the dimer, tetramer, and octamer do to a similar extent. Whether monomeric antigens can activate the BCR is disputed,^21,24,25,41,42^ yet, from the binding experiments, the monomer binds about 10% to the BCR at 1 μM and therefore no or little activation for the monomer is expected at this concentration. At higher concentrations, the monomer might be able to induce p-PLCγ, but aggregation effects cannot be ruled out. B-cell activation may be undesired in immunotherapy since it could potentially enhance an autoimmune reaction, while activation may be beneficial for vaccine development.^43^ It should also be noted that most studies use IgD and IgM-containing B cells, while our cell model expresses an IgG receptor. Übelhart et al. showed that IgM can respond to low-valency antigens, while IgD cannot.^44^ They postulate that IgG might respond similarly since it shares functional similarities with IgD. In RA, the ACPA-specific autoreactive B-cell compartment typically evolves before the onset of symptoms, with the majority of autoreactive B cells expressing IgG.^45,46^

Since we hypothesized that at higher concentrations the dimer binds with only one of its antigen ligands to the BCR, we wondered whether this would also affect the activation level of the BCR. Indeed, we observe that at concentrations above 100 nM CCP4, the level of p-PLCγ induced by the dimer decreases. Previously, a similar effect has been observed for highly multivalent polymers, where the reduced availability of receptors per polymer at high concentrations led to a decrease in activation.^38,39^ However, our tetrameric construct did not exhibit this activation drop at high concentrations, suggesting that this effect is not universal and appears to be dependent on the physical characteristics of the antigen, such as its size and rigidity.

Given the experimental challenges of isolating low abundance CCP-reactive B cells from patient samples, the use of engineered Ramos 3F3 cells as an autoreactive B-cell model greatly facilitates studies to target autoreactive B cells in an antigen-specific manner. Yet, the use of engineered cells overexpressing the BCR, however, warrants caution when translating the results to the complex cellular environment in patients as BCR expression levels and cellular responses may differ.

Finally, we evaluated whether the presence of free-circulating antibodies could potentially interfere with antigen-specific therapy through binding and neutralizing the targeting construct and induce undesired adverse effects by Fc receptor-mediated uptake by neighboring cells.^47^ We found, using a competition experiment, that the dimer and octamer bind less efficiently to free 3F3 antibodies than does the tetramer. We hypothesize that this is due to the different interantigen distances. A streptavidin tetramer has a diameter of approximately 11 nm and IgG is estimated to have a binding-site distance of 13–15 nm due to the flexibility of its arms.^48,49^ A tetramer could, therefore, bind 1:2 with a 3F3 antibody (Figure 4B). Our dimer has a maximum interantigen distance of approximately 6 nm (Figure S6B) making a 1:1 binding with the two arms of an antibody troublesome. It is likely that the dimer binds with only one arm to the free antibody, reducing the avidity due to the lack of multivalency. These results suggest that a multimer with a smaller interantigen distance could increase targeting effectiveness and decrease unspecific Fc-mediated cellular uptake.

## Conclusions

In conclusion, we synthesized a well-defined monomer and dimer of the CCP4 antigen. By combining a biotin monomer and dimer with streptavidin, we also made CCP4 tetramers and octamers. Binding studies showed that the dimer, tetramer, and octamer have a significantly higher avidity to the BCR compared to the monomer, are readily internalized, and traffic toward the lysosome. Furthermore, the dimer is less affected by competition with circulating antibodies than the tetramer and octamer. We envision that the triggered release of a potent effector molecule from our CCP4 dimer would be an attractive strategy for selective elimination of autoreactive B cells.

## Supporting Information Available

Supporting Information Figures S1–S7; Supporting Information Tables S1–S2; detailed synthesis procedures; NMR, HPLC and LCMS data (PDF)
