# Supplementary material for: Effect of Antigen Valency on Autoreactive B-Cell Targeting
Source: Mol Pharm. 2023 Oct 20;21(2):481–90. doi: 10.1021/acs.molpharmaceut.3c00527 (PMC10848265; doi:10.1021/acs.molpharmaceut.3c00527)

Supplementary material for

The effect of antigen valency on autoreactive B-cell targeting

M.J. van Weijsten[1,4†], K.R. Venrooij[1,4†], L.P.W.M. Lelieveldt[1,4], T. Kissel[2], E. van Buijtenen[1], F.J. van Dalen[3,4], M. Verdoes[3,4], R.E.M. Toes[2], K.M. Bonger[1,4*]

†These authors contributed equally to this work

* To whom correspondence should be addressed: Heyendaalseweg 135, 6525 AJ, Nijmegen, The Netherlands. Telephone: +31 243652535. E-mail: kim.bonger@ru.nl

[1]Institute for Molecules and Materials, Radboud University, Heyendaalseweg 135, 6525 AJ Nijmegen, The Netherlands

[2]Department of Rheumatology, Leiden University Medical Center, Albinusdreef 2, 2333 ZA Leiden, The Netherlands

[3]Department of Medical BioSciences, Radboudumc, Geert Grooteplein Zuid 28, 6525 GA Nijmegen, The Netherlands.

[4]Institute for Chemical Immunology, Nijmegen, The Netherlands.

This document includes:

1. Supplemental Figures S1-S7
2. Supplemental Tables S1-S2
3. Detailed synthesis procedures
4. NMR, HPLC and LCMS data

1. Supplemental figures

Figure S1

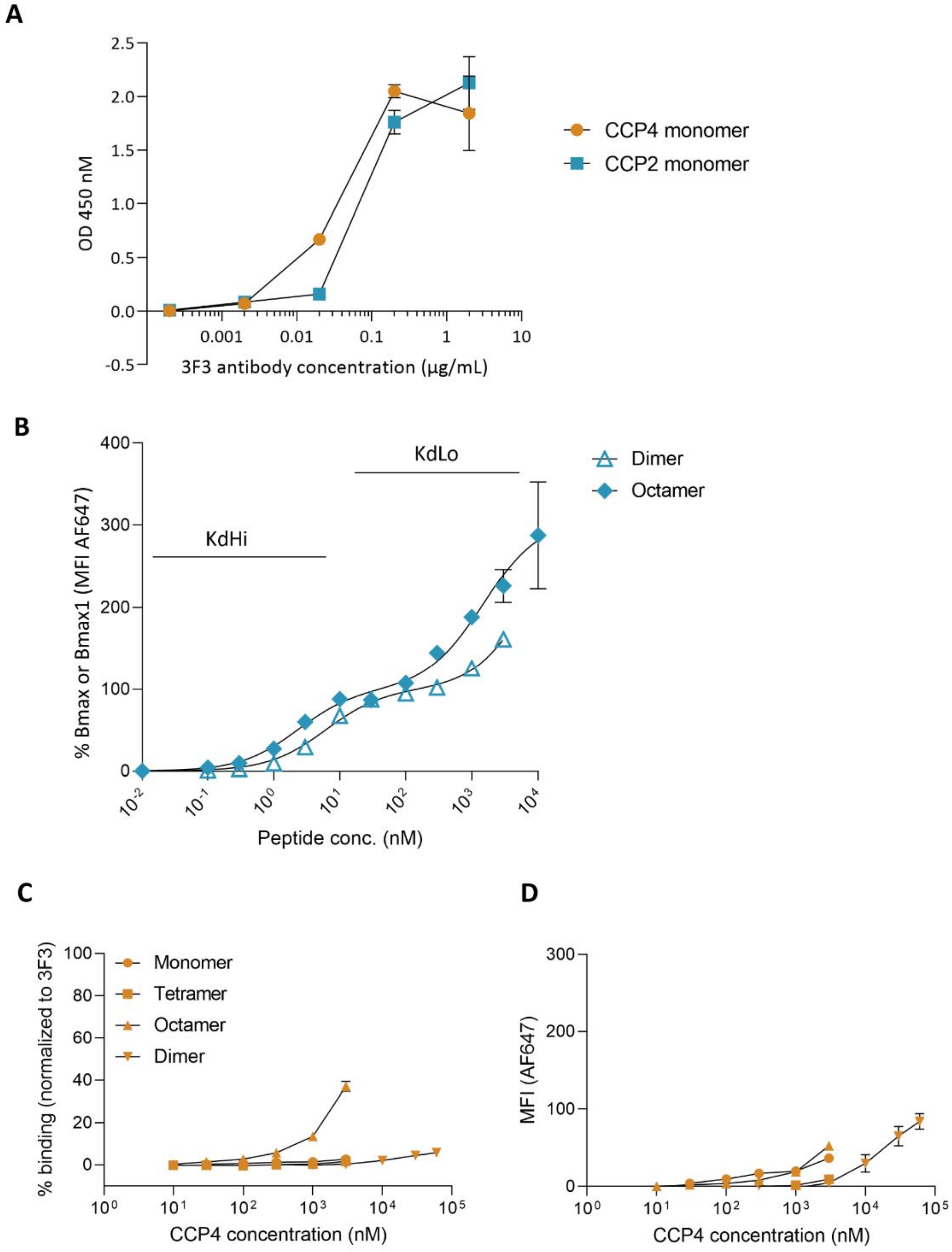

Figure S1: Specific binding of the CCP4 constructs. **A.** CCP4 monomer binds to 3F3 antibodies with a comparable affinity to CCP2 monomer. Binding was measured with ELISA. Plate were coated with peptide and incubated with 3F3 antibody for 1 hour at 37°C. N=3. **B.** The dimer and octamer follow a two-step saturation curve, resulting in a K_D1and K_D2 value (see sup fig 1). **C. and D.** The CCP4 constructs do not bind significantly to the MDL KO cells. The constructs were incubated for 15 min at 4°C. N=3.

Figure S2

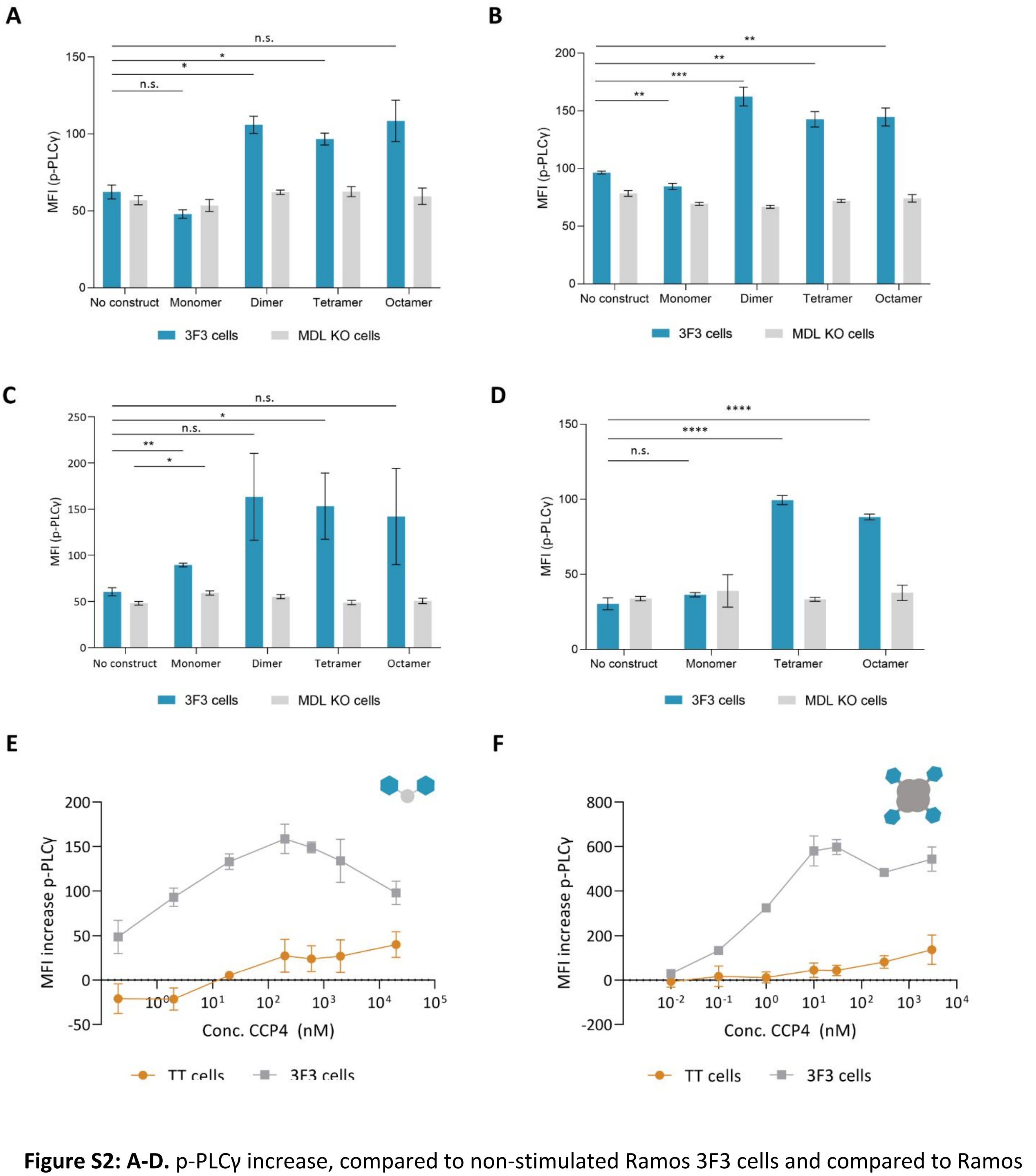

Figure S2: A-D. p-PLCγ increase, compared to non-stimulated Ramos 3F3 cells and compared to Ramos MDL KO cells. Five individual experiments, N=3. If not indicated, p-PLCγ in- or decrease is not significant compared to non-treated cells. All constructs were incubated at 1 μM peptide concentration, 10 min at 4°C and 15 min at 37°C. **E and F.** p-PLCγ increase of Ramos TT cells compared to 3F3 cells, stimulated with CCP4 dimer or tetramer respectively.

Figure S3

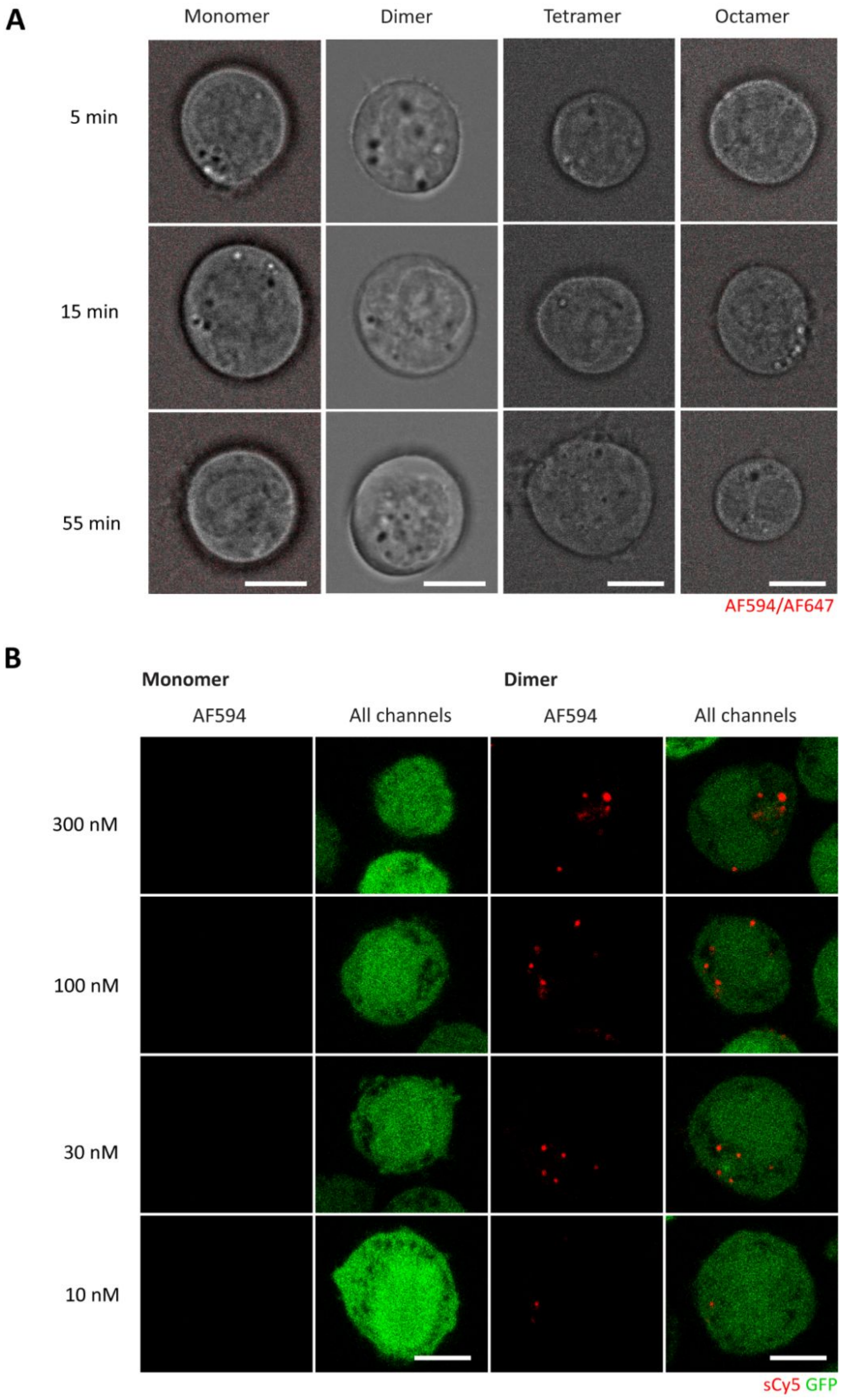

Figure S3: A. No visible internalization in Ramos MDL KO cells for all constructs. Constructs were incubated at 1 µM. Images of different cells were taken every 10 min from 5-55 min in a live-cell imaging setup. **B.** The CCP4 monomer does not internalize from 10-300 nM. The CCP4 dimer internalises from 10 nM onwards. The constructs were incubated 10 min at 4°C and 30 min at 37°C, before fixation and analysis.

Figure S4

A

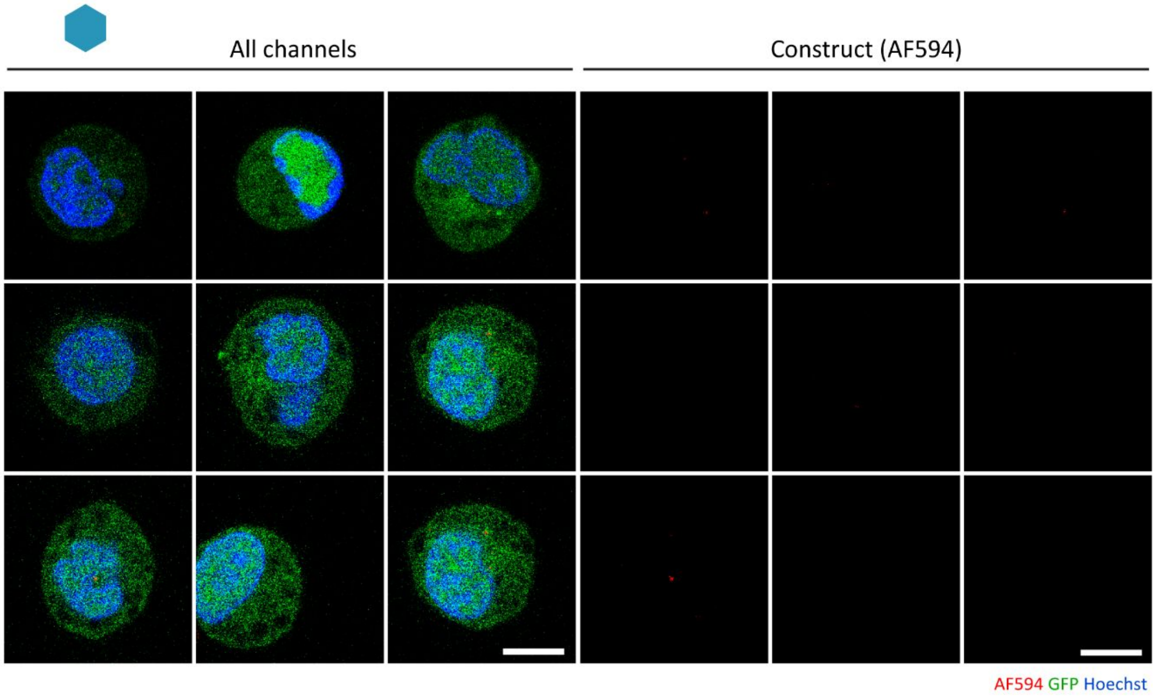

All channels
Construct (AF594)
AF594 GFP Hoechst

B

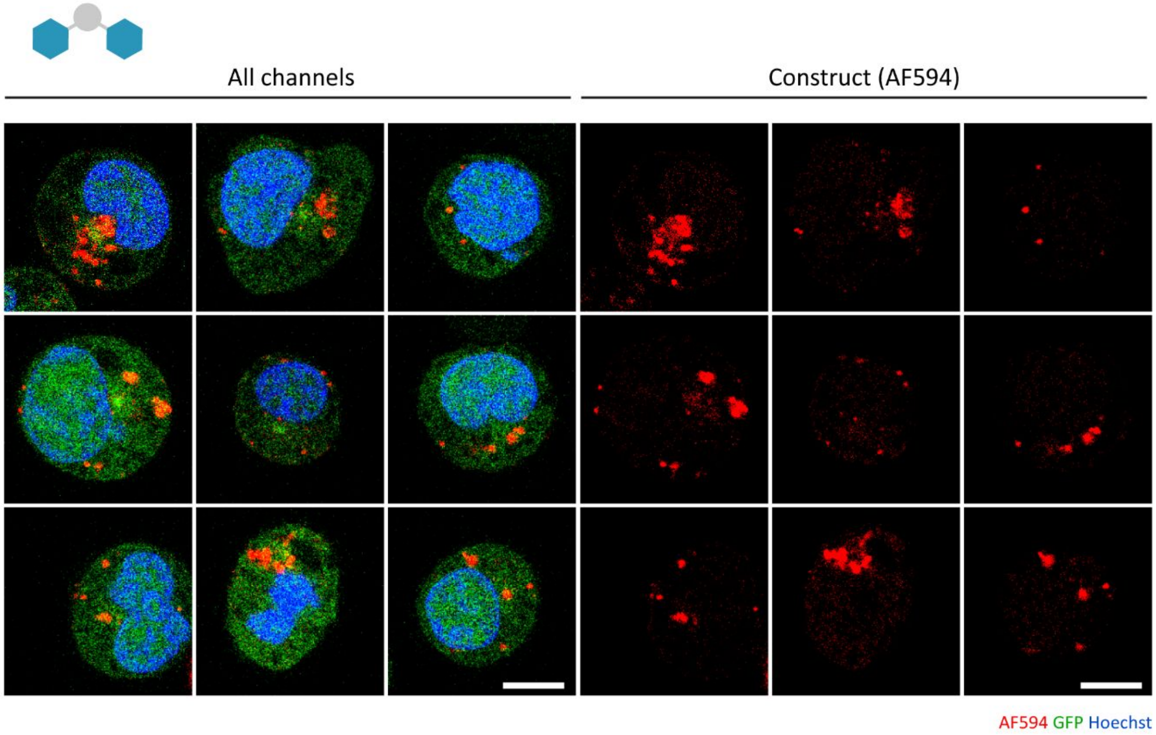

All channels
Construct (AF594)
AF594 GFP Hoechst

C

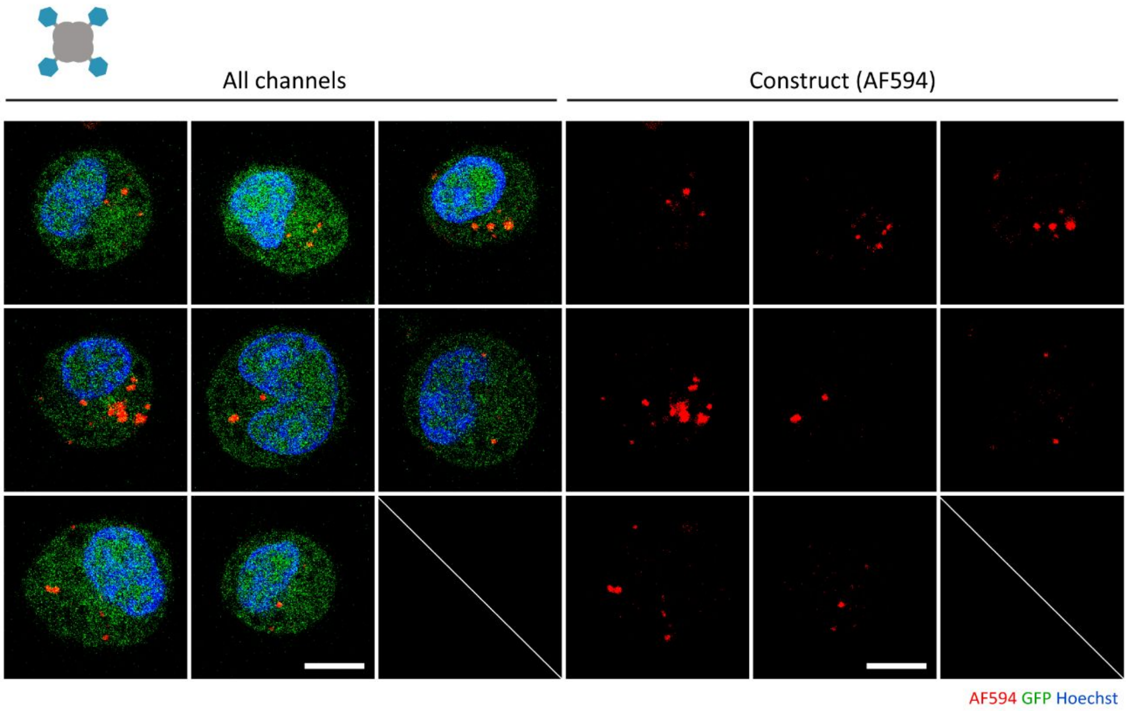

D

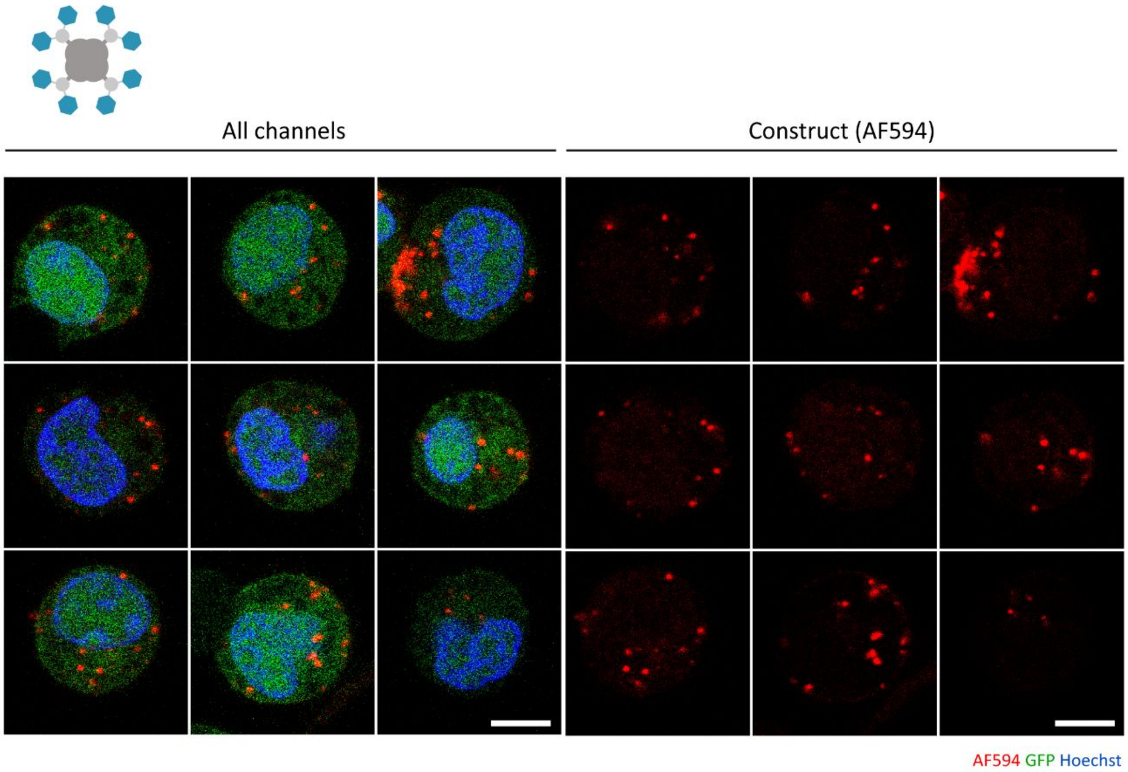

Figure S4: All multimeric constructs show similar internalization. All constructs were incubated at 1 µM CCP4 concentration, 10 min at 4°C and 30 min at 37°C, before fixation and analysis with confocal microscopy. Slices shown here represent the center of a z-stack. N=3. Scale bars represent 5 µm. **A.** Monomer **B.** Dimer **C.** Tetramer **D.** Octamer

Figure S5

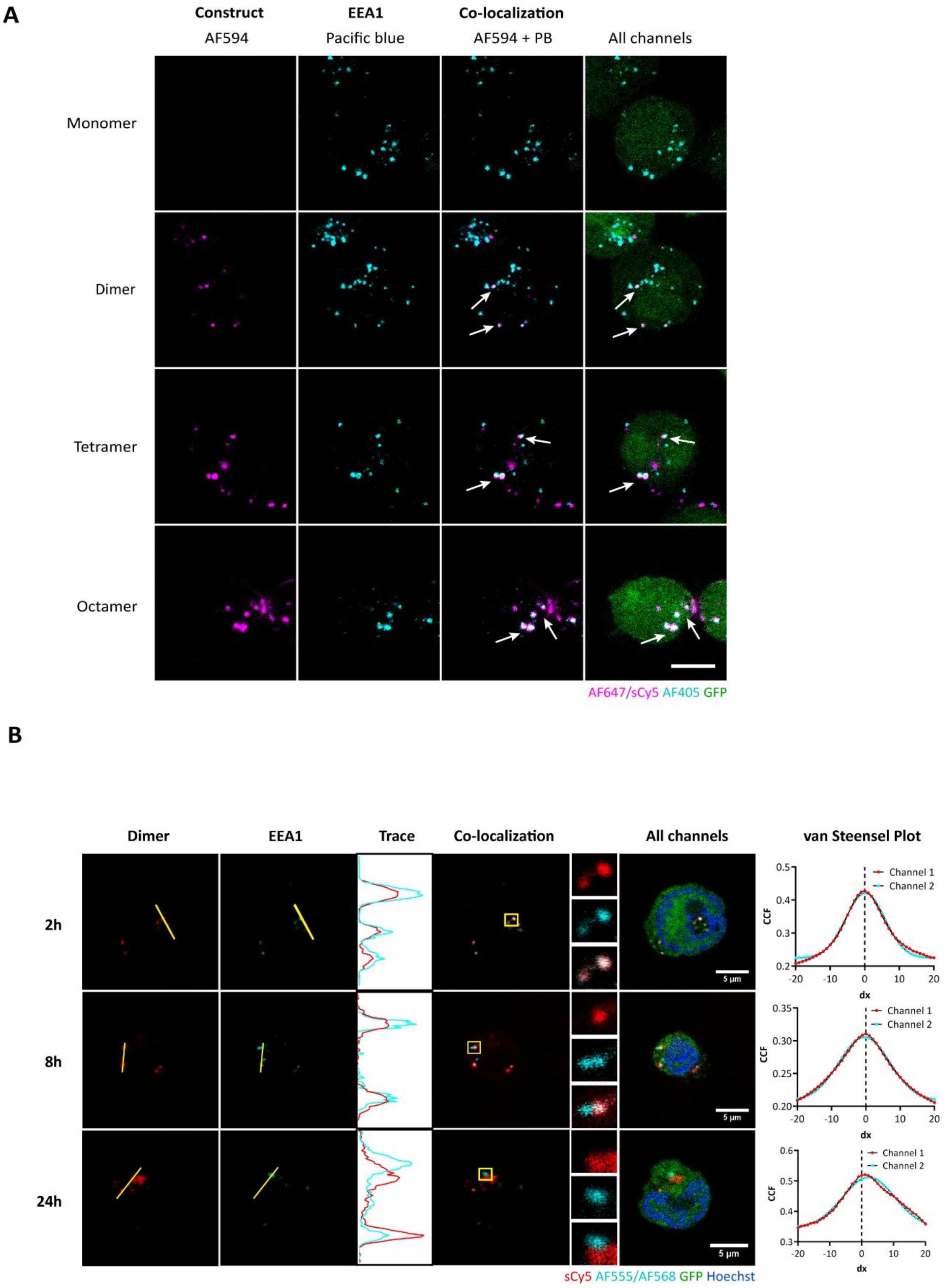

A
Construct
AF594
EEA1
Pacific blue
Co-localization
AF594 + PB
All channels
Monomer
Dimer
Tetramer
Octamer
AF647/sCy5 AF405 GFP
B
Dimer
EEA1
Trace
Co-localization
All channels
van Steensel Plot
2h
8h
24h
5 µm
Channel 1
Channel 2
CCF
dx
sCy5 AF555/AF568 GFP Hoechst

C

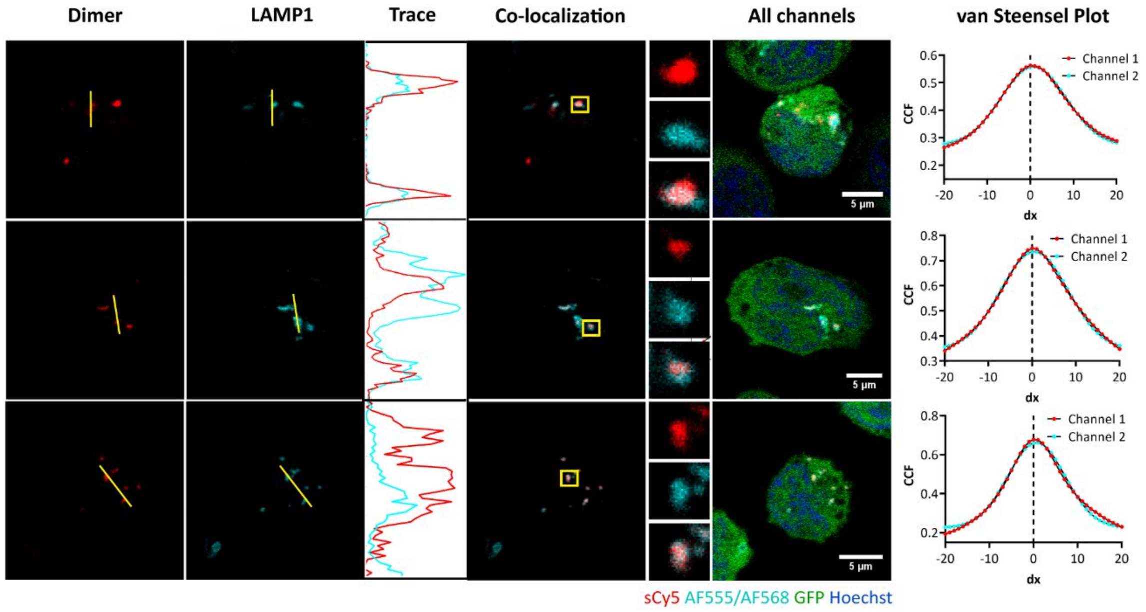

Figure S5: Scale bars represent 5 µm. **A.** Co-localisation of the CCP4 constructs with endosomal marker EEA1 and lysosomal marker LAMP1. **A.** After 30 min, the dimer, tetramer, and octamer co-localise with endosomal marker EEA1 (white arrows). All constructs were incubated at 0,6-1 µM. **B.** Co-localisation of the CCP4 dimer with EEA1. Co-localisation decreases over time. **C.** Co-localisation of the CCP4 dimer with LAMP1. Co-localization increases over time.

Figure S6

A

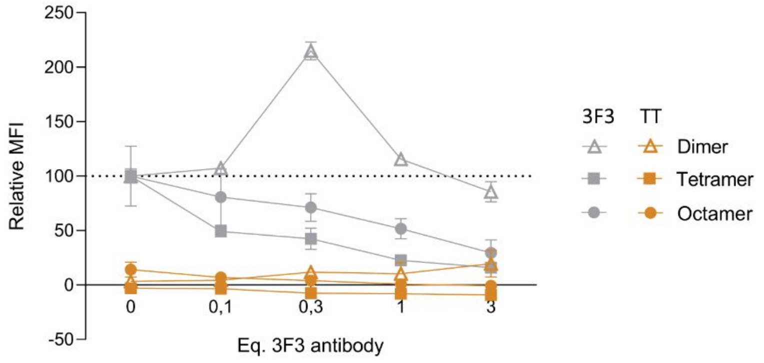

B

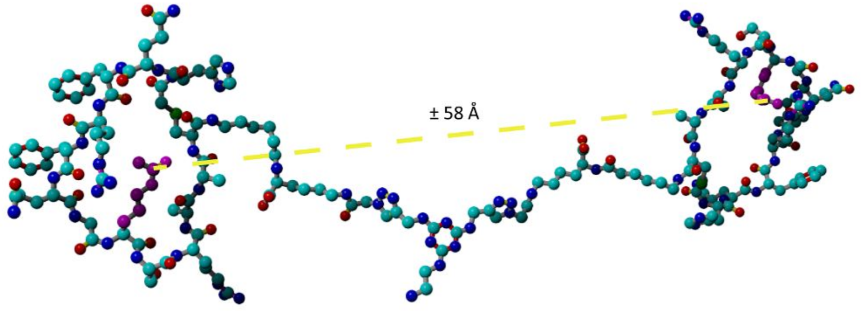

Figure S6: A. Competition assay with 3F3 antibodies on TT cells. The constructs were incubated 1:1 CCP4:Eq of 3F3 binding site, at 4 µM for 1h. Next the samples were diluted to 100 nM, added to TT Ramos cells and a binding assay was performed. The TT cells show no background binding of the antibody-antigen construct. **B.** 3D model of the CCP4 dimer. The distance between the two citrullines of each antigen is approximately 58 Å, or 5.8 nm. Purple = citrullines. The model was generated with Yasara.

Figure S7

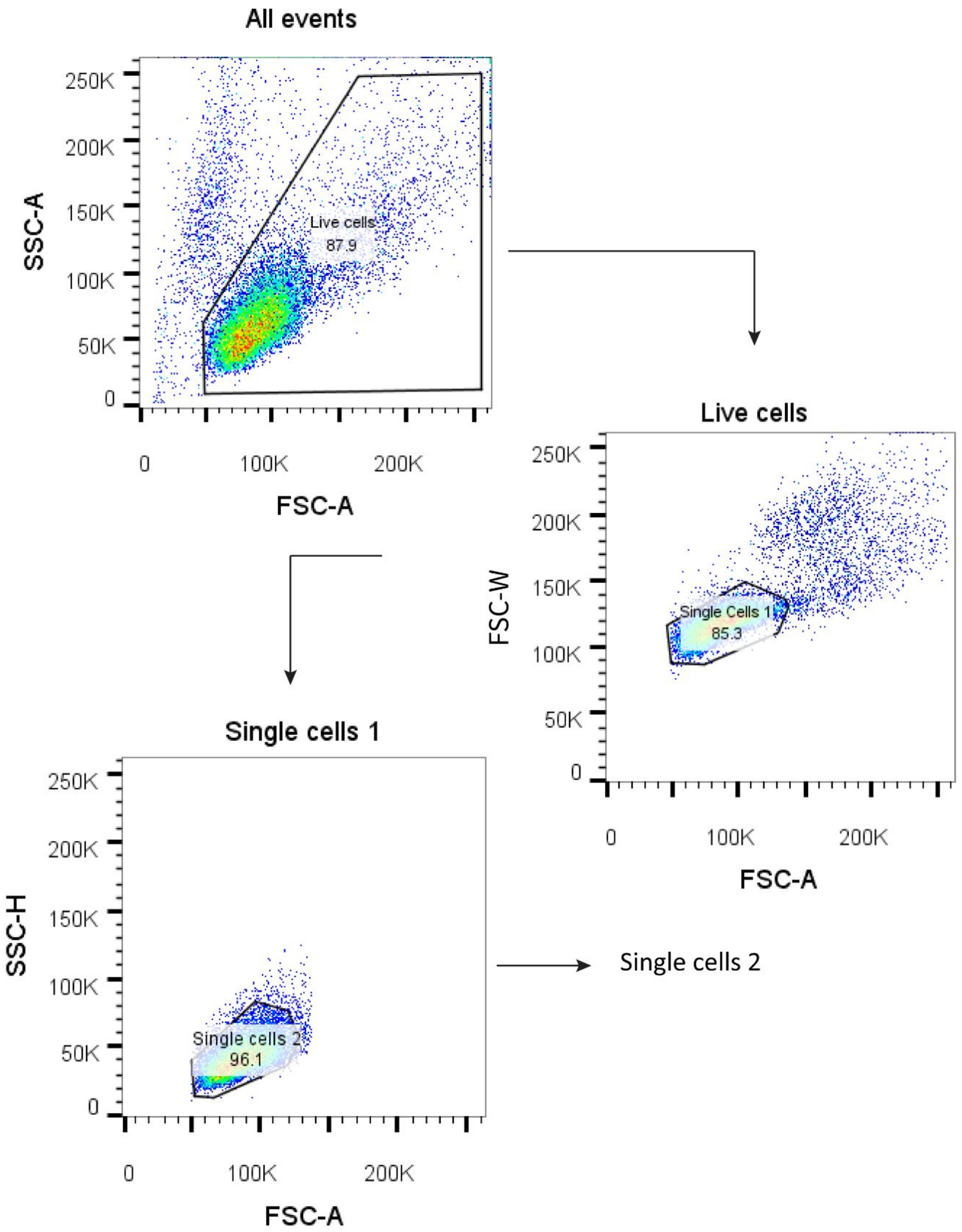

Figure S7: General FACS gating procedure. All events were first gated for live cells (FSC-A vs SSC-A). Next, single cells were isolated in two steps, using FSC-A vs FSC-W and FSC-S vs SSC-H. The resulting cell population was used for further analysis.

2. Supplemental tables

Table S1: Sequences used for the generation of Ramos 3F3 and TT cell lines. Table is partly derived from Kissel et al. [1]

	IGH-CDR3aa*	IGHV*	%Id*	IGHD*	IGHJ*	IGL-CD3aa*	LC	IGKV*	% Id*	IGLJ*
3F3	CARGTYLPVDE SAAFDVW	IGHV1-2*02	80.56 %	IGHD2-8*01	IGHJ3*01	CQQYYEAPY TF	κ	IGKV4-1*01	87.54%	IGKJ2*01
TT	ARHVLITFG GLIEQTYYF DS	IGHV4-39*03	72.41 %	IGHD2-21*02	IGHJ2*01	MGGLQTPT	κ	IGKV2-28*01 or IGKV2D-28*01	69,39%	IGKJ2*01 or IGKJ2*02

*Determined by IMGT/V-QUEST

Table S2: CCP4:fluorophore ratios for the CCP4 constructs.

	CCP4	Fluorophore	Fluorophore/CCP4
**Monomer**	1	1	1
**Dimer**	2	1	0,5
**Tetramer**	4	1	0,25
**Octamer**	8	1	0,125

3. Detailed synthesis procedures

General methods and materials

Amino acids were obtained from Bachem (Bubendorf, Switzerland) or Novabiochem (EMD Chemical, Gibbstown, USA). Solvents were purchased from J.T. Baker, Biosolve, Fisher Scientific and Merck Milipore and were used as received.

Analytical thin layer chromatography (TLC) was performed on silica gel-coated plates (Merck, 60 F254) with the indicated solvent mixture, and visualization done with ultraviolet (UV) irradiation (λ = 254 nm) and/or staining with aqueous $KMnO_4$ or ninhydrin, acetic acid in acetone and gentle warming. Compounds purified by column chromatography was performed with silica gel 60 (Merck, 0.040-0.063 mm).

NMR chemical shifts (δ) were recorded on a Bruker Avance III 400 MHz spectrometer equipped with a Prodigy BB cryoprobe. ^1H NMR chemical shifts (δ) are reported in parts per million (ppm) relative to a residual proton peak of the solvent, i.e. $CDCl_3$ (δ = 7.26), $(CD_3)_2SO$ (δ = 2.50) or D_2O (δ = 4.79). Coupling constants are reported as a J-value in Hertz (Hz). ^{13}C NMR chemical shifts (δ) are reported in parts per million (ppm) relative to a residual proton peak of the solvent, i.e. $CDCl_3$ (δ = 77.16) or $(CD_3)_2SO$ (δ = 39.52).

A Thermo finnigan LCQ Fleet ESI ion-trap mass spectrometer with a Shimadzu HPLC (C18 column, particle size 3 µm, 5-100% acetonitrile/water gradient, 16 min with a flow of 0.2 mL/min) and a PDA detector, was used for separation of organic compounds and measurement of low-resolution mass spectra (LRMS). The high-resolution mass spectra (HRMS) were recorded on a JEOL AccuTOF JMST100CS (ESI).

Preparative HPLC was performed on a Shimadzu LC-20A Prominence system (Shimadzu, ‘s-Hertogenbosch, the Netherlands) with a Gemini NX-C18 column, 150 x 21.2 mm, particle size 10 µm (Phenomenex, Utrecht, the Netherlands). A gradient of 5-35% acetonitrile/water + 0.1% trifluoroacetic acid, in 40 min with a flow of 6 mL/min was used. The analytical HPLC measurements were done on a Shimadzu LC-20A Prominence system (Shimadzu, ‘s-Hertogenbosch, the Netherlands) with a Gemini NX-C18 column, 150 x 3 mm, particle size 3 µm (Phenomenex, Utrecht, the Netherlands). The gradient was 5/100% acetonitrile/water, in 30 min with a flow of 0.4 mM/min. The products were observed at 214 and 254 nm, with the desired peaks being manually integrated using the LabSolutions software package (Shimadzu, ‘s-Hertogenbosch, the Netherlands)

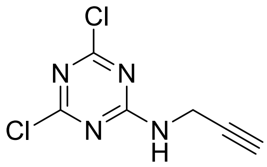

4,6-Dichloro-N-(prop-2-yn-1-yl)-1,3,5-triazin-2-amine (1). Cyanuric chloride (2.00 g, 10.85 mmol, 1 eq.) was dissolved in 30 mL THF:DCM (2:8) and cooled to 0°C. Subsequently, a solution of DIPEA (2.3 mL, 13.02 mmol, 1.2 eq) and propargylamine (0.69 mL, 10.85 mmol, 1 eq.) in 30 mL THF:DCM (2:1) was added dropwise over 3 hours. The mixture was then stirred for 18 hours at 0°C. Subsequently, the solvent was removed in vacuo and purified using column chromatography (40% EtOAc in heptane to 100% EtOAc), affording a white powder (1.43 g, 7.05 mmol, 65%). ^{1}H NMR (400 MHz, $CDCl_3$) δ 6.42 (s, 1H), 4.30 (dd, *J* = 5.7, 2.5 Hz, 2H), 2.31 (t, *J* = 2.5 Hz, 1H). ^{13}C NMR (126 MHz, $CDCl_3$) δ 171.39, 170.40, 165.68, 77.41, 76.90, 73.04, 31.47. Data agrees with reference spectra. [2]

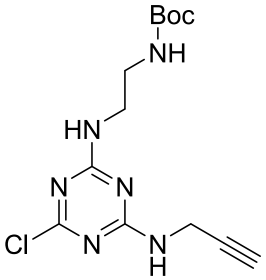

Tert-butyl (2-((4-chloro-6-(prop-2-yn-1-ylamino)-1,3,5-triazin-2-yl)amino)ethyl)carbamate (2).

Triazin **1** (1.30g, 6.41 mmol, 1.0 eq) was dissolved in anhydrous DMF (23.2 mL) at RT. Subsequently, DIPEA (1.24 mL, 7.11 mmol, 1.11 eq) and N-Boc ethylenediamine (1.11 mL, 7.05 mmol, 1.1 eq) were added. The mixture was then heated at 60°C for 18h and then concentrated *in vacuo*. Finally, a white powder (1.60g, 4.90 mmol, 76%) was obtained after purification by column chromatography (10% EtOAc in heptane to 100% EtOAc). Rf (1:1 EtOAc:Heptane) 0.5. ^{1}H NMR (400 MHz, DMSO) δ 8.12 (dt, *J* = 29.9, 5.9 Hz, 1H), 7.86 (dt, *J* = 12.2, 5.8 Hz, 1H), 6.82 (q, *J* = 6.1 Hz, 1H), 4.07 (dd, *J* = 5.9, 2.4 Hz, 1H), 3.99 (dd, *J* = 5.8, 3.0 Hz, 1H), 3.25 (dq, *J* = 11.4, 6.0 Hz, 2H), 3.13 – 3.01 (m, 3H), 1.36 (s, 9H). ^{13}C NMR (101 MHz, DMSO) δ 167.81, 165.37, 165.13, 155.59, 80.92, 77.65, 73.85, 72.94, 40.33, 29.64, 27.71. LRMS (ESI+) m/z calculated for $C_{13}H_{19}ClN_6O_2$ $[M+H]^+$ 327.13 , found 326.84.

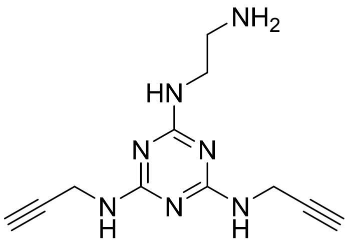

N2-(2-aminoethyl)-N4,N6-di(prop-2-yn-1-yl)-1,3,5-triazine-2,4,6-triamine (3). Triazin **2** (98.0 mg, 0.30 mmol, 1.0 eq) was dissolved in anhydrous DMSO (2 mL) at RT. Subsequently, DIPEA (0.1 mL, 0.6 mmol, 2 eq) and propargyl amine (0.29 mL, 4.5 mmol, 15 eq) were added. The mixture was heated at 80°C for 4h and subsequently concentrated *in vacuo*. The reaction mixture was diluted with 6 ml DCM. Then, TFA (6 ml) was slowly added. The reaction was stirred at room temperature for 2 hours and 15 minutes. Next, the reaction mixture was co-evaporated with chloroform and concentrated *in vacuo*. The product was purified using automated flash column chromatography (reverse phase, 0 to 100% ACN in MQ + 0.1% TFA, 30 ml/min), product fractions were lyophilized and N2-(2-aminoethyl)-N4,N6-di(prop-2-yn-1-yl)-1,3,5-triazine-2,4,6-triamine (60.3 mg, 0.176 mmol, 58.6%) was obtained as a light-yellow solid. Rf-value: 0.24 (1% Et_3N in 10% MeOH in DCM). ^{1}H NMR (400 MHz, D_2O) δ 4.35 – 4.12 (m, 4H), 3.92 – 3.66 (m, 2H), 3.34 – 3.20 (m, 2H), 2.76 – 2.60 (m, 2H). ^{13}C NMR (101 MHz, D_2O) δ 163.05, 162.69, 72.52, 39.13, 38.30, 29.94. LRMS (ESI+) m/z calculated for $C_{10}H_{16}N_7$ $[M+H]^+$ 246.29, found 246.30.

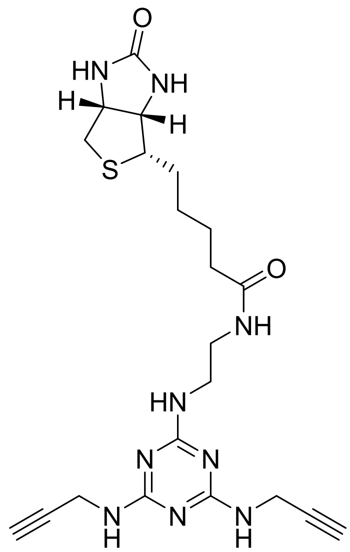

N2-(2-aminoethyl)-N4,N6-di(prop-2-yn-1-yl)-1,3,5-triazine-2,4,6-triamine (4). The triazin **3** (37.23 mg, 104.2 µmol, 1 eq) was dissolved in anhydrous DMF in a flame-dried round bottom flask under argon, filled with activated mol sieves (3A). DIPEA (89.5 µL, 67.34 mg, 0.521 mmol, 5 eq) was then added and the solution was left overnight under argon, stirring at 100 rpm. The following day, Biotin-NHS (43.0 mg, 0.126 µmol, 1.21 eq) was added to the reaction vessel. After 2 hours the reaction mixture was filtered and diluted with 3 mL MQ. After stirring for 30 min at 700 rpm, the reaction mixture was concentrated *in vacuo* to yield an off-white solid (33.2 mg). This product was used as crude in the subsequent reaction. LCMS (ESI+) m/z calcd for $C_{21}H_{30}N_9O_2S$ $[M+H]^+$ 472.22, found 472.48.

Synthesis of CCP4 (5). Wang resin was swollen for 3 hours in NMP. Subsequently, Fmoc-Lys(Boc)-OH (3 eq) and HOBt (4 eq) were dissolved in NMP and added to the resin. A solution of DMAP (2 eq) and DIPCDI (2 eq) in NMP was prepared and also added to the resin. After allowing the resin to react overnight, it was washed 3x with NMP and dried by washing with DCM (3x), MeOH (3x), DCM (3x) and MeOH (3x), after which the resin was further dried in a vacuum oven overnight and the loading was determined by weighing the resin. The resin was subsequently swollen in NMP for 3 hours and benzoyl chloride (0.34 mL/g resin) and pyridine (0.28 mL/g resin) in NMP was added for 30 minutes. The resin was then washed with DCM (3x), NMP (3x), DCM (3x) and NMP (3x).

Fmoc protecting groups were removed by addition of piperidine in NMP (20%, v/v) and leaving it to shake for 20 minutes. The resin was then washed 3 times with NMP and fmoc removal was verified using a Kaiser test. Consequently, the proper amino acid (3 eq) was dissolved in NMP with Nhydroxybenzotriazole (HOBt 3.6 eq) and N,N'-diisopropylcarbodiimide (DIPCDI, 3.3 eq) and added to the resin. Coupling lasted until completion and was monitored using a Kaiser test. If no completion was observed within 2 hours, a new mixture of amino acid, HOBt and DIPCDI was added. Difficult couplings, such as Fmoc-6-Ahx-OH, were performed with HATU (2.88 eq) and DIPEA (6 eq). For regular amino acids, hard couplings were performed overnight with HOBt/DIPCDI. All couplings with Fmoc-Arg(Pbf)-OH were done in DCM instead of NMP. Upon completion of a coupling the resin was washed three times with NMP and subsequently acetic anhydride (1 mL) and pyridine (1 mL) in NMP (12 mL) was added to the resin for 1 minute. The resin was washed three times with NMP and the cycle was repeated until the last amino acid was coupled. After Fmoc deprotection, chloroacetic anhydride (5 eq) and DIPEA (10 eq) were added.

After the final coupling, the resin was washed with DCM (3x), methanol (3x), DCM (3x), methanol (3x) and dried. For cleavage, the resin was first washed with DCM for 2 hours. A cleavage cocktail of TFA/TIPS/1,2-Ethanedithiol (EDT) (92.5:3.75:3.75) was freshly prepared and bubbled through with Argon gas. After 3 hours of suspending the resin in the cleavage cocktail, the peptide was obtained by precipitation in diethyl ether (-10°C) of the filtrate. The obtained peptide was then lyophilized and cyclized in a buffer (2 mg peptide/mL, pH 8.4) of H2O and MeCN (1:1) containing NH_4HCO_3 (50 mM) overnight. The buffer was then concentrated, lyophilized and peptides were purified using a reversed phase preparative HPLC.

HPLC: Rt. 15.133 min. HRMS (ESI+) m/z calcd for $C_{81}H_{128}N_{27}O_{20}S$ $[M+H]^+$ 1830.95496, found 1830.95469.

CCP4-N_3 (6). To a flame dried flask under argon was added 3.5 mL dry DMF, CCP4 **5** (80% pure, 35.0 mg, 19 µmol, 1 eq) and 2,5-dioxopyrrolidin-1-yl 2-azidoacetate (3.8 mg, 19 µmol, 1 eq). Subsequently anhydrous DIPEA (33 µL, 0.19 mmol, 10 eq) was added and the mixture was left to stir at 1400 rpm for 45 min. The reaction mixture was then concentrated *in vacuo*, diluted with 6 mL MQ:ACN:TFA (8:2:0.1) and spun down (4500 x *g*, 5 min, RT) and the supernatant was injected in the RP HPLC (3 runs). The supernatant was purified by RP HPLC and product fractions were concentrated *in vacuo* and lyophilized, yielding a white fluffy powder (18.5 mg, 51%). HPLC: Rt. 16.408 min. LCMS (ESI+) m/z calcd for $C_{83}H_{129}N_{30}O_{21}S$ $[M+H]^+$ 1913.966376, found 1914.88. $C_{83}H_{130}N_{30}O_{21}S$ $[M+2H]^{2+}$ 957.486826, found 957.68. $C_{83}H_{131}N_{30}O_{21}S$ $[M+3H]^{3+}$ 638.660309, found 639.04. HRMS (ESI+) m/z calcd for $C_{83}H_{129}N_{30}O_{21}S$ $[M+H]^+$ 1913.96692, found 1913.97036.

CCP4-Biotin (7). To a flame dried flask under argon was added 2 mL dry DMF, CCP4 **5** (80% pure, 10.01 mg, 5.47 µmol, 1 eq) and biotin-NHS (1.83 mg, 5.36 µmol, 0.98 eq). Subsequently anhydrous DIPEA (2.8 µL, 16.4 µmol, 3 eq) was added and the mixture was left to stir at 1400 rpm for 45 min. The reaction mixture was then diluted with 6 mL MQ:ACN:TFA (8:2:0.1), centrifuged (4500 x *g*, 5 min, RT) and the supernatant was injected in the RP HPLC. Product fractions were concentrated *in vacuo* and lyophilized, yielding a white fluffy powder (7.00 mg, 3.40 µmol, 62.2%). HPLC: Rt. 16.256 min. LCMS (ESI+) m/z calcd for $C_{91}H_{143}N_{29}O_{22}S_2$ $[M+2H]^{2+}$ 1029.019646, found 1029.84. $C_{91}H_{144}N_{29}O_{22}S_2$ $[M+3H]^{3+}$ 686.348856, found 686.92. HRMS (ESI+) m/z calcd for $C_{91}H_{142}N_{29}O_{22}S_2$ $[M+H]^+$ 2057.03256, found 2057.02729.

CCP4-AF594 (8). To a flame dried flask under argon was added 1 mL dry DMSO, CCP4 **5** (100% pure, 5.8 mg, 3.2 µmol, 1 eq) and AF594-NHS (2.5 mg, 3.1 µmol, 1 eq). Subsequently anhydrous DIPEA (5.3 µL, 0.31 mmol, 10 eq) was added and the mixture was left to stir at 1400 rpm for 1.5h in the dark. The reaction mixture was then diluted with 2 mL MQ:ACN:TFA (8:2:0.1), centrifuged (4500 x *g*, 5 min, RT) and the supernatant was injected in the RP HPLC (1 run). Product fractions were concentrated *in vacuo* and lyophilized, yielding a purple fluffy powder (2.50 mg, 0.99 µmol, 32%). HPLC: Rt. 18.123 min. LCMS (ESI+) m/z calcd for $C_{116}H_{161}N_{29}O_{30}S_3$ $[M+2H]^{2+}$ 1268.06, found 1268.76. $C_{116}H_{162}N_{29}O_{30}S_3$ $[M+3H]^{3+}$ 845.71, found 846.24. HRMS (ESI+) m/z calcd for $C_{116}H_{159}N_{29}Na_2O_{30}S_3$ $[M+2Na]^{2+}$ 2580.07651, found 2580.07838.

CCP4-SCy5 (9). To a flame dried flask under argon was added 2 mL dry DMSO, CCP4 **5** (80% pure, 29.8 mg, 13.02 µmol, 1 eq) and SCy5-NHS (12.0 mg, 16.2 µmol, 1.25 eq). Subsequently DIPEA (29 µL, 0.17 mmol, 10 eq) was added and the mixture was left to stir at 1400 rpm for 2h, in the dark. The reaction mixture was then concentrated *in vacuo*, diluted with 4 mL MQ (0.1% TFA) and spun down (4500 x *g*, 5 min, RT) and the supernatant was injected in the RP HPLC (2 runs). The pellet was then dissolved in 6 mL MQ:MeCN:TFA (79.9:20:0.1), vortexed (10min), and left in the sonicator (10 min, RT). The resulting solution was purified by RP HPLC and product fractions were concentrated *in vacuo* and lyophilized, yielding a dark blue fluffy powder (29.5 mg, 92.3%). HPLC: Rt. 17.509 min. HRMS (ESI+) m/z calcd for $C_{113}H_{164}N_{29}O_{27}S_3$ $[M+2H]^{2+}$ 1228.07959, found 1228.07731. $C_{113}H_{165}N_{29}O_{27}S_3$ $[M+3H]^{3+}$ 819.38995, found 819.38689.

CCP4(dimer)-NH$_2$ (10). An RND flask was charged with MQ (4 mL) and triazin **3** (1.60mg, 6,53 µmol, 1 eq) and THPTA (28.4 mg, 65.3 µmol, 10 eq). This solution was then bubbled through with argon for 30min. To the stirring solution under argon (at max speed) was then added CuSO4.5H2O (3.26 mg, 13.1 µmol, 2 eq) and once dissolved CCP4-N$_3$ **6** (25.0 mg, 13.1 µmol, 2 eq). Finally, sodium ascorbate (12.9 mg, 65.3 µmol, 10 eq) was added and the reaction was monitored using LC-MS or HPLC. Upon completion, the mixture was diluted to 5 mL MQ + 0.1% TFA and purified using RP HPLC to yield a fluffy white powder (11.5 mg, 2.82 µmol, 43.2%). HPLC: Rt. 15.904 min. HRMS (ESI+) m/z calcd for $C_{177}H_{273}N_{67}O_{42}S_2$ $[M+2H]^{2+}$ 2037.53886, found 2037.53517. $C_{177}H_{274}N_{67}O_{42}S_2$ $[M+3H]^{3+}$ 1358.69518, found 1358.69121. $C_{177}H_{275}N_{67}O_{42}S_2$ $[M+5H]^{5+}$ 1019.27334, found 1019.26812.

CCP4(dimer)-Biotin (11). A flask was charged with MQ and DMSO (4:1, 2.5 mL) and bubbled through with argon. Triazin **4** (1.61 mg, 3.41 µmol, 1.08 eq) and THPTA (13.8 mg, 31.8 µmol, 10 eq) were added to the flask. Subsequently, $CuSO_4.5H_2O$ (1.49 mg, 5.97 µmol, 1.89 eq) was added to the mixture. Once fully dissolved, CCP4-N$_3$ **6** (12.1 mg, 6.32 µmol, 2 eq) and sodium ascorbate (6.31 mg, 31.9 µmol, 10.1 eq) were added. The mixture was then diluted with MQ (+0.1% TFA, 2.5 mL) and the product was purified by RP HPLC. Product fractions were pooled, concentrated *in vacuo* and lyophilized to yield a white powder (8.12 mg, 1.89 µmol, 59.7%). HPLC: Rt. 16.415 min. LCMS (ESI+) m/z calcd for $C_{187}H_{288}N_{69}O_{44}S_3$ $[M+3H]^{3+}$ 1433.39, found 1434.48. $C_{187}H_{289}N_{69}O_{44}S_3$ $[M+4H]^{4+}$ 1075.29, found 1076.12. $C_{187}H_{290}N_{69}O_{44}S_3$ $[M+5H]^{5+}$ 860.43, found 861.12. $C_{187}H_{291}N_{69}O_{44}S_3$ $[M+6H]^{6+}$ 717.20, found 717.68. $C_{187}H_{292}N_{69}O_{44}S_3$ $[M+7H]^{7+}$ 614.88, found 615.44. $C_{187}H_{293}N_{69}O_{44}S_3$ $[M+8H]^{8+}$ 538.15, found 538.56.

CCP4(dimer)-AF594 (12). In a flame-dried round bottom flask with activated 3A mol sieves was added 1.9 mL anhydrous DMF and 110.2 µL Triazin **3** (10 mM in DMF, 0.27 mg, 1.1 µmol, 2 eq). DIPEA (0.96 µL, 0.71 mg, 5.5 µmol, 10 eq) was then added and the solution was left overnight under argon. The following day, AF594-NHS (0.90 mg, 1.1 µmol, 2 eq) was added to the reaction vessel. After 2 hours, the reaction mixture was concentrated *in vacuo* and resuspended in 2.0 mL DMF. A round bottom flask was filled with 1.0 mL of Triazin **3**-AF594 (1.0 mg, 1.1 µmol, 1 eq), 2 mL MQ and THPTA (4.6 mg, 11 µmol, 10 eq). This solution was then bubbled through with argon for 30min. To the stirring solution under argon (at max speed) was then added 21.2 µL $CuSO_4.5H_2O$ (100 mM, 0.53 mg, 2.1 µmol, 2 eq) and once dissolved CCP4-N_3 **6** (4.4 mg, 2.3 µmol, 2.2 eq). Finally, sodium ascorbate (2.1 mg, 11 µmol, 10 eq) was added and the reaction was monitored using LC-MS or HPLC. Upon completion, the mixture was diluted with 5 mL MQ + 0.1% TFA, sonicated and spun down at 4500 x g, 5min, RT. The supernatant was purified using RP-HPLC to yield a fluffy purple powder (1.23 mg, 0.257 µmol, 24%). HPLC: Rt. 17.450 min. HRMS (ESI+) m/z calcd for $C_{212}H_{305}N_{69}O_{52}S_4$ $[M+2H]^{2+}$ 2390.11474, found 2390.07373. $C_{212}H_{306}N_{69}O_{52}S_4$ $[M+3H]^{3+}$ 1593.74577, found 1593.74317. $C_{212}H_{307}N_{69}O_{52}S_4$ $[M+4H]^{4+}$ 1195.56128, found 1195.55163. $C_{212}H_{308}N_{69}O_{52}S_4$ $[M+5H]^{5+}$ 956.65059, found 956.64632.

CCP4(dimer)-SCy5 (13). To a flame dried flask under argon was added anhydrous DMSO (0.3 mL), **10** (4.40 mg, 1.08 µmol, 1 eq) and SulfoCy5-NHS (0.91 mg, 1.2 µmol, 1.1 eq). Subsequently anhydrous DIPEA (3.76 µL, 21.6 µmol, 20 eq) was added and the mixture was left to stir at 1400 rpm for 2 hours. The reaction mixture was then diluted with 2 mL MQ:ACN:TFA (8:2:0.1), centrifuged (4500 x *g*, 5 min, RT) and the supernatant was injected in the RP HPLC. Product fractions were concentrated *in vacuo* and lyophilized, yielding a blue fluffy powder (3.30 mg, 0.702 µmol, 65.0%). HPLC: Rt. 17.069 min. LCMS (ESI+) m/z calcd for $C_{209}H_{310}N_{69}O_{49}S_4$ $[M+3H]^{3+}$ 1566.09, found 1566.76. $C_{209}H_{311}N_{69}O_{49}S_4$ $[M+4H]^{4+}$ 1174.82, found 1175.60. $C_{209}H_{312}N_{69}O_{49}S_4$ $[M+5H]^{5+}$ 940.06, found 940.76. $C_{209}H_{313}N_{69}O_{49}S_4$ $[M+6H]^{6+}$ 783.55, found 784.08.

References

1. Kissel, T.; Ge, C. R.; Hafkenscheid, L.; Kwekkeboom, J. C.; Slot, L. M.; Cavallari, M.; He, Y. B.; Van Schie, K. A.; Vergroesen, R. D.; Kampstra, A. S. B.; Reijm, S.; Stoeken-Rijsbergen, G.; Koeleman, C.; Voortman, L. M.; Heitman, L. H.; Xu, B. Z.; Pruijn, G. J. M.; Wuhrer, M.; Rispens, T.; Huizinga, T. W. J.; Scherer, H. U.; Reth, M.; Holmdahl, R.; Toes, R. E. M., Surface Ig variable domain glycosylation affects autoantigen binding and acts as threshold for human autoreactive B cell activation. *Sci Adv* **2022,** *8* (6).
2. Patra, S.; Kozura, B.; Huang, A. Y.; Enciso, A. E.; Sun, X.; Hsieh, J. T.; Kao, C. L.; Chen, H. T.; Simanek, E. E., Dendrimers terminated with dichlorotriazine groups provide a route to compositional diversity. *Org Lett* **2013,** *15* (15), 3808-11.

4. NMR, HPLC and LCMS spectra

¹H NMR spectrum of 4,6-Dichloro-N-(prop-2-yn-1-yl)-1,3,5-triazin-2-amine (triazin 1).

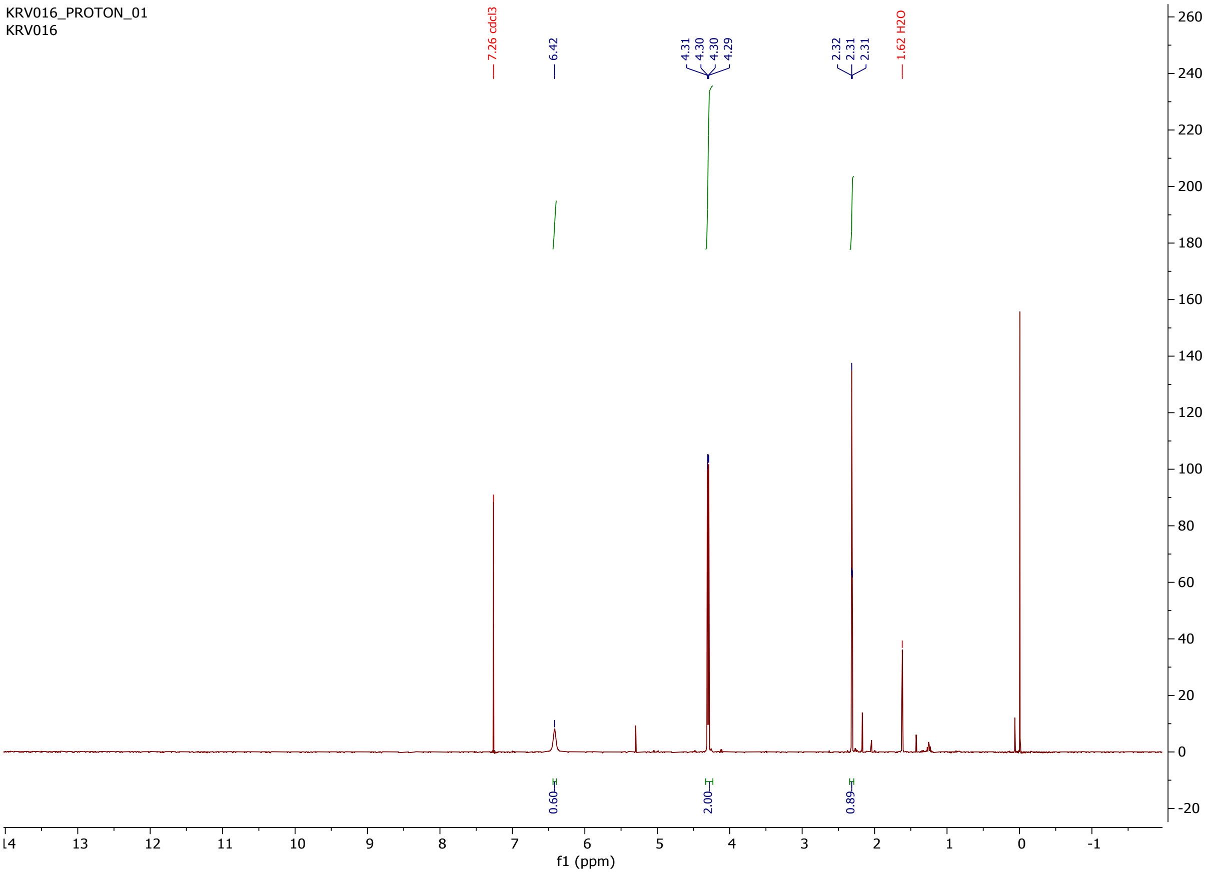

^{13}C NMR spectrum of 4,6-Dichloro-N-(prop-2-yn-1-yl)-1,3,5-triazin-2-amine (triazin 1).

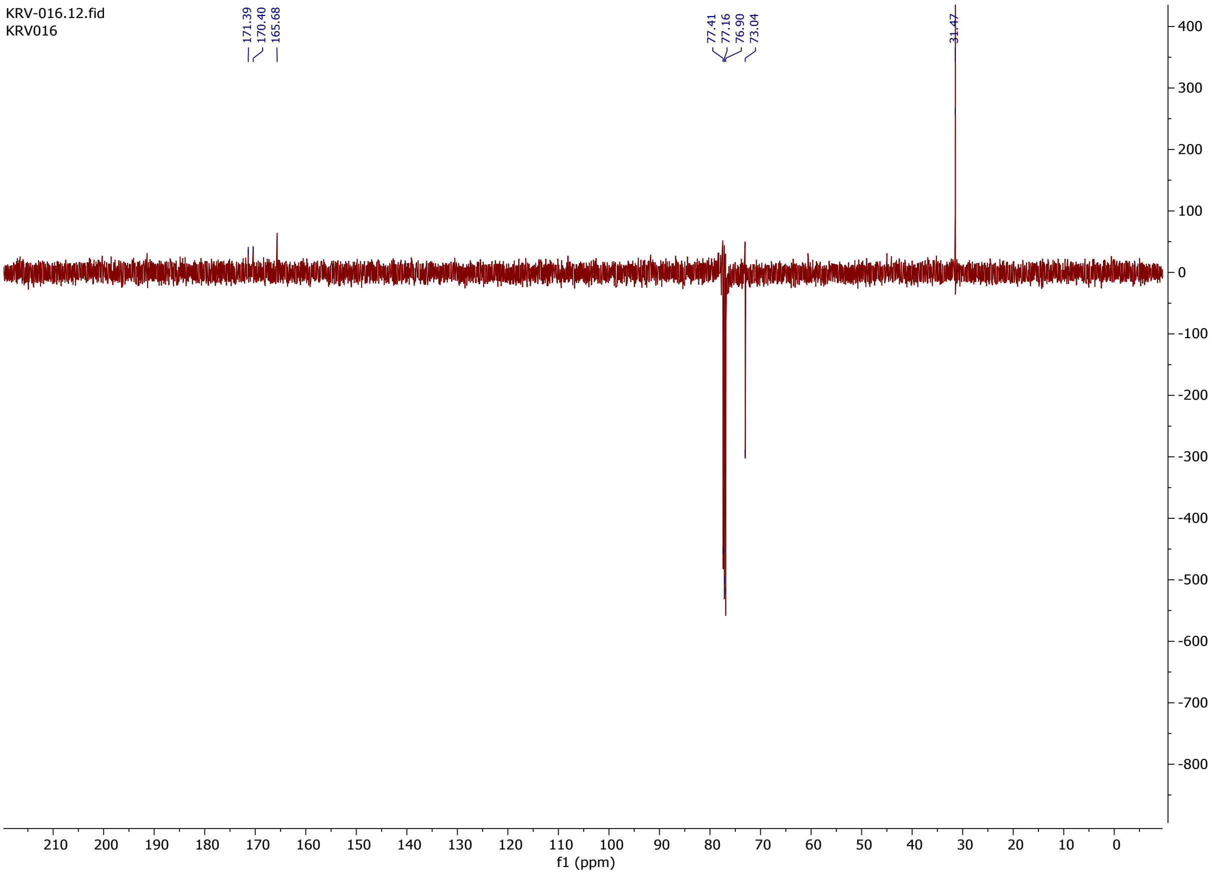

¹H NMR spectrum of Tert-butyl (2-((4-chloro-6-(prop-2-yn-1-ylamino)-1,3,5-triazin-2-yl)amino)ethyl)carbamate (2).

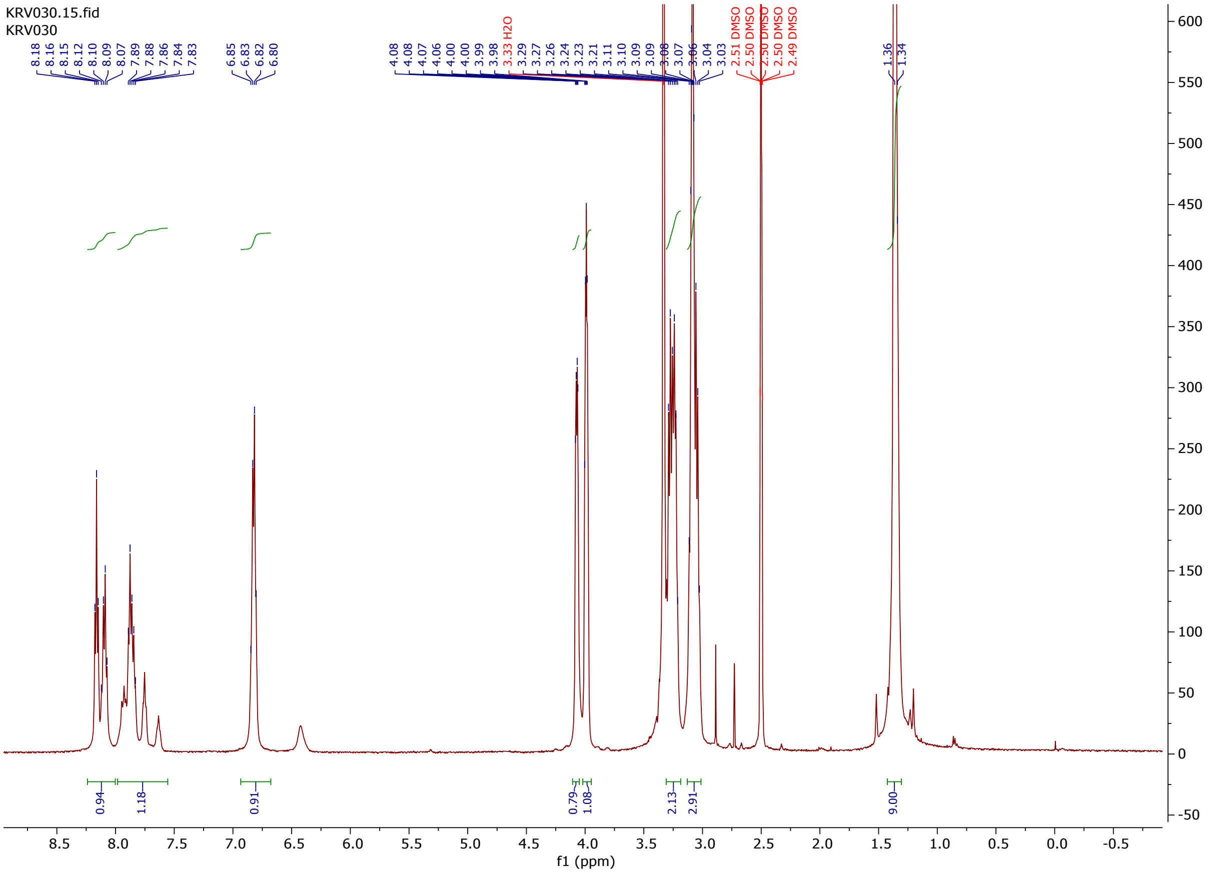

^{13}C NMR spectrum of Tert-butyl (2-((4-chloro-6-(prop-2-yn-1-ylamino)-1,3,5-triazin-2-yl)amino)ethyl)carbamate (2).

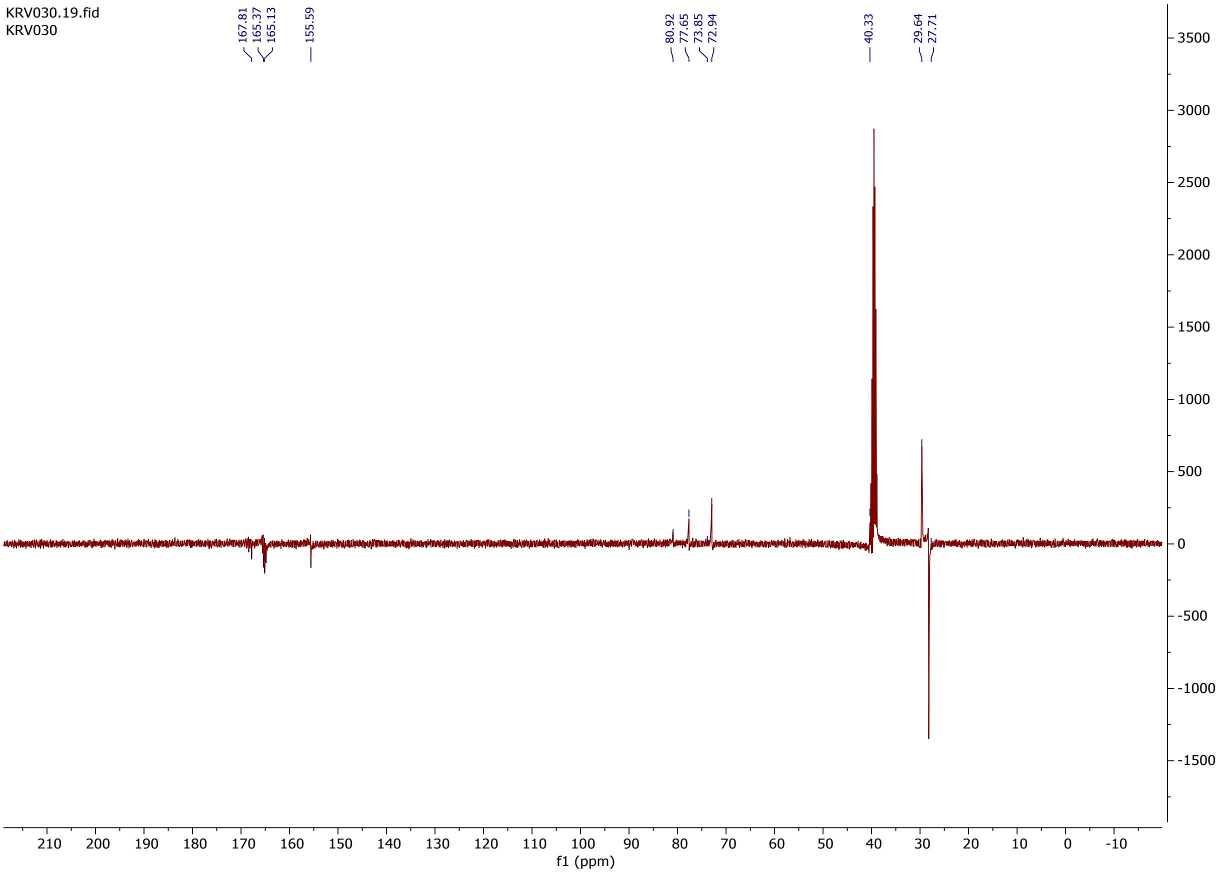

HPLC spectrum of Tert-butyl (2-((4-chloro-6-(prop-2-yn-1-ylamino)-1,3,5-triazin-2-yl)amino)ethyl)carbamate (2).

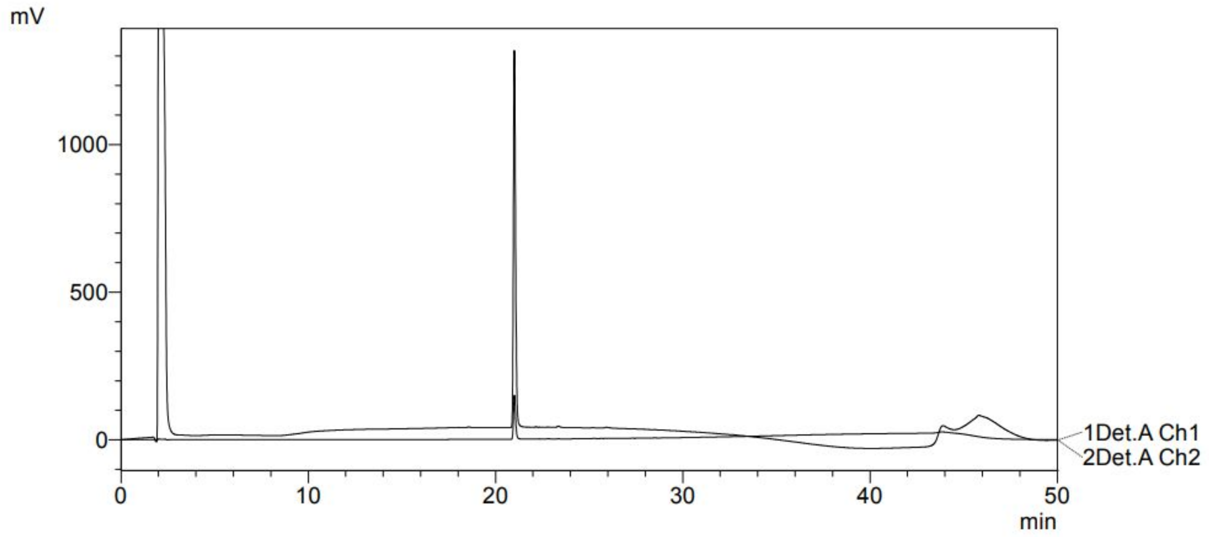

LRMS spectrum of Tert-butyl (2-((4-chloro-6-(prop-2-yn-1-ylamino)-1,3,5-triazin-2-yl)amino)ethyl)carbamate (2).

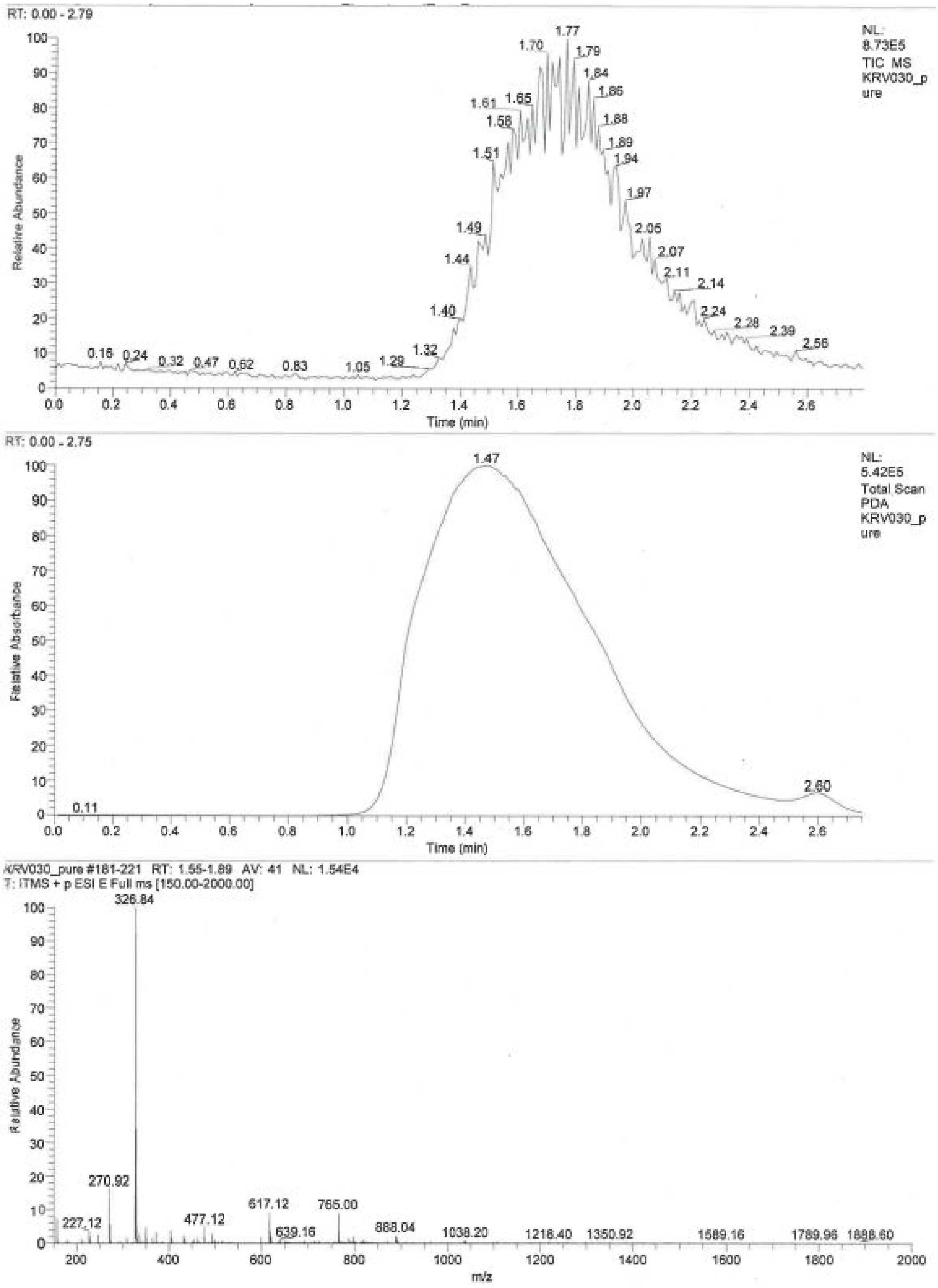

^{1}H NMR spectrum of N2-(2-aminoethyl)-N4,N6-di(prop-2-yn-1-yl)-1,3,5-triazine-2,4,6-triamine (3).

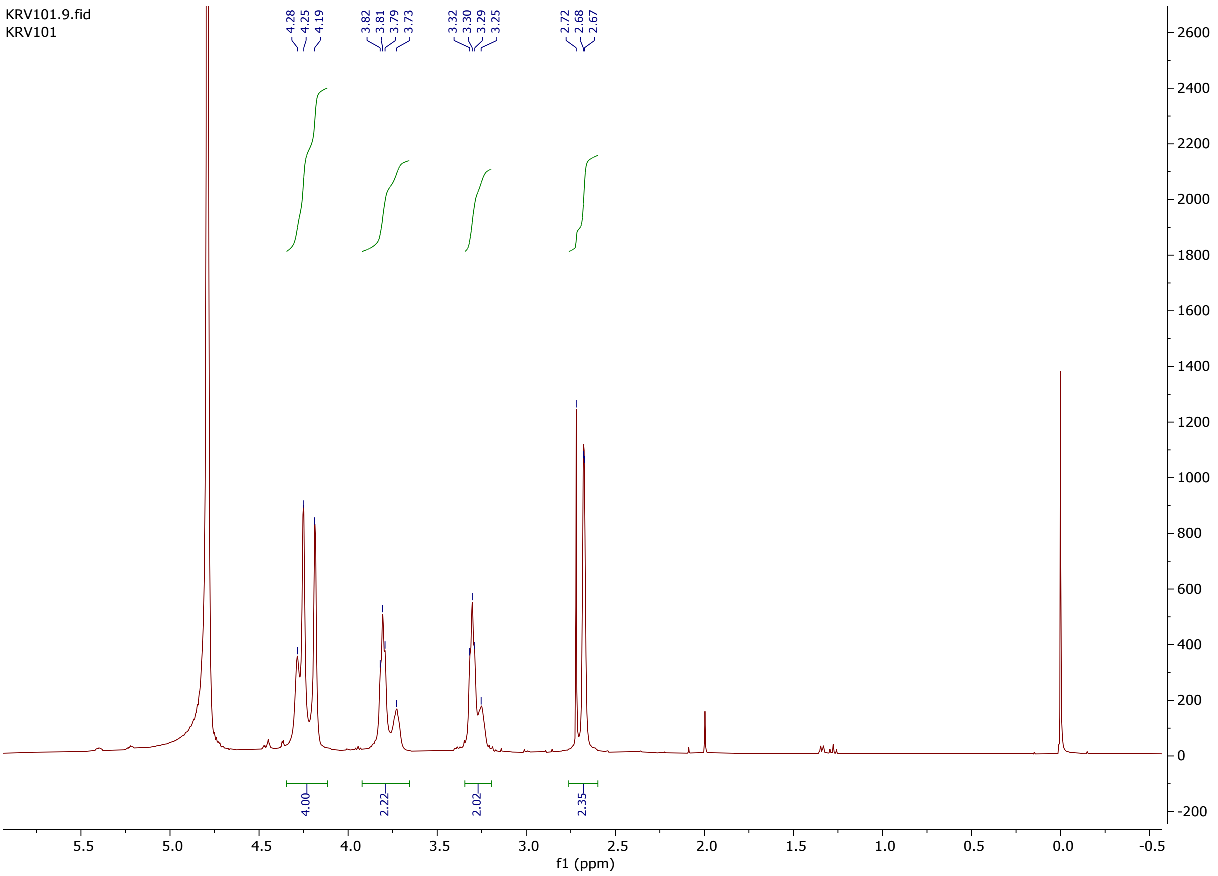

^{13}C NMR spectrum of N2-(2-aminoethyl)-N4,N6-di(prop-2-yn-1-yl)-1,3,5-triazine-2,4,6-triamine (3).

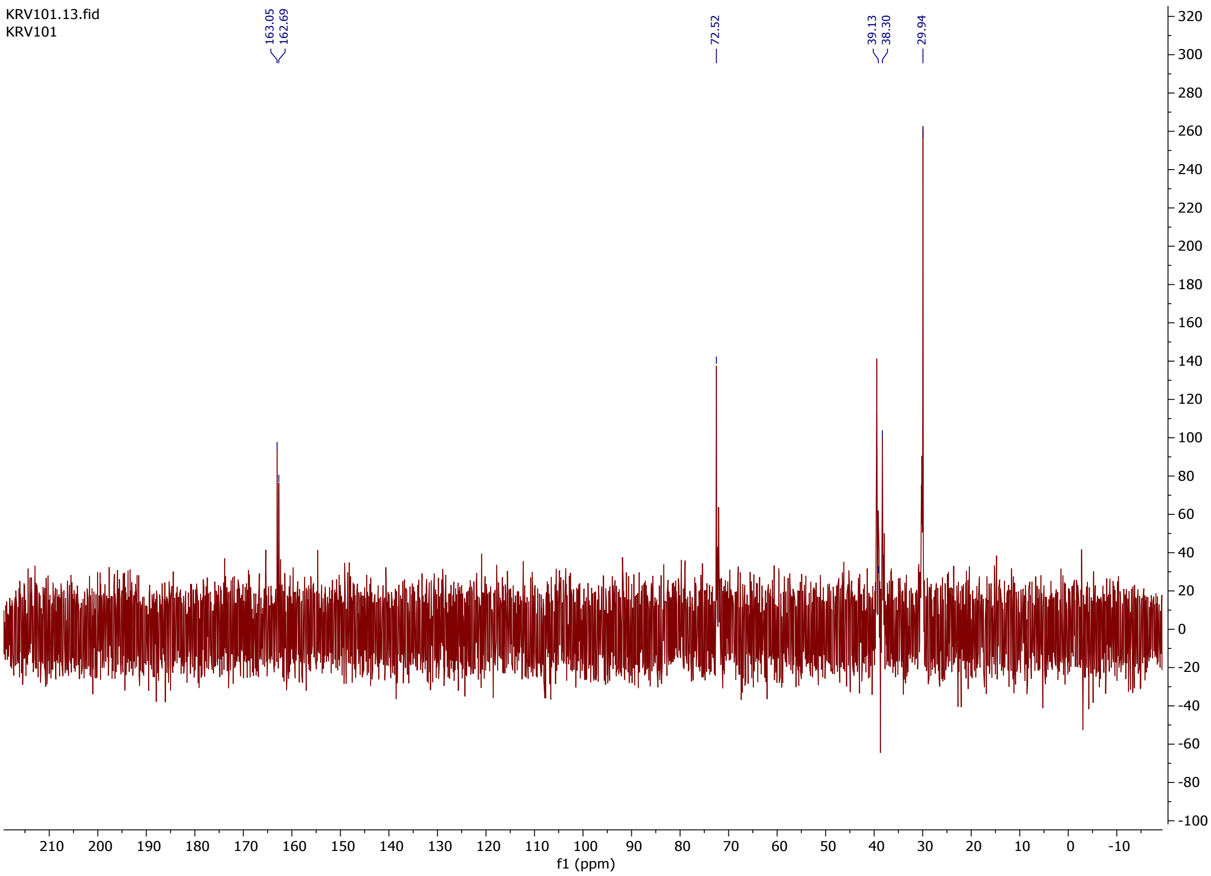

HPLC spectrum of N2-(2-aminoethyl)-N4,N6-di(prop-2-yn-1-yl)-1,3,5-triazine-2,4,6-triamine (3).

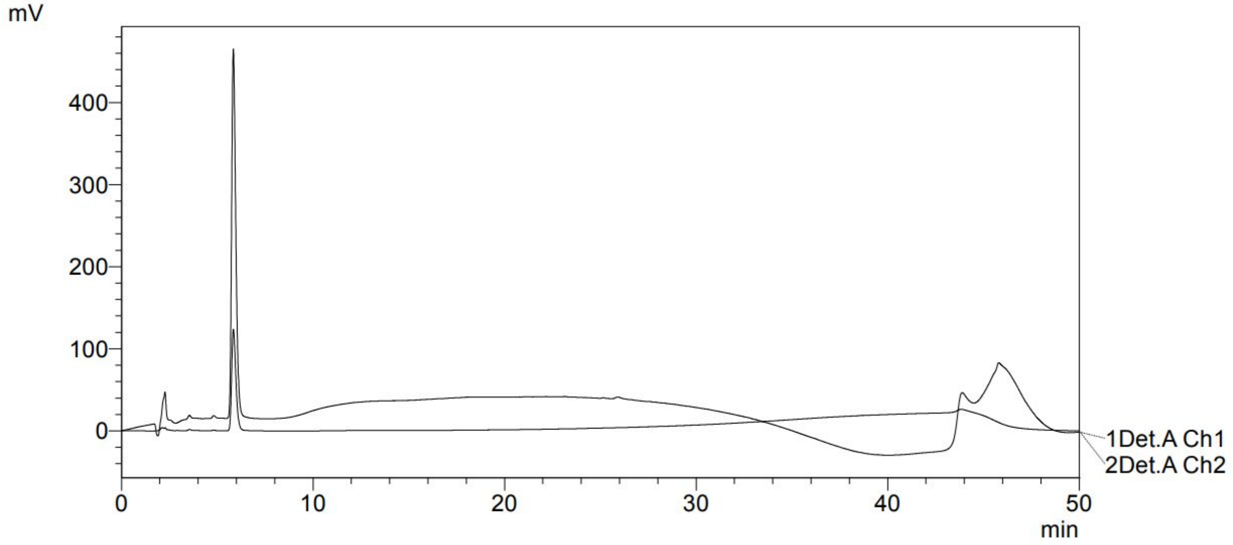

LRMS spectrum of N2-(2-aminoethyl)-N4,N6-di(prop-2-yn-1-yl)-1,3,5-triazine-2,4,6-triamine (3).

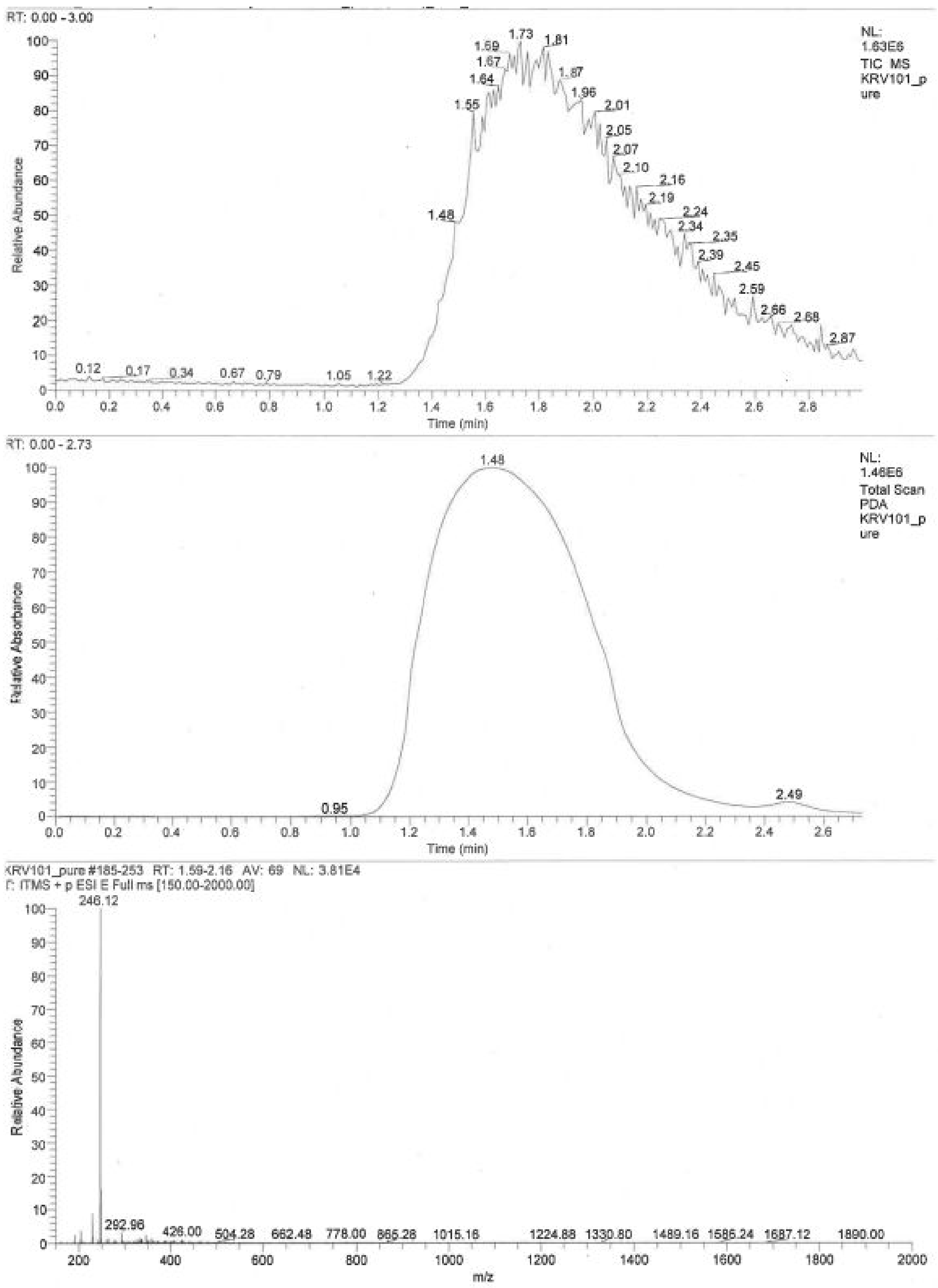

LC-MS analysis of N2-(2-aminoethyl)-N4,N6-di(prop-2-yn-1-yl)-1,3,5-triazine-2,4,6-triamine (4).

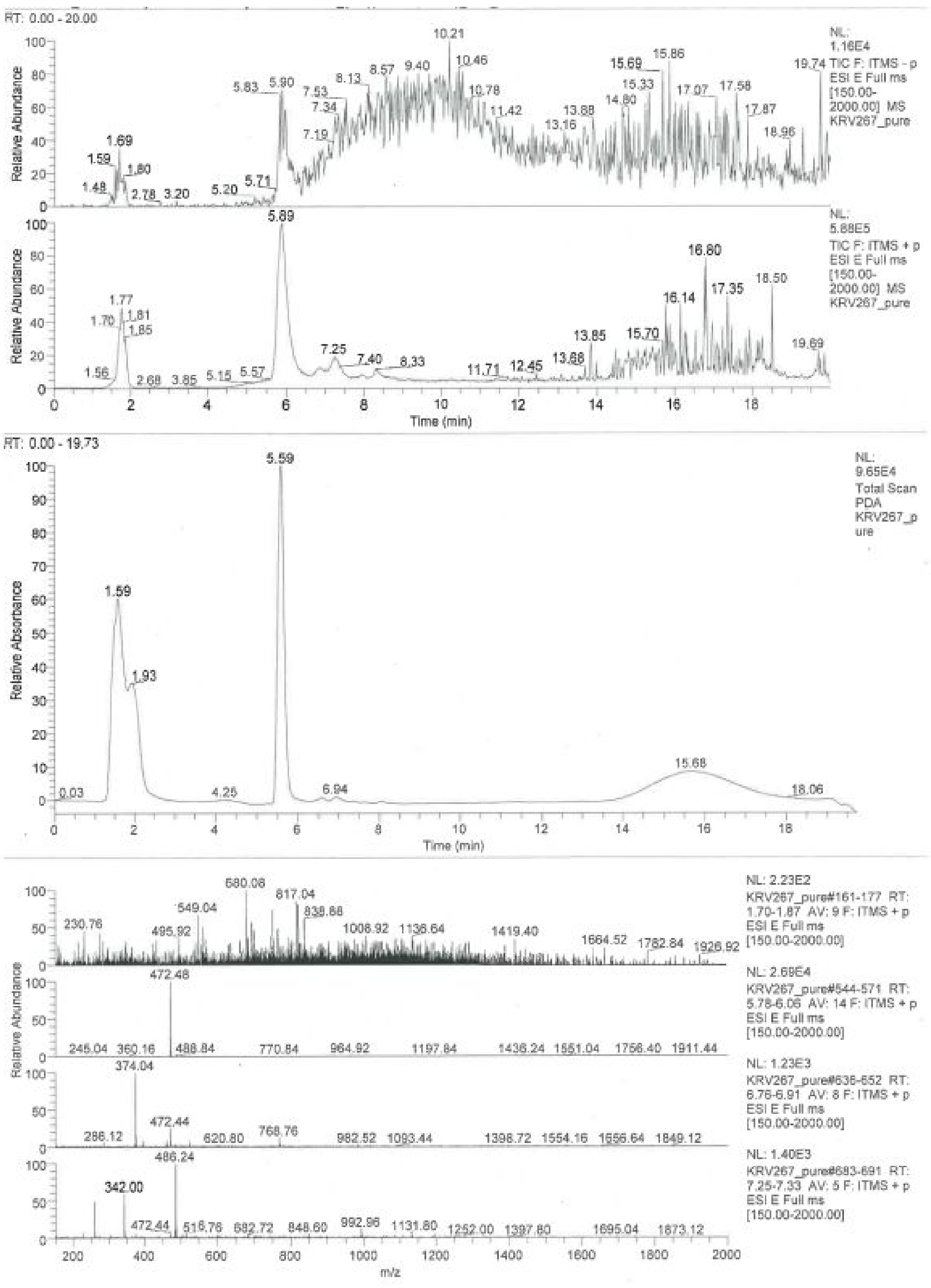

Analytical HPLC spectrum of Cyclic Citrullinated Peptide 4 (5).

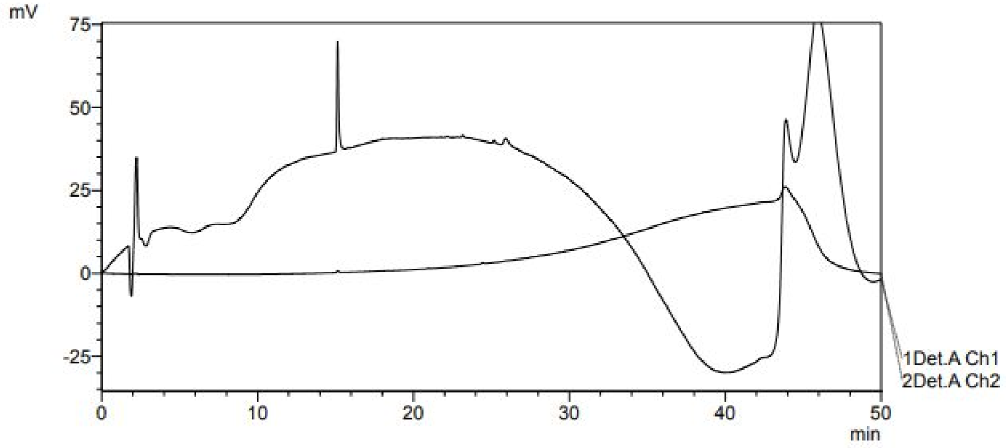
mV
75
50
25
0
-25
0
10
20
30
40
50
min
1Det.A Ch1
2Det.A Ch2

High resolution MS spectrum of Cyclic Citrullinated Peptide 4 (5).

Charge number:1 Tolerance:3.00(ppm), 3.00 .. 10.00(mmu) Unsaturation Number:-1.5 .. 999.0 (Fraction:.5)

Element:^{12}C:81 .. 81, ^{1}H:1 .. 200, ^{14}N:10 .. 30, ^{23}Na:0 .. 2, ^{16}O:10 .. 30, ^{32}S:1 .. 5

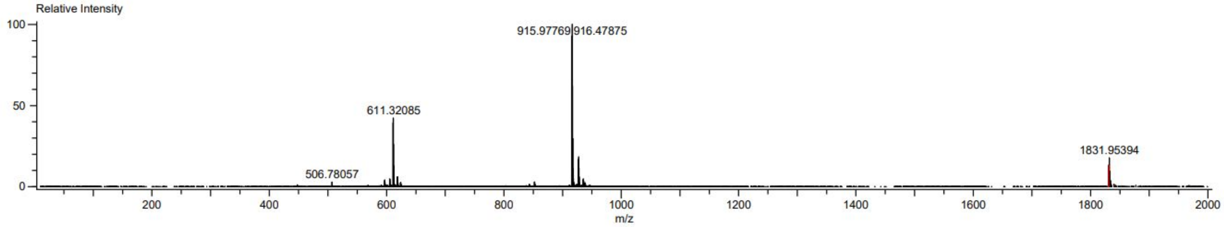

Mass	Decision	Calc. Mass	Mass Difference (mmu)	Mass Difference (ppm)	^{12}C	^{1}H	^{14}N	^{23}Na	^{16}O	^{32}S	Unsaturation Number
1830.95469	+	1830.95496	-0.27	-0.15	81	128	27		20	1	32.5
	?	1830.95476	-0.07	-0.04	81	5	28	2	24	1	93.5
	?	1830.95449	0.20	0.11	81	136	23		19	3	28.5
	?	1830.95429	0.40	0.22	81	13	24	2	23	3	89.5
	?	1830.95429	0.40	0.22	81	137	27	1	10	5	31.5
	?	1830.95402	0.67	0.36	81	144	19		18	5	24.5
	?	1830.95382	0.87	0.48	81	21	20	2	22	5	85.5
	?	1830.95355	1.14	0.62	81	28	12	1	30	5	78.5
	?	1830.95594	-1.25	-0.69	81	15	26		20	5	92.5
	?	1830.95641	-1.72	-0.94	81	7	30		21	3	96.5
	?	1830.95284	1.85	1.01	81	134	21	2	22	1	25.5
	?	1830.95257	2.12	1.16	81	141	13	1	30	1	18.5
	?	1830.95237	2.32	1.27	81	142	17	2	21	3	21.5
	?	1830.95190	2.79	1.53	81	150	13	2	20	5	17.5
	?	1830.95755	-2.86	-1.56	81	146	11	2	28	2	15.5
	?	1830.95901	-4.32	-2.36	81	25	14	2	29	4	79.5
	?	1830.95921	-4.52	-2.47	81	148	13		25	4	18.5
	?	1830.95948	-4.79	-2.61	81	17	18	2	30	2	83.5
	?	1830.95948	-4.79	-2.62	81	141	21	1	17	4	25.5
	?	1830.95968	-4.99	-2.72	81	140	17		26	2	22.5
	?	1830.95995	-5.26	-2.87	81	133	25	1	18	2	29.5
	?	1830.94930	5.39	2.94	81	8	26	1	25	2	92.5
	?	1830.94930	5.39	2.94	81	132	29		12	4	34.5

LC-MS analysis of Cyclic Citrullinated Peptide 4 (5).

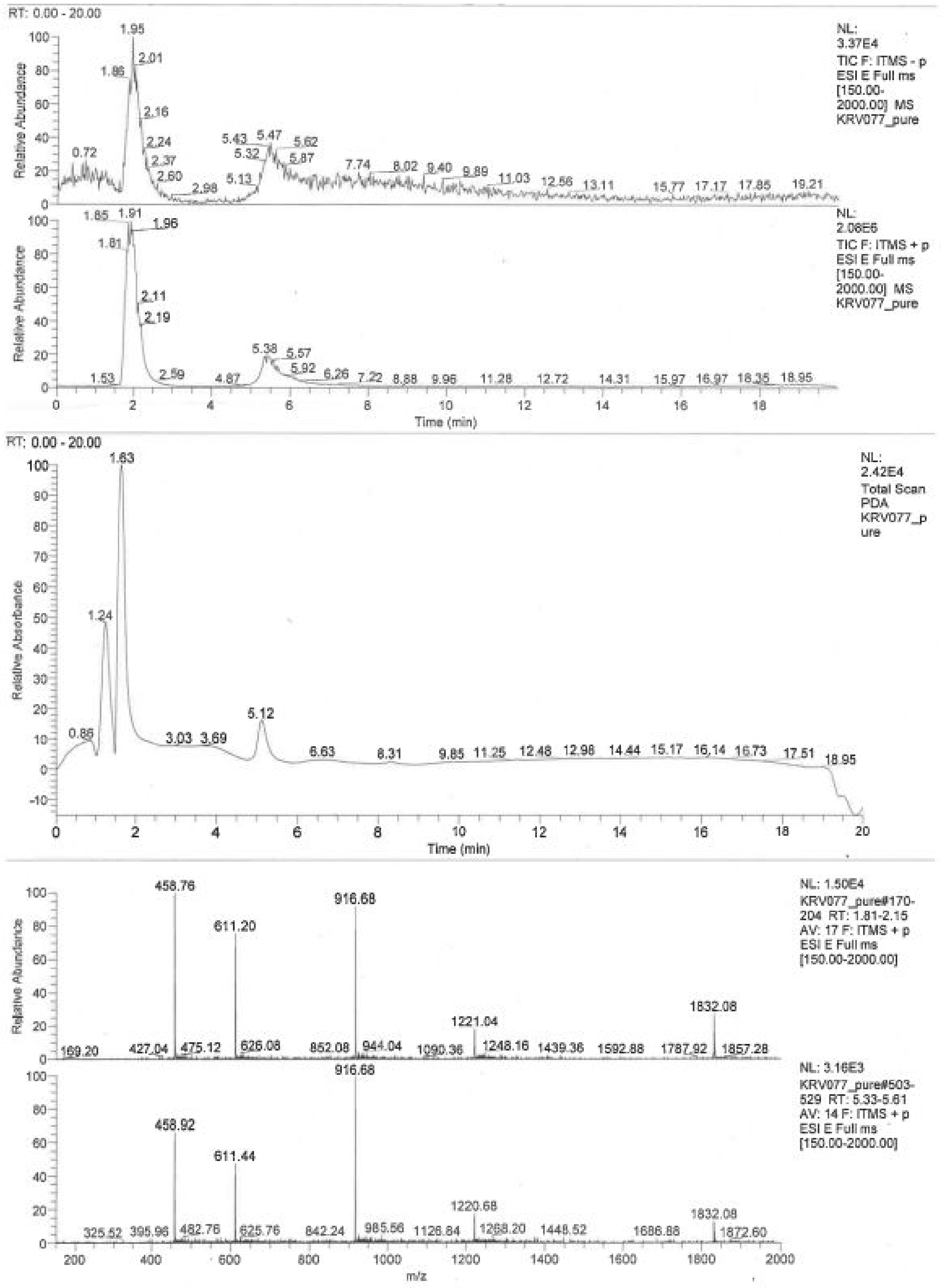

HPLC spectrum of CCP4-N_3 (6).

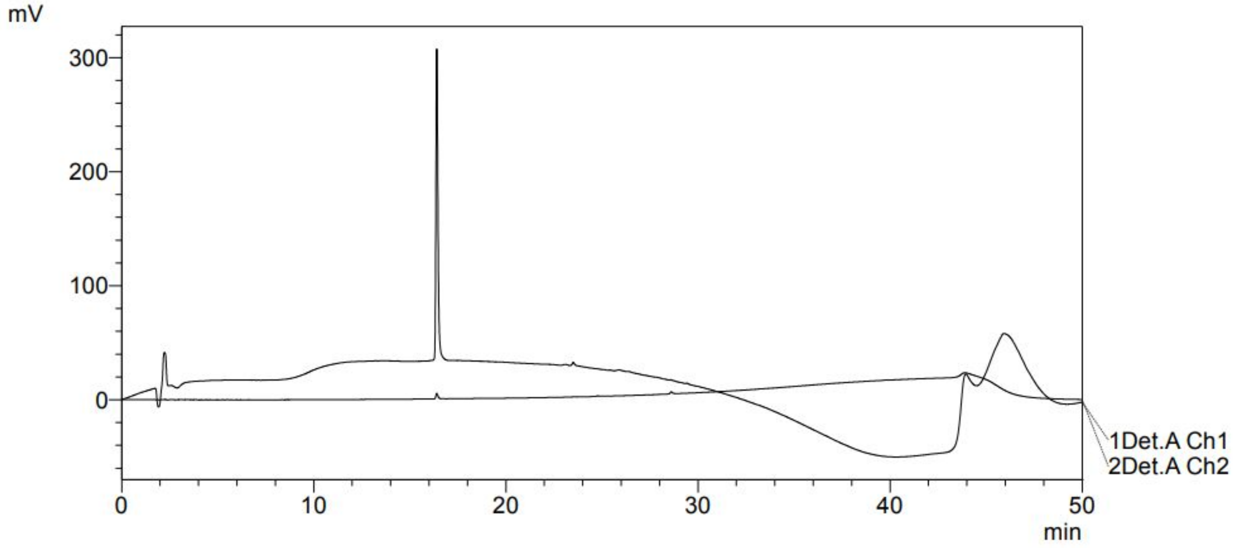

High resolution MS spectrum of CCP4-N_3 (6).

Data:KRV322_pure
Sample Name:Kevin Venrooij [Bon], M=1912
Description:
Ionization Mode:ESI+
History:Determine m/z[Peak Detect[Centroid,30,Area];Correct Base[]];Average(MS[1] 0.21..0.23)-1.0*...

Acquired:06-Feb-23 09:17:44
Operator:Accutof
Mass Calibration data:TFANa_ESI+_2000
Created:06-Feb-23 09:38:49
Created by:Accutof

Charge number:1
Tolerance:3.00(ppm), 3.00 .. 10.00(mmu)
Unsaturation Number:-1.5 .. 999.0 (Fraction:.5)
Element:^{12}C:83 .. 83, ^{1}H:1 .. 200, ^{14}N:10 .. 30, ^{23}Na:0 .. 2, ^{16}O:10 .. 30, ^{32}S:1 .. 5

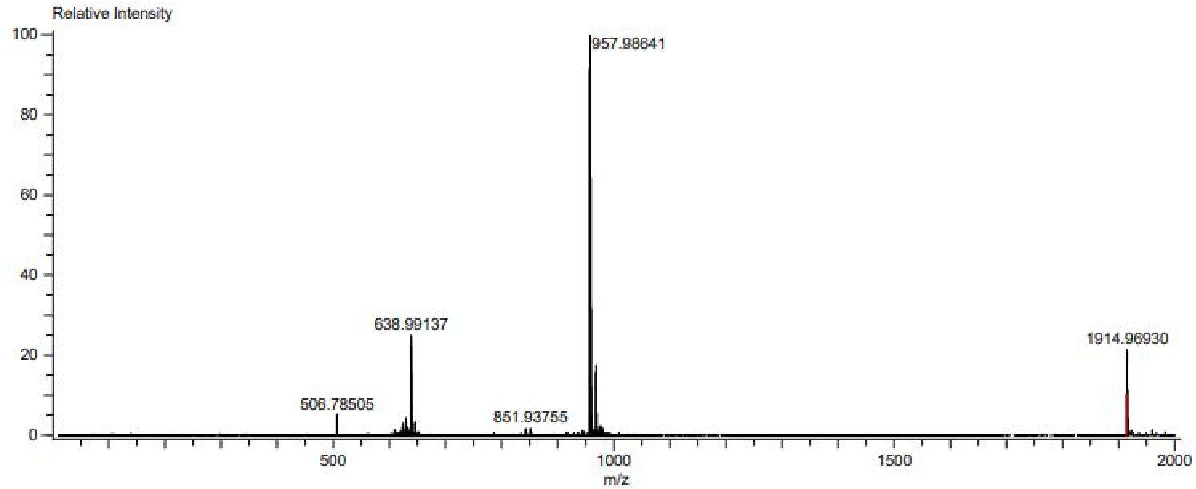

Mass	Decision	Calc. Mass	Mass Difference (mmu)	Mass Difference (ppm)	^{12}C	^{1}H	^{14}N	^{23}Na	^{16}O	^{32}S	Unsaturation Number
1913.97036	+	1913.96692	3.44	1.80	83	129	30		21	1	35.5
	?	1913.97097	-0.61	-0.32	83	26	17	2	30	4	82.5
	?	1913.97117	-0.81	-0.42	83	149	16		26	4	21.5
	?	1913.96952	0.84	0.44	83	147	14	2	29	2	18.5
	?	1913.97144	-1.08	-0.57	83	142	24	1	18	4	28.5
	?	1913.97164	-1.28	-0.67	83	141	20		27	2	25.5
	?	1913.96905	1.31	0.69	83	155	10	2	28	4	14.5
	?	1913.97191	-1.55	-0.81	83	134	28	1	19	2	32.5
	?	1913.96791	2.45	1.28	83	16	29		21	5	95.5
	?	1913.97309	-2.74	-1.43	83	20	23		28	4	89.5
	?	1913.97356	-3.20	-1.67	83	12	27		29	2	93.5
	?	1913.96645	3.90	2.04	83	137	26		20	3	31.5
	?	1913.96625	4.10	2.14	83	138	30	1	11	5	34.5
	?	1913.96625	4.11	2.15	83	14	27	2	24	3	92.5
	?	1913.96598	4.37	2.29	83	145	22		19	5	27.5
	?	1913.96578	4.58	2.39	83	22	23	2	23	5	88.5
	?	1913.96480	5.56	2.91	83	135	24	2	23	1	28.5

LC-MS analysis of spectrum of CCP4-N_3 (6).

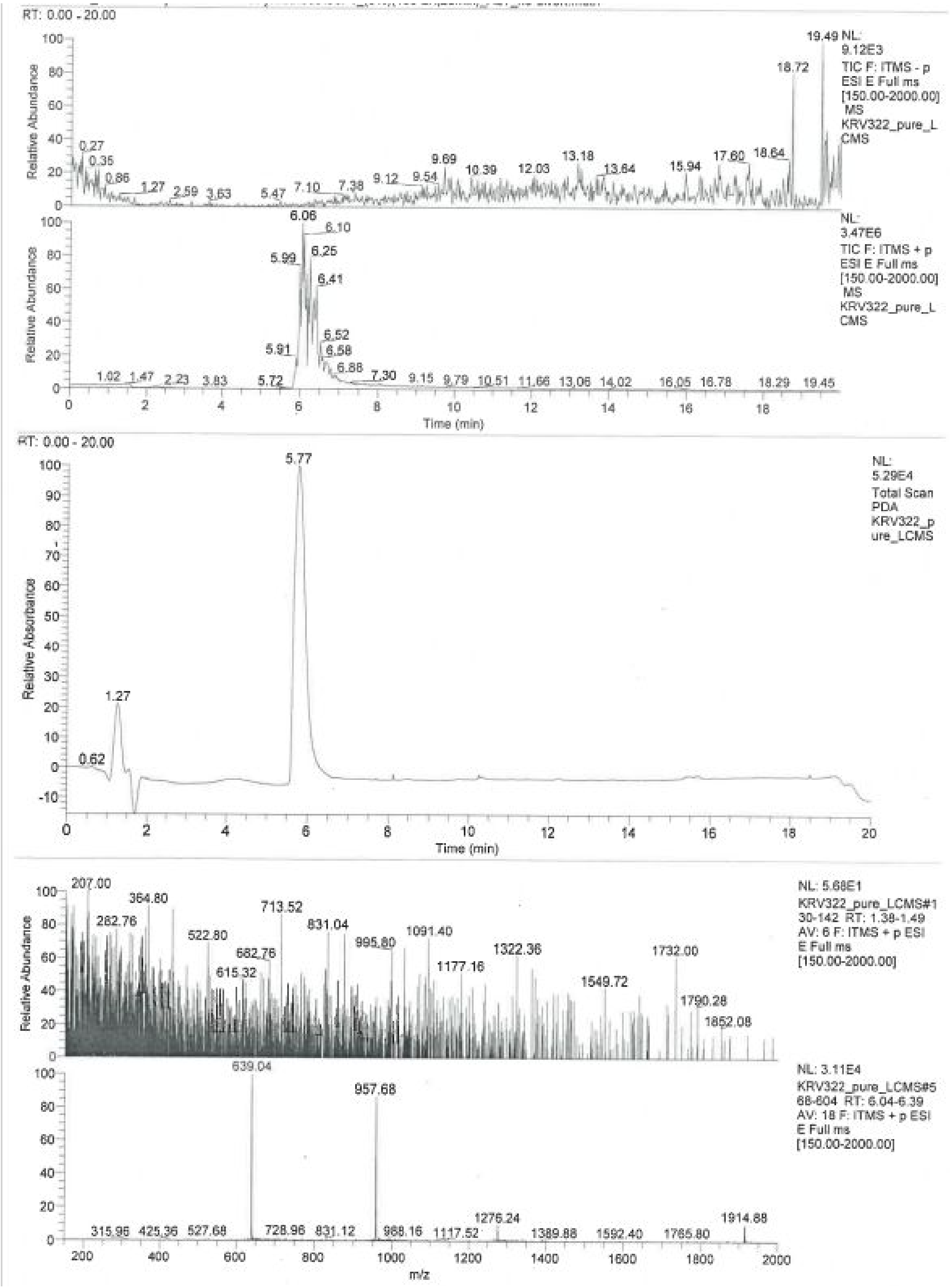

HPLC spectrum of CCP4-Biotin (7).

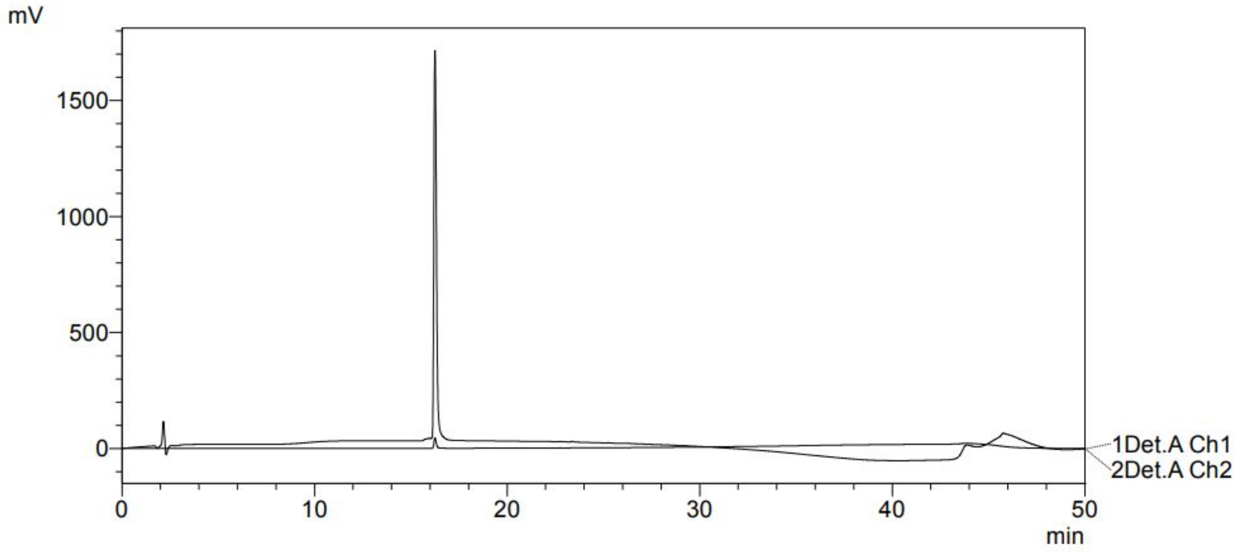

High resolution MS spectrum of CCP4-Biotin (7).

Data:KRV264_pure
Sample Name:Kevin Venrooij [Bon], M=2056
Description:
Ionization Mode:ESI+
History:Determine m/z[Peak Detect[Centroid,30,Area];Correct Base[]];Average(MS[1] 0.21..0.25)-1.0*...

Acquired:06-Feb-23 10:01:26
Operator:Accutof
Mass Calibration data:TFANa_ESI+_4000
Created:06-Feb-23 11:30:58
Created by:Accutof

Charge number:1
Tolerance:3.00(ppm), 3.00 .. 10.00(mmu)
Unsaturation Number:-1.5 .. 999.0 (Fraction:Both)
Element:^{12}C:91 .. 91, ^{1}H:100 .. 250, ^{14}N:20 .. 40, ^{23}Na:0 .. 2, ^{16}O:20 .. 40, ^{32}S:1 .. 5

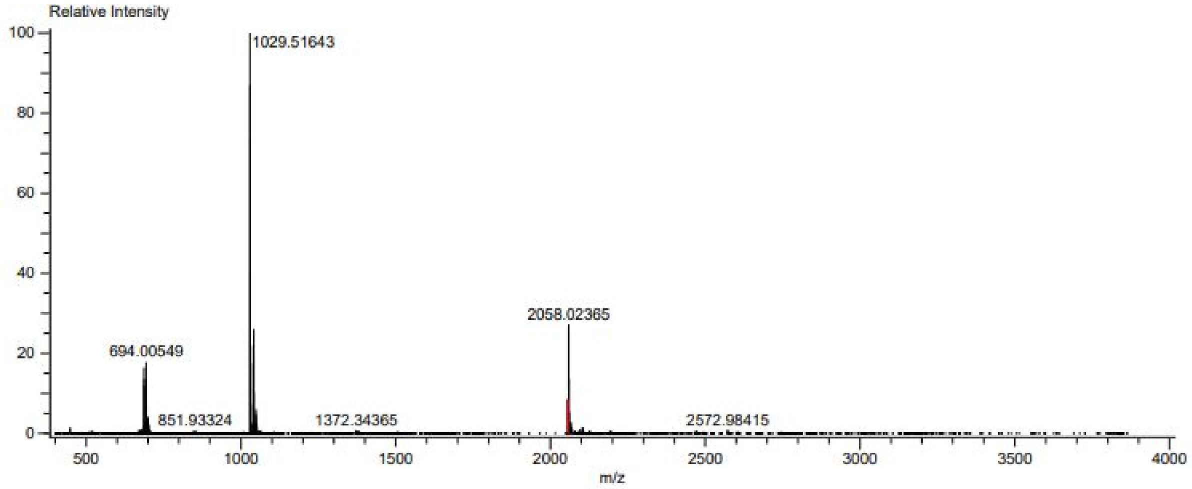

Mass	Decision	Calc. Mass	Mass Difference (mmu)	Mass Difference (ppm)	^{12}C	^{1}H	^{14}N	^{23}Na	^{16}O	^{32}S	Unsaturation Number
2057.02729	+	2057.03256	-5.27	-2.56	91	142	29		22	2	37.5
	?	2057.02651	0.78	0.38	91	140	28		25	1	37.0
	?	2057.02604	1.25	0.61	91	148	24		24	3	33.0
	?	2057.02557	1.72	0.84	91	156	20		23	5	29.0
	?	2057.02545	1.85	0.90	91	143	25	1	26	1	33.5
	?	2057.02498	2.32	1.13	91	151	21	1	25	3	29.5
	?	2057.02438	2.91	1.41	91	146	22	2	27	1	30.0
	?	2057.03043	-3.14	-1.53	91	148	23	2	24	2	30.5
	?	2057.03103	-3.74	-1.82	91	153	22	1	22	4	30.0
	?	2057.03150	-4.20	-2.04	91	145	26	1	23	2	34.0
	?	2057.03209	-4.80	-2.33	91	150	25		21	4	33.5

LC-MS analysis of CCP4-Biotin (7).

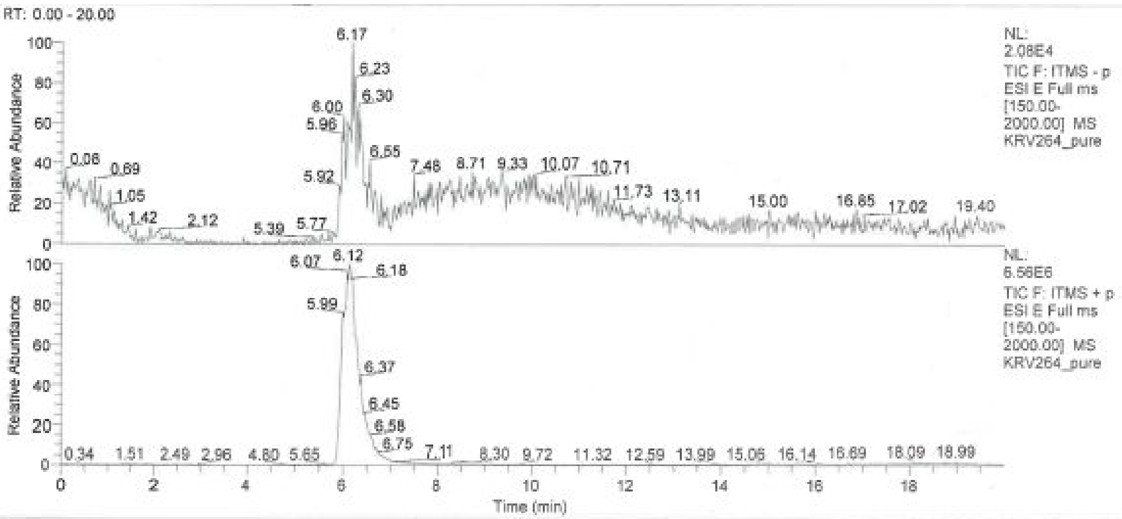
RT: 0.00 - 20.00
NL: 2.08E4
TIC F: ITMS - p ESI E Full ms [150.00-2000.00] MS KRV264_pure
NL: 6.56E6
TIC F: ITMS + p ESI E Full ms [150.00-2000.00] MS KRV264_pure
Relative Abundance
Time (min)
6.17
6.12

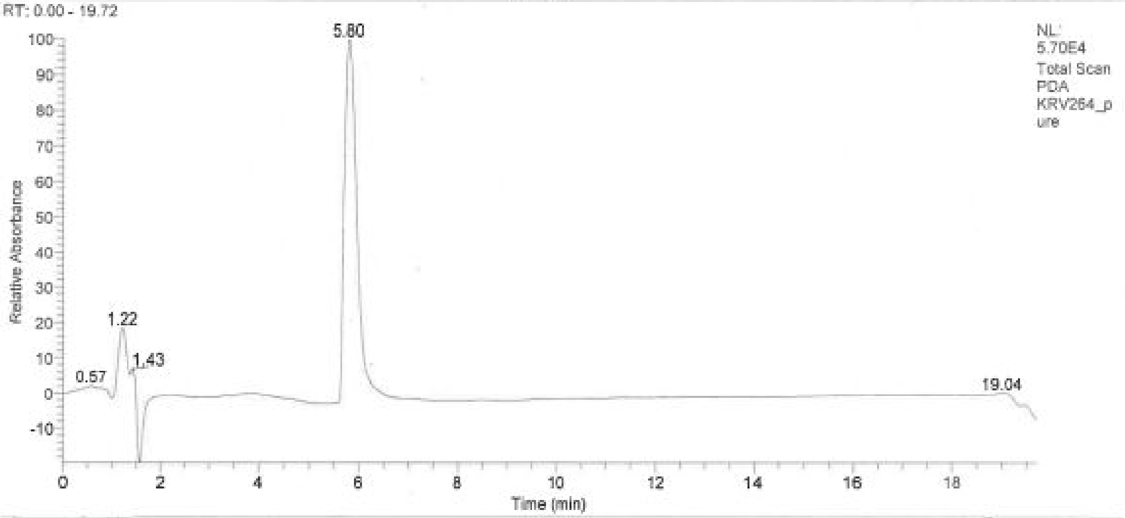
RT: 0.00 - 19.72
NL: 5.70E4
Total Scan PDA KRV264_pure
Relative Absorbance
Time (min)
5.80
1.22
1.43
0.57
19.04

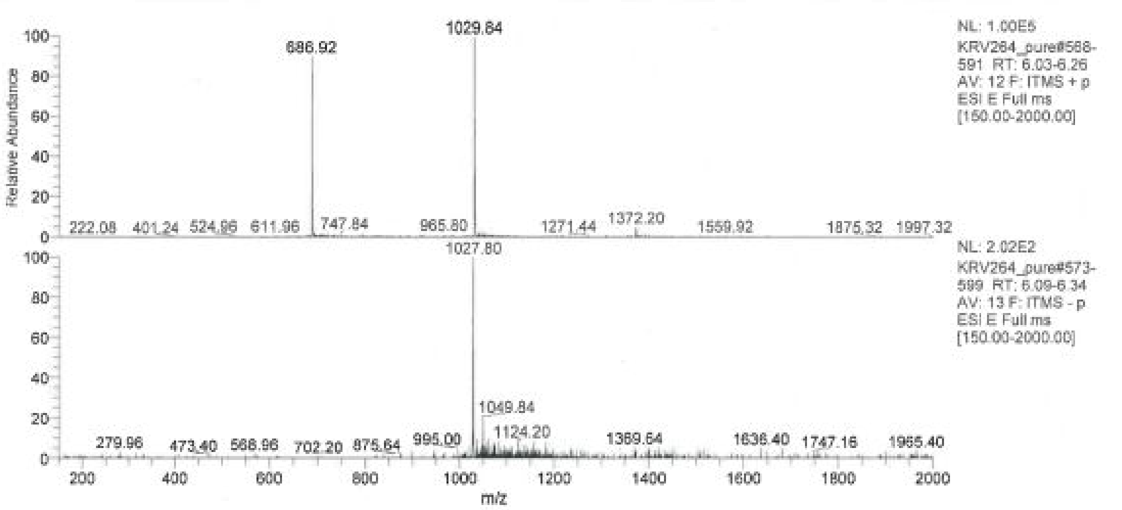
NL: 1.00E5
KRV264_pure#568-591 RT: 6.03-6.26 AV: 12 F: ITMS + p ESI E Full ms [150.00-2000.00]
NL: 2.02E2
KRV264_pure#573-599 RT: 6.09-6.34 AV: 13 F: ITMS - p ESI E Full ms [150.00-2000.00]
Relative Abundance
m/z
1029.84
686.92
1027.80
1049.84

HPLC spectrum of CCP4-AF594 (8).

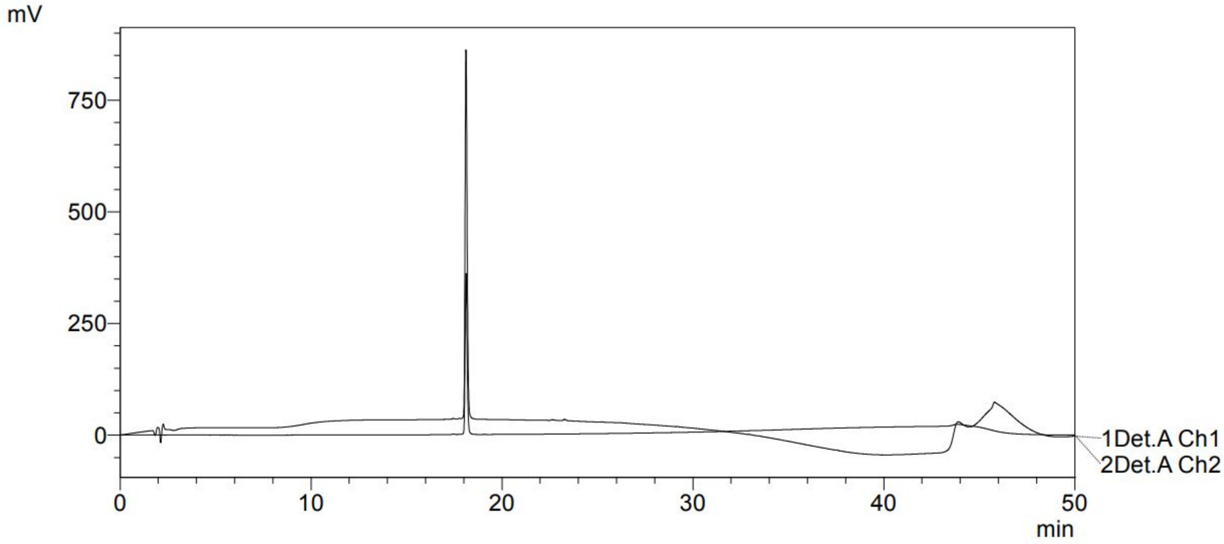

High resolution MS spectrum of CCP4-AF594 (8).

Data:KRV259
Sample Name:Kevin Venrooij [Bo], M=2534
Description:
Ionization Mode:ESI+
History:Determine m/z[Peak Detect[Centroid,30,Area];Correct Base[]];Average(MS[1] 0.21..0.26)-1.0*Average(MS[1] 0.07..0.11);Correct Base[5....

Acquired:28-Feb-22 09:43:50
Operator:Accutof
Mass Calibration data:TFANa_ESI+_2000
Created:28-Feb-22 10:59:41
Created by:Accutof

Charge number:2
Tolerance:5.00(ppm), 3.00 .. (mmu)
Unsaturation Number:-1.5 .. 999.0 (Fraction:Both)
Element:^{12}C:116 .. 116, ^{1}H:100 .. 200, ^{14}N:20 .. 40, ^{23}Na:2 .. 2, ^{16}O:20 .. 40, ^{32}S:1 .. 5

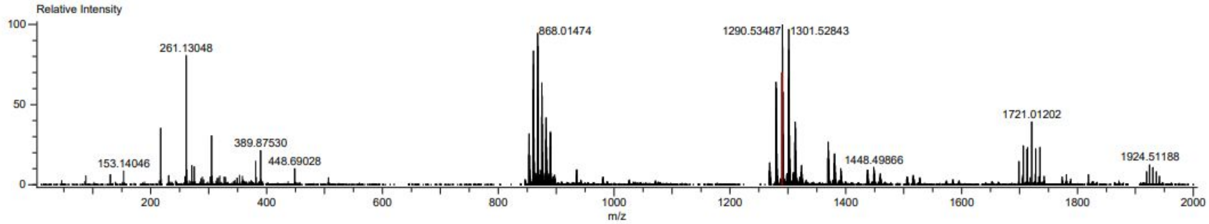

Mass	Decision	Calc. Mass	Mass Difference (mmu)	Mass Difference (ppm)	^{12}C	^{1}H	^{14}N	^{23}Na	^{16}O	^{32}S	Unsaturation Number
2580.07838	+	2580.07651	1.87	0.73	116	159	29	2	30	3	54.0
	?	2580.07784	0.54	0.21	116	149	40	2	21	3	64.5
	?	2580.07698	1.40	0.54	116	151	33	2	31	1	58.0
	?	2580.08303	-4.65	-1.80	116	153	34	2	28	2	58.5
	?	2580.07737	1.01	0.39	116	157	36	2	20	5	60.5
	?	2580.07565	2.73	1.06	116	161	22	2	40	1	47.5
	?	2580.08256	-4.18	-1.62	116	161	30	2	27	4	54.5
	?	2580.08170	-3.32	-1.29	116	163	23	2	37	2	48.0
	?	2580.07604	2.34	0.91	116	167	25	2	29	5	50.0

LC-MS analysis of CCP4-AF594 (8).

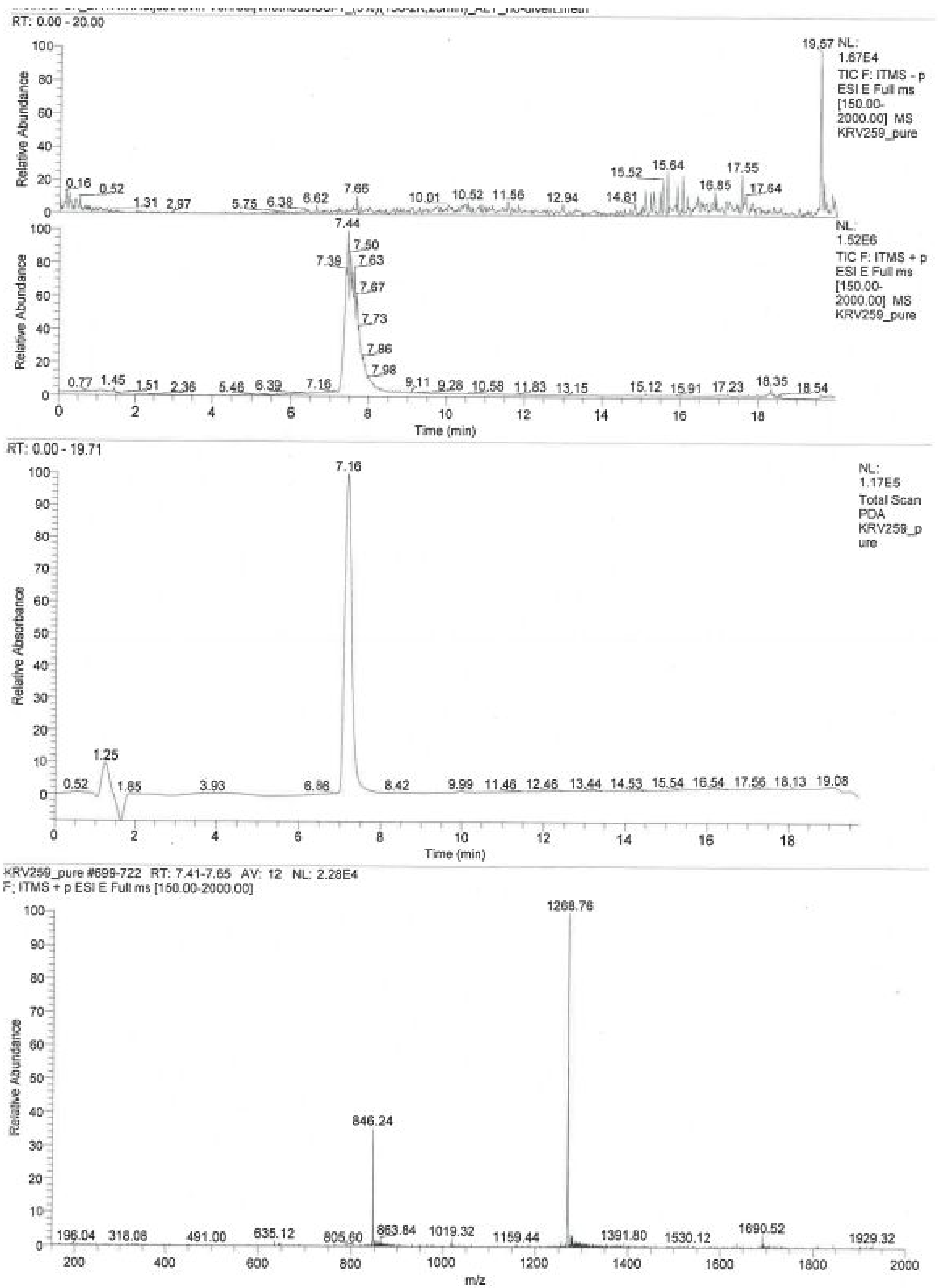
RT: 0.00 - 20.00
Relative Abundance
NL: 1.67E4
TIC F: ITMS - p ESI E Full ms [150.00-2000.00] MS KRV259_pure
19.57
NL: 1.52E6
TIC F: ITMS + p ESI E Full ms [150.00-2000.00] MS KRV259_pure
7.44
Time (min)
RT: 0.00 - 19.71
Relative Absorbance
7.16
NL: 1.17E5
Total Scan PDA KRV259_pure
1.25
Time (min)
KRV259_pure #699-722 RT: 7.41-7.65 AV: 12 NL: 2.28E4
F: ITMS + p ESI E Full ms [150.00-2000.00]
Relative Abundance
1268.76
846.24
1690.52
m/z

HPLC spectrum of CCP4-SCy5 (9).

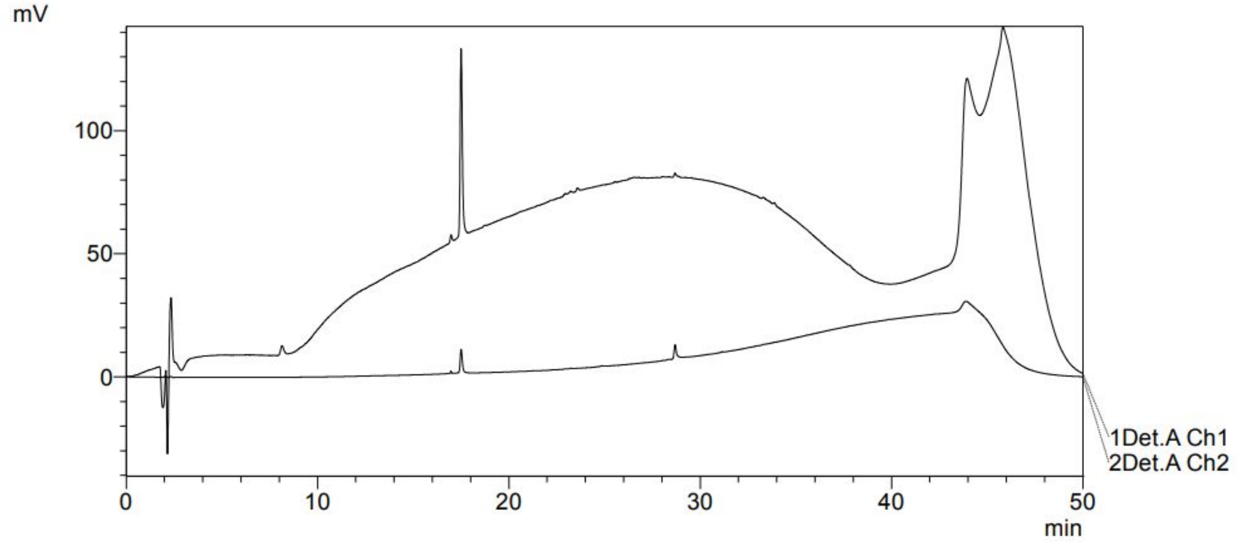

High resolution MS spectrum of CCP4-SCy5 (9).

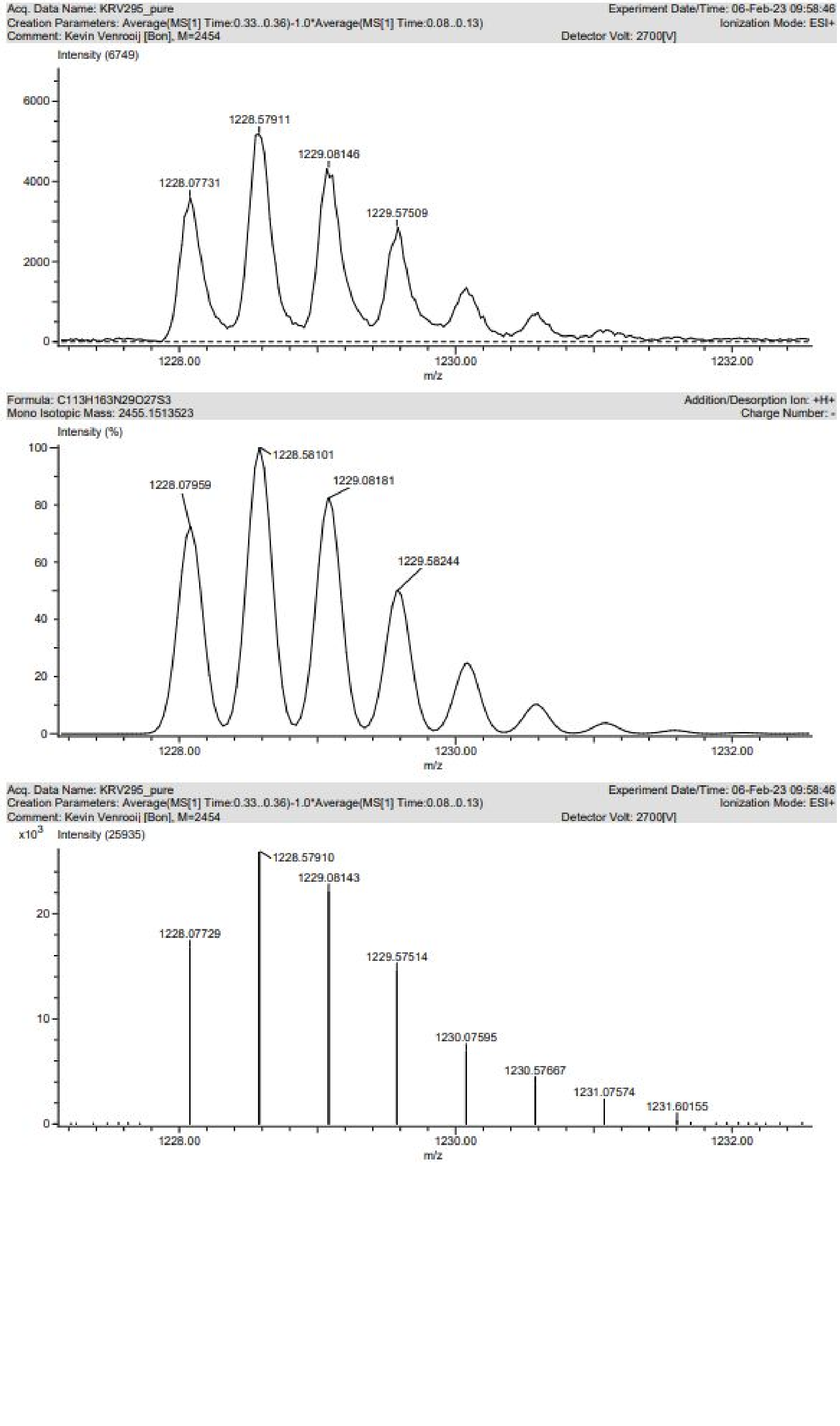

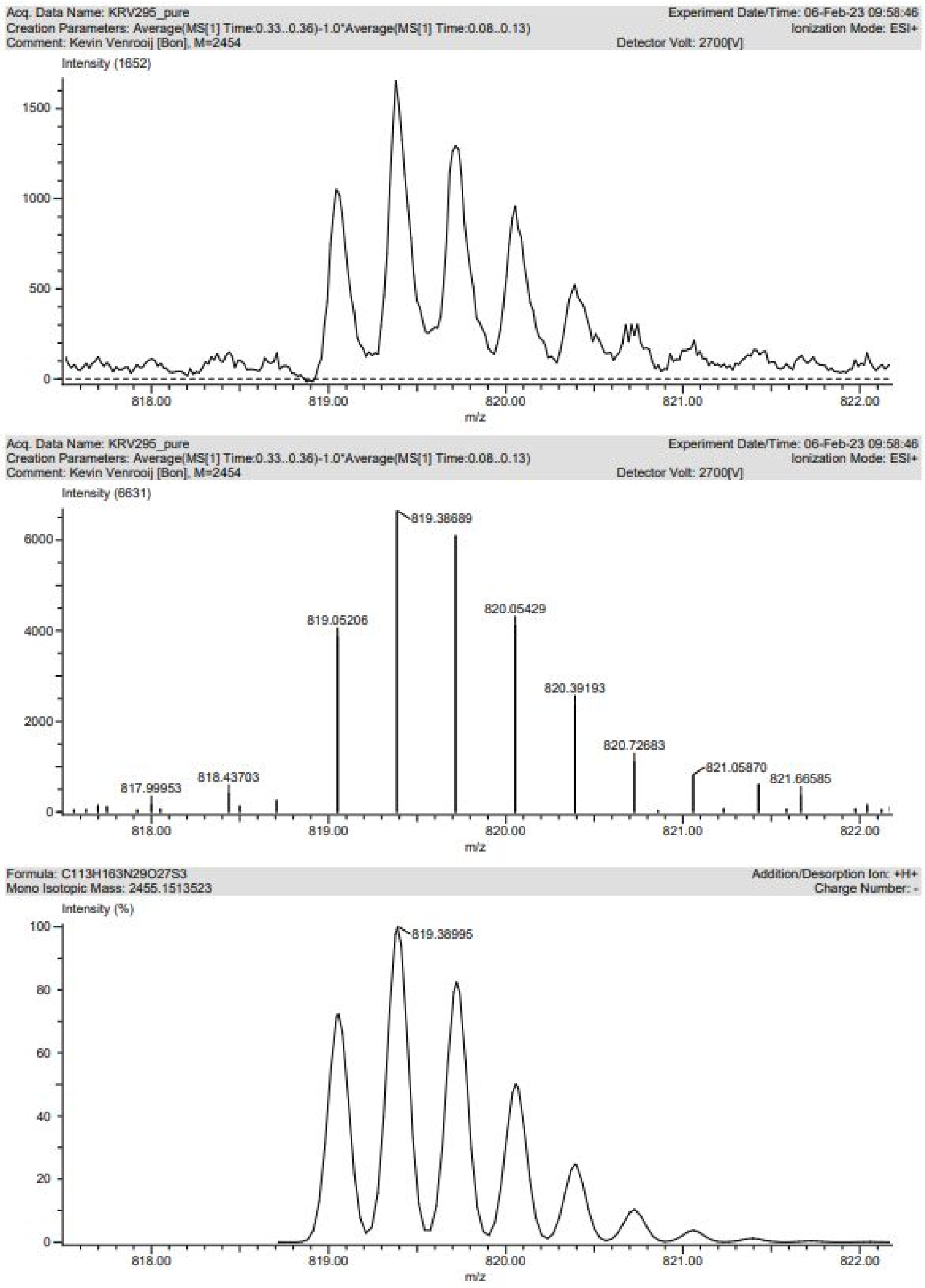
Acq. Data Name: KRV295_pure
Creation Parameters: Average(MS[1] Time:0.33..0.36)-1.0*Average(MS[1] Time:0.08..0.13)
Comment: Kevin Venrooij [Bon], M=2454
Experiment Date/Time: 06-Feb-23 09:58:46
Ionization Mode: ESI+
Detector Volt: 2700[V]
Intensity (1652)
m/z
Acq. Data Name: KRV295_pure
Creation Parameters: Average(MS[1] Time:0.33..0.36)-1.0*Average(MS[1] Time:0.08..0.13)
Comment: Kevin Venrooij [Bon], M=2454
Experiment Date/Time: 06-Feb-23 09:58:46
Ionization Mode: ESI+
Detector Volt: 2700[V]
Intensity (6631)
819.38689
819.05206
820.05429
820.39193
820.72683
821.05870
821.66585
817.99953
818.43703
m/z
Formula: C113H163N29O27S3
Mono Isotopic Mass: 2455.1513523
Addition/Desorption Ion: +H+
Charge Number: -
Intensity (%)
819.38995
m/z

LC-MS analysis of CCP4-SCy5 (9).

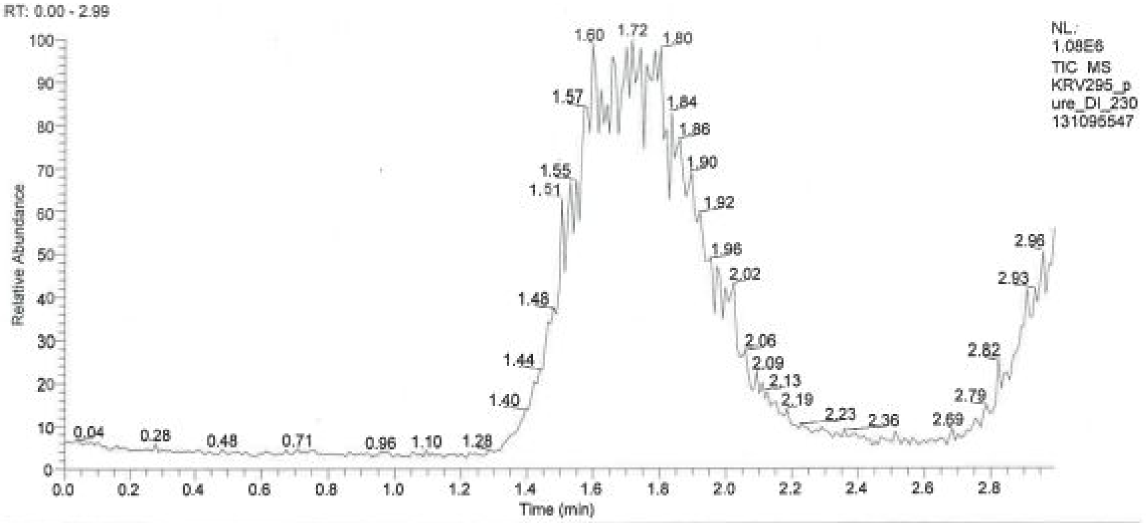
RT: 0.00 - 2.99
NL: 1.08E6 TIC MS KRV295_pure_DI_230131095547
Relative Abundance
Time (min)
1.60
1.72
1.80
1.57
1.84
1.86
1.55
1.51
1.90
1.92
1.96
2.02
1.48
2.06
2.09
1.44
2.13
1.40
2.19
2.23
2.36
2.69
2.79
2.82
2.93
2.96
0.04
0.28
0.48
0.71
0.96
1.10
1.28

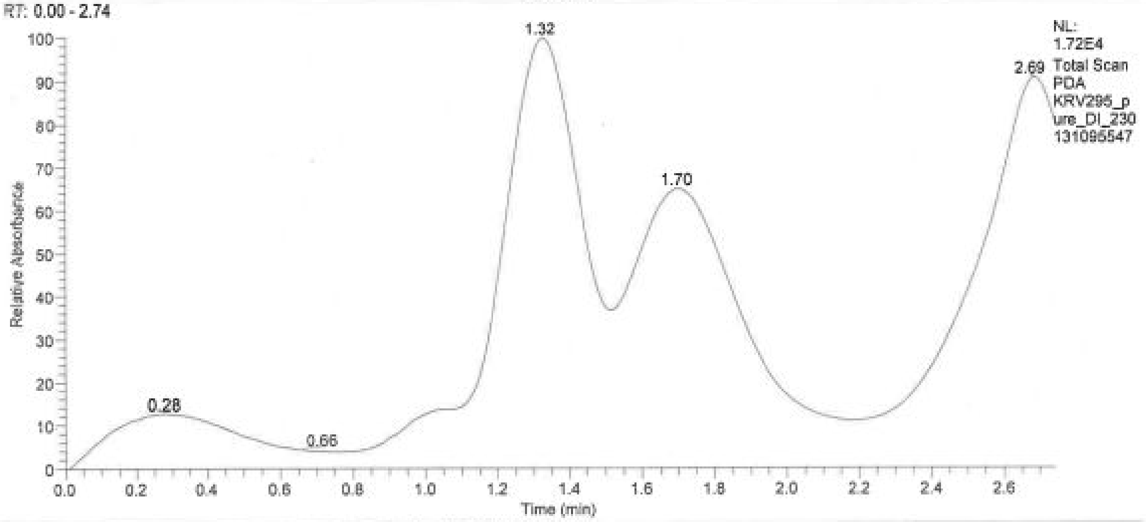
RT: 0.00 - 2.74
NL: 1.72E4 Total Scan PDA KRV295_pure_DI_230131095547
Relative Absorbance
Time (min)
1.32
2.69
1.70
0.28
0.66

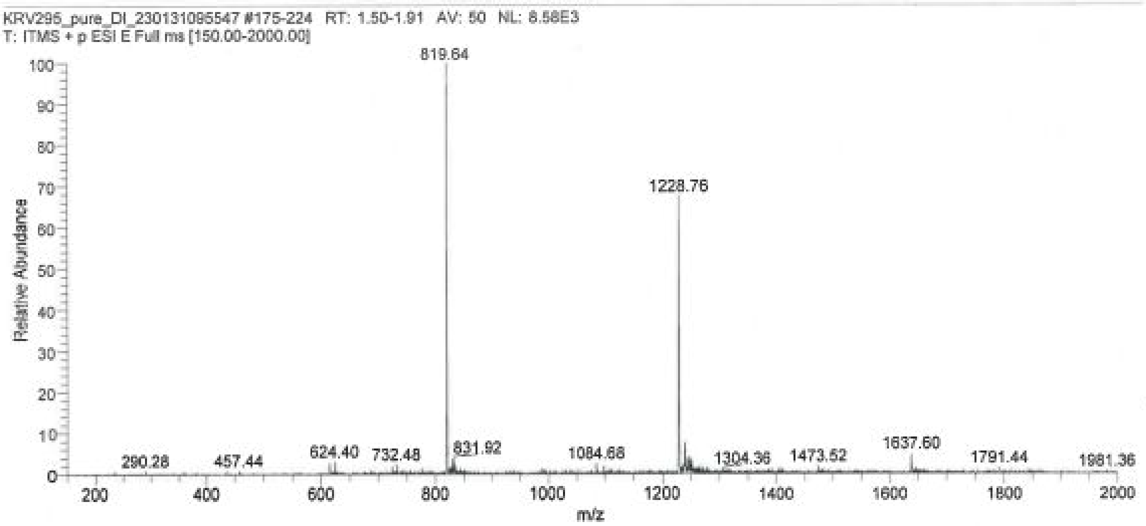
KRV295_pure_DI_230131095547 #175-224 RT: 1.50-1.91 AV: 50 NL: 8.58E3
T: ITMS + p ESI E Full ms [150.00-2000.00]
Relative Abundance
m/z
819.64
1228.76
290.28
457.44
624.40
732.48
831.92
1084.68
1304.36
1473.52
1637.60
1791.44
1981.36

HPLC spectrum of CCP4(dimer)-NH2 (10).

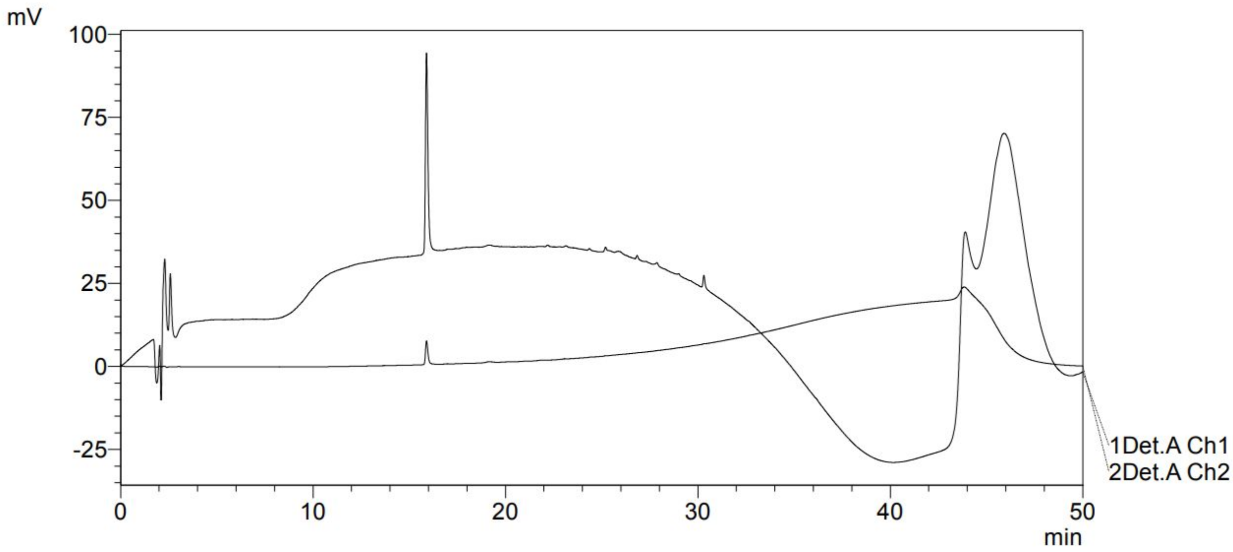

High resolution MS spectrum of CCP4(dimer)-NH2 (10).

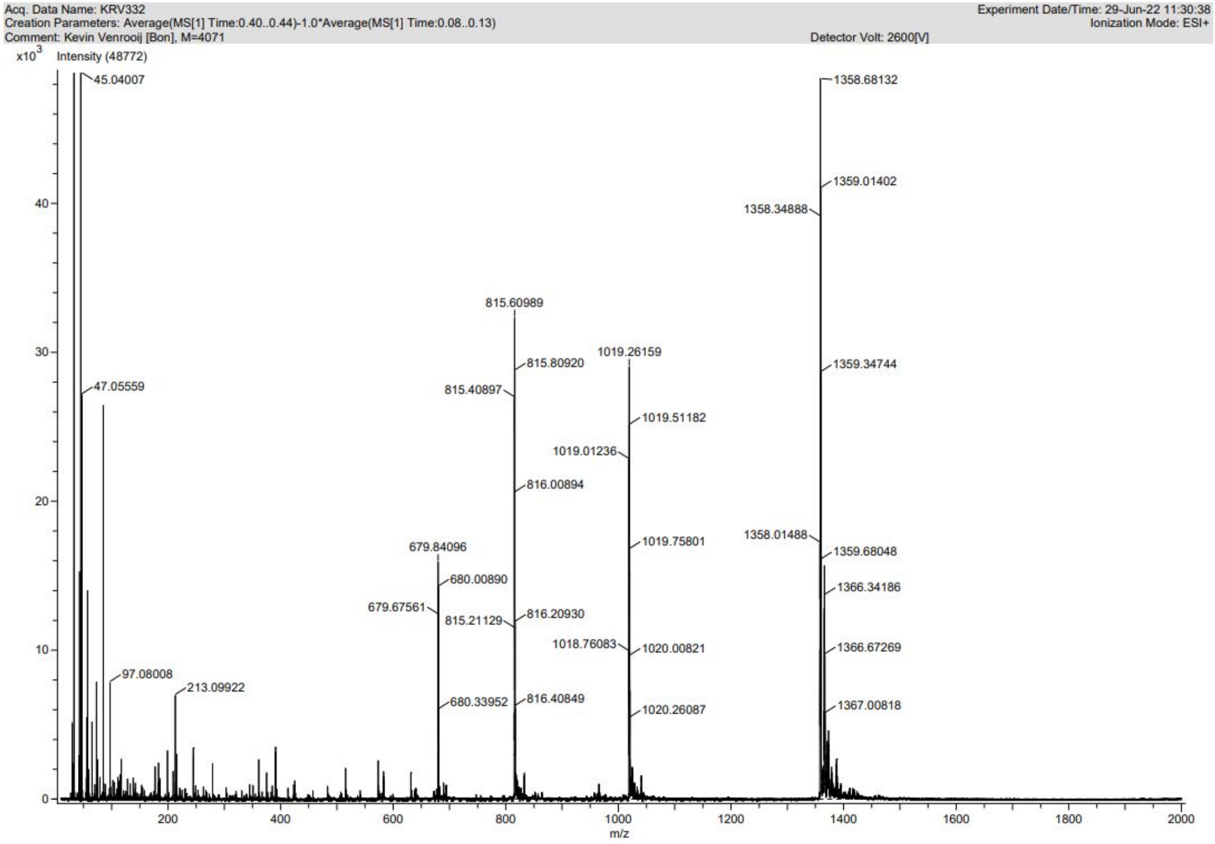

LRMS analysis of CCP4(dimer)-NH2 (10).

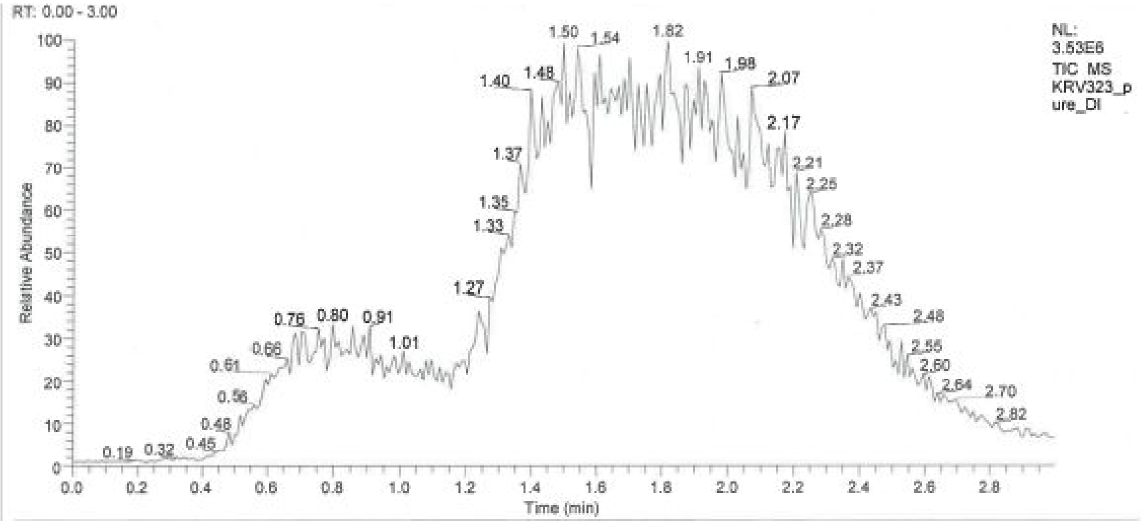
RT: 0.00 - 3.00
NL: 3.53E6 TIC MS KRV323_pure_DI
Relative Abundance
Time (min)
1.50 1.54 1.82 1.91 1.98 2.07 1.40 1.48 2.17 1.37 2.21 2.25 1.35 1.33 2.28 2.32 2.37 1.27 2.43 2.48 0.76 0.80 0.91 1.01 0.66 2.55 2.60 0.61 2.64 2.70 0.56 2.82 0.48 0.19 0.32 0.45
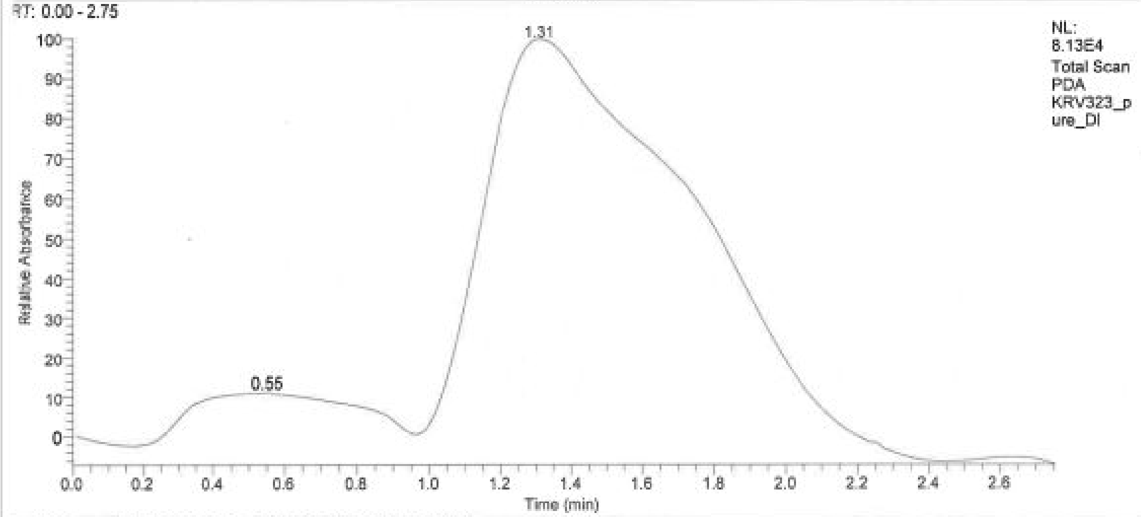
RT: 0.00 - 2.75
NL: 8.13E4 Total Scan PDA KRV323_pure_DI
Relative Absorbance
Time (min)
1.31
0.55
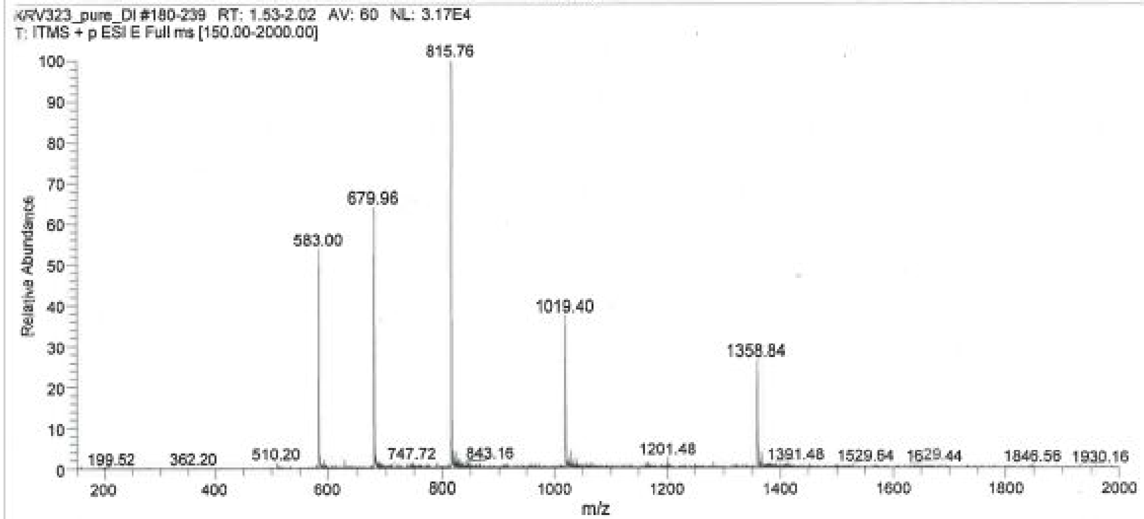
KRV323_pure_DI #180-239 RT: 1.53-2.02 AV: 60 NL: 3.17E4
T: ITMS + p ESI E Full ms [150.00-2000.00]
Relative Abundance
m/z
815.76
679.96
583.00
1019.40
1358.84
199.52 362.20 510.20 747.72 843.16 1201.48 1391.48 1529.64 1629.44 1846.56 1930.16

HPLC spectrum of CCP4(dimer)-Biotin (11).

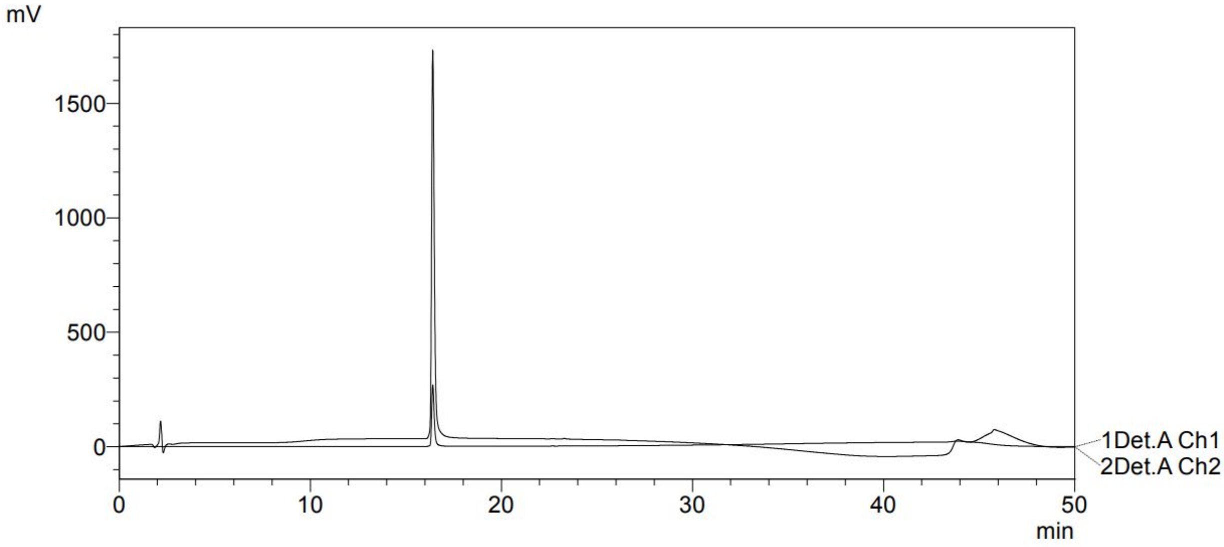

High resolution MS spectrum of CCP4(dimer)-Biotin (11).

Data:KRV268
Sample Name:Kevin Venrooij [Bo], M=4297.1347
Description:
Ionization Mode:ESI+
History:Determine m/z[Peak Detect[Centroid,30,Area];Correct Base[]];Average(MS[1] 0.39..0.43)-1.0*Average(MS[1] 0.10..0.13);Correct Base[5....

Acquired:28-Feb-22 11:05:14
Operator:Accutof
Mass Calibration data:TFANa_ESI+_2000
Created:28-Feb-22 11:12:48
Created by:Accutof

Charge number:3
Tolerance:5.00(ppm), 3.00 .. (mmu)
Unsaturation Number:-1.5 .. 999.0 (Fraction:Both)
Element:^{12}C:187 .. 187, ^{1}H:200 .. 350, ^{14}N:60 .. 80, ^{23}Na:0 .. 0, ^{16}O:30 .. 50, ^{32}S:1 .. 5

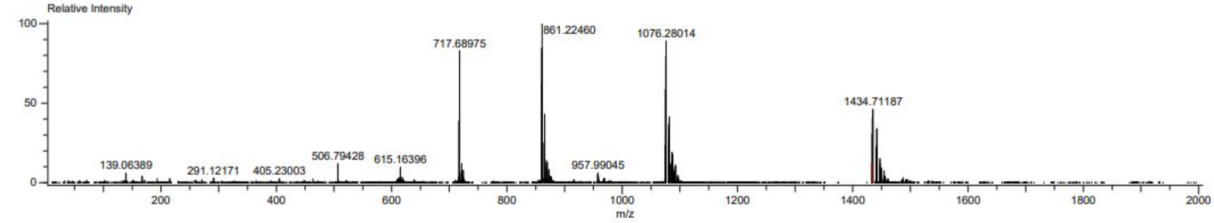

Mass	Decision	Calc. Mass	Mass Difference (mmu)	Mass Difference (ppm)	^{12}C	^{1}H	^{14}N	^{23}Na	^{16}O	^{32}S	Unsaturation Number
4300.15259	+	4300.15816	-5.57	-1.29	187	288	69		44	3	81.5
	?	4300.14739	5.20	1.21	187	274	78		41	1	91.0
	?	4300.15344	-0.85	-0.20	187	276	79		38	2	91.5
	?	4300.15949	-6.90	-1.60	187	278	80		35	3	92.0
	?	4300.15863	-6.04	-1.40	187	280	73		45	1	85.5
	?	4300.14692	5.67	1.32	187	282	74		40	3	87.0
	?	4300.15297	-0.38	-0.09	187	284	75		37	4	87.5
	?	4300.14606	6.53	1.52	187	284	67		50	1	80.5
	?	4300.15211	0.48	0.11	187	286	68		47	2	81.0
	?	4300.15902	-6.43	-1.50	187	286	76		34	5	88.0
	?	4300.14645	6.14	1.43	187	290	70		39	5	83.0
	?	4300.14559	7.00	1.63	187	292	63		49	3	76.5
	?	4300.15164	0.95	0.22	187	294	64		46	4	77.0
	?	4300.15769	-5.10	-1.19	187	296	65		43	5	77.5

LC-MS analysis of CCP4(dimer)-Biotin (11).

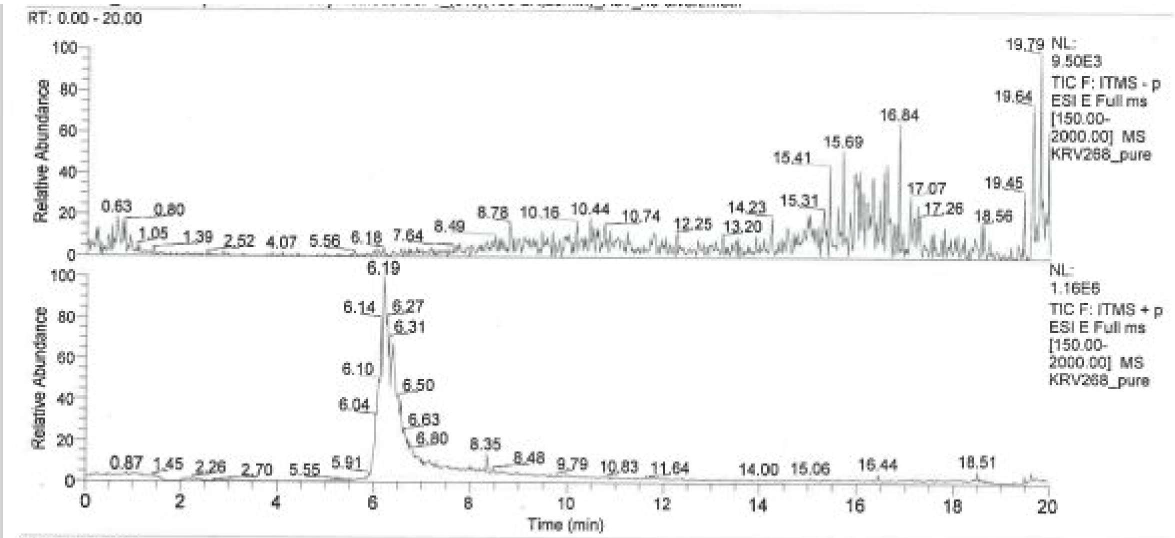
RT: 0.00 - 20.00
Relative Abundance
NL: 9.50E3
TIC F: ITMS - p ESI E Full ms [150.00-2000.00] MS KRV268_pure
19.79
19.64
16.84
15.69
15.41
19.45
17.07
17.26
18.56
15.31
14.23
13.20
12.25
10.74
10.44
10.16
8.78
8.49
7.64
6.18
5.56
4.07
2.52
1.39
1.05
0.80
0.63
NL: 1.16E6
TIC F: ITMS + p ESI E Full ms [150.00-2000.00] MS KRV268_pure
6.19
6.14
6.27
6.31
6.10
6.50
6.04
6.63
6.80
8.35
8.48
9.79
10.83
11.64
14.00
15.06
16.44
18.51
5.91
5.55
2.70
2.26
1.45
0.87
Time (min)

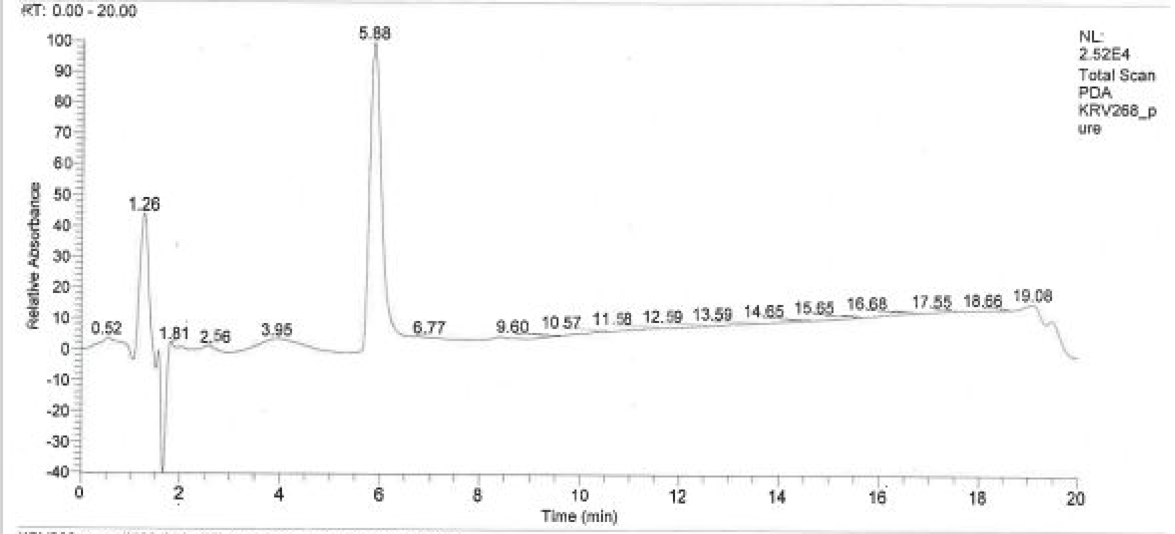
RT: 0.00 - 20.00
NL: 2.52E4
Total Scan PDA KRV268_pure
Relative Absorbance
5.88
1.26
0.52
1.81
2.56
3.95
6.77
9.60
10.57
11.58
12.59
13.59
14.65
15.65
16.68
17.55
18.66
19.08
Time (min)

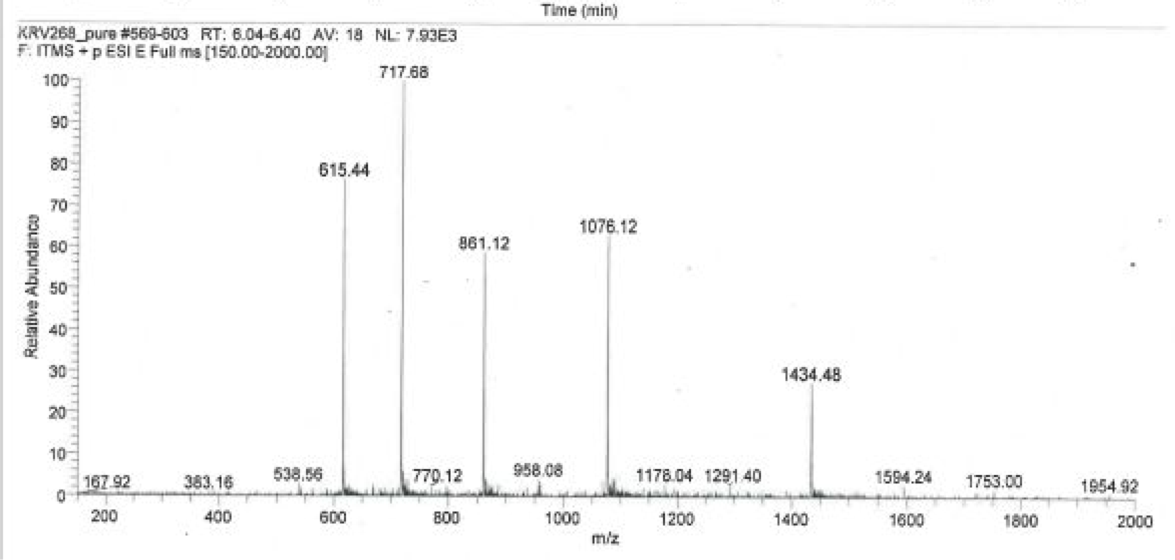
KRV268_pure #569-603 RT: 6.04-6.40 AV: 18 NL: 7.93E3
F: ITMS + p ESI E Full ms [150.00-2000.00]
Relative Abundance
717.68
615.44
861.12
1076.12
1434.48
167.92
383.16
538.56
770.12
958.08
1178.04
1291.40
1594.24
1753.00
1954.92
m/z

HPLC spectrum of CCP4(dimer)-AF594 (12).

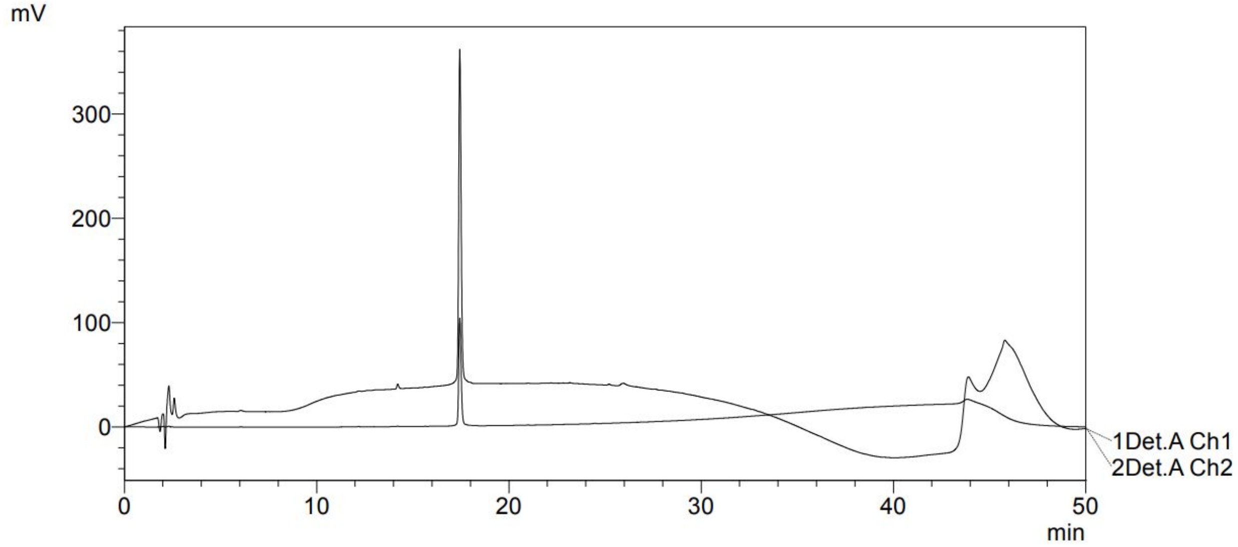

High resolution MS spectrum of CCP4(dimer)-AF594 (12).

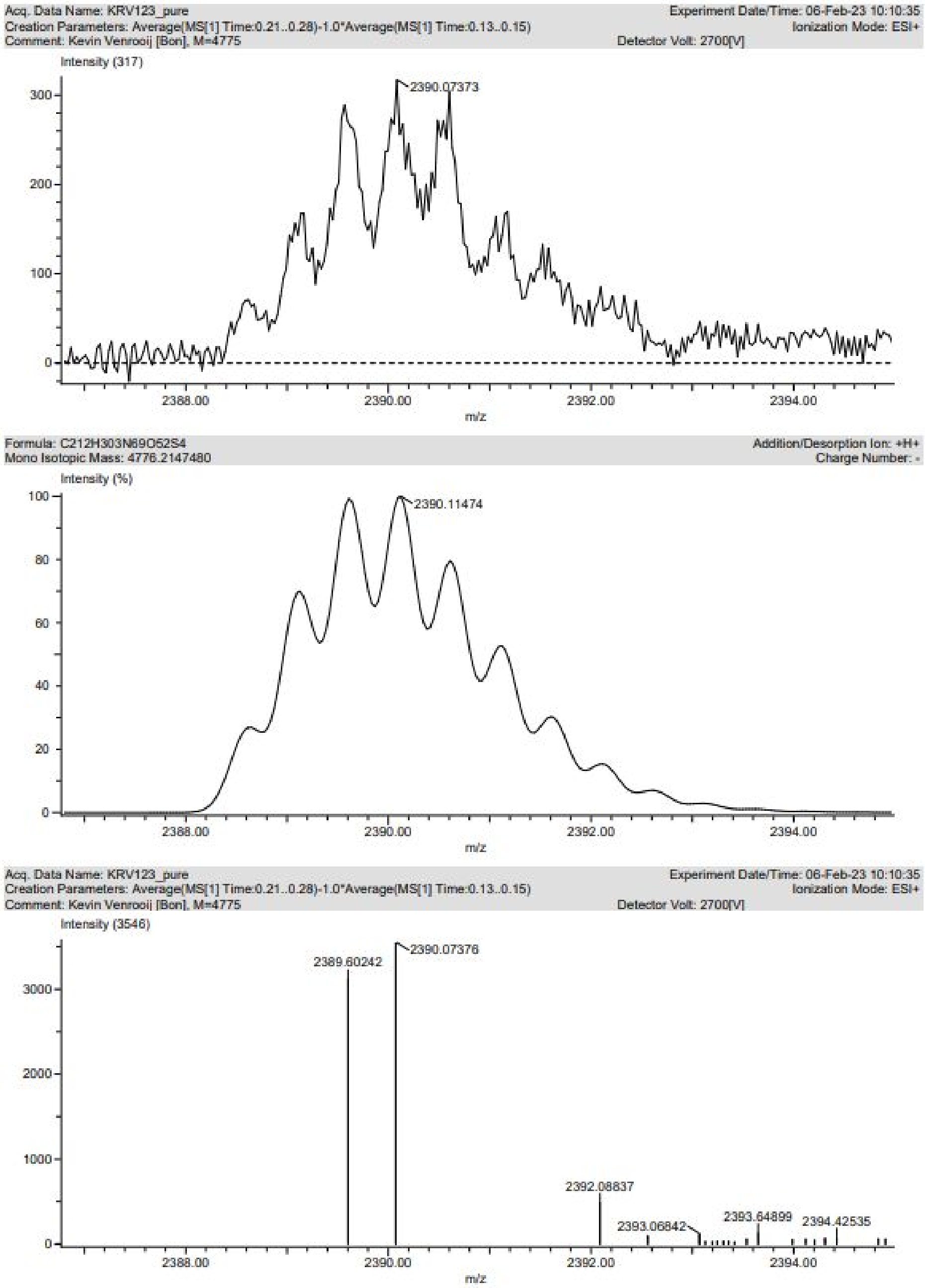

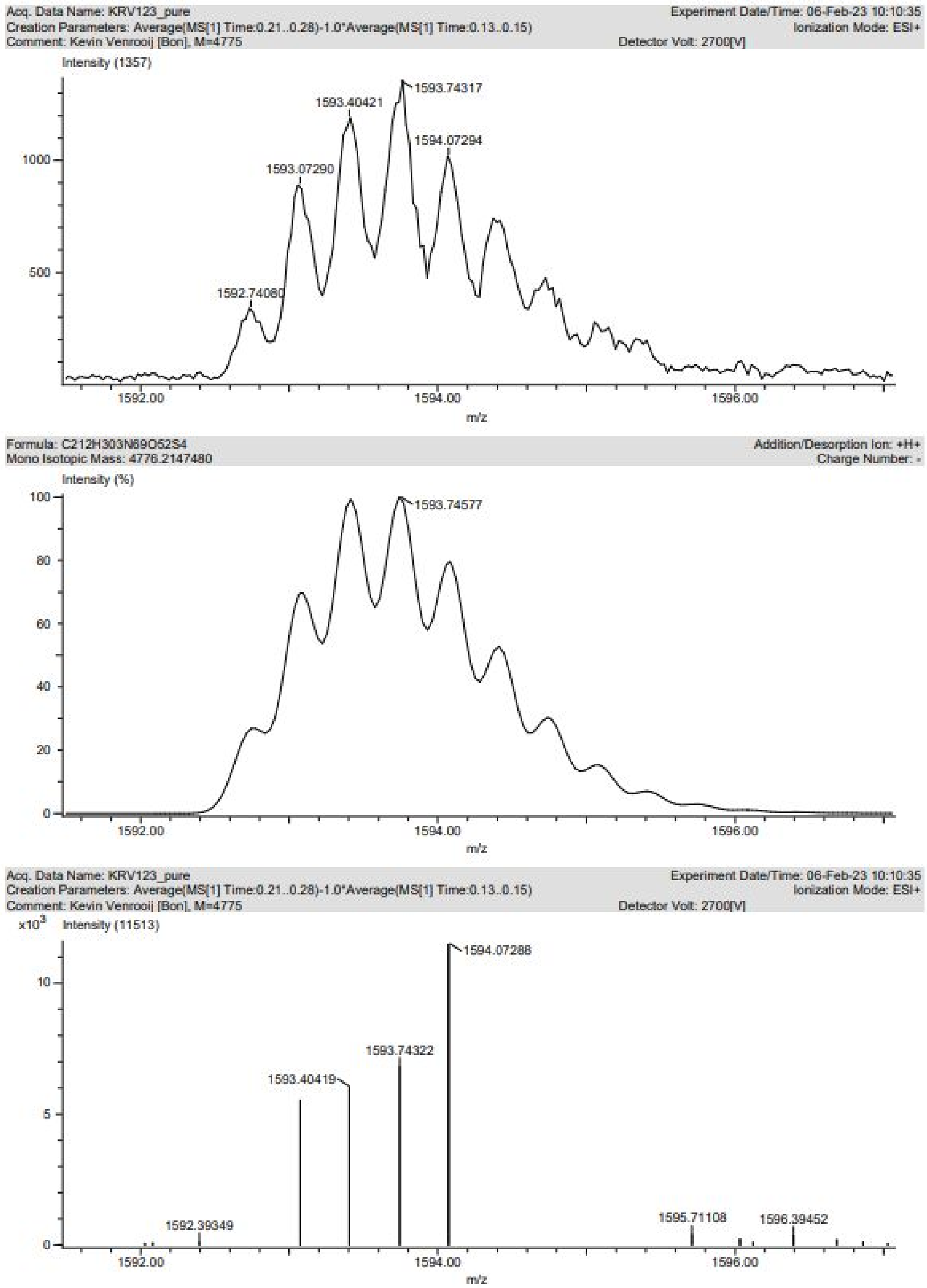
Acq. Data Name: KRV123_pure
Experiment Date/Time: 06-Feb-23 10:10:35
Creation Parameters: Average(MS[1] Time:0.21..0.28)-1.0*Average(MS[1] Time:0.13..0.15)
Ionization Mode: ESI+
Comment: Kevin Venrooij [Bon], M=4775
Detector Volt: 2700[V]
Intensity (1357)
1593.74317
1593.40421
1594.07294
1593.07290
1592.74060
m/z
Formula: C212H303N69O52S4
Addition/Desorption Ion: +H+
Mono Isotopic Mass: 4776.2147480
Charge Number: -
Intensity (%)
1593.74577
m/z
Acq. Data Name: KRV123_pure
Experiment Date/Time: 06-Feb-23 10:10:35
Creation Parameters: Average(MS[1] Time:0.21..0.28)-1.0*Average(MS[1] Time:0.13..0.15)
Ionization Mode: ESI+
Comment: Kevin Venrooij [Bon], M=4775
Detector Volt: 2700[V]
x10^3 Intensity (11513)
1594.07288
1593.74322
1593.40419
1592.39349
1595.71108
1596.39452
m/z

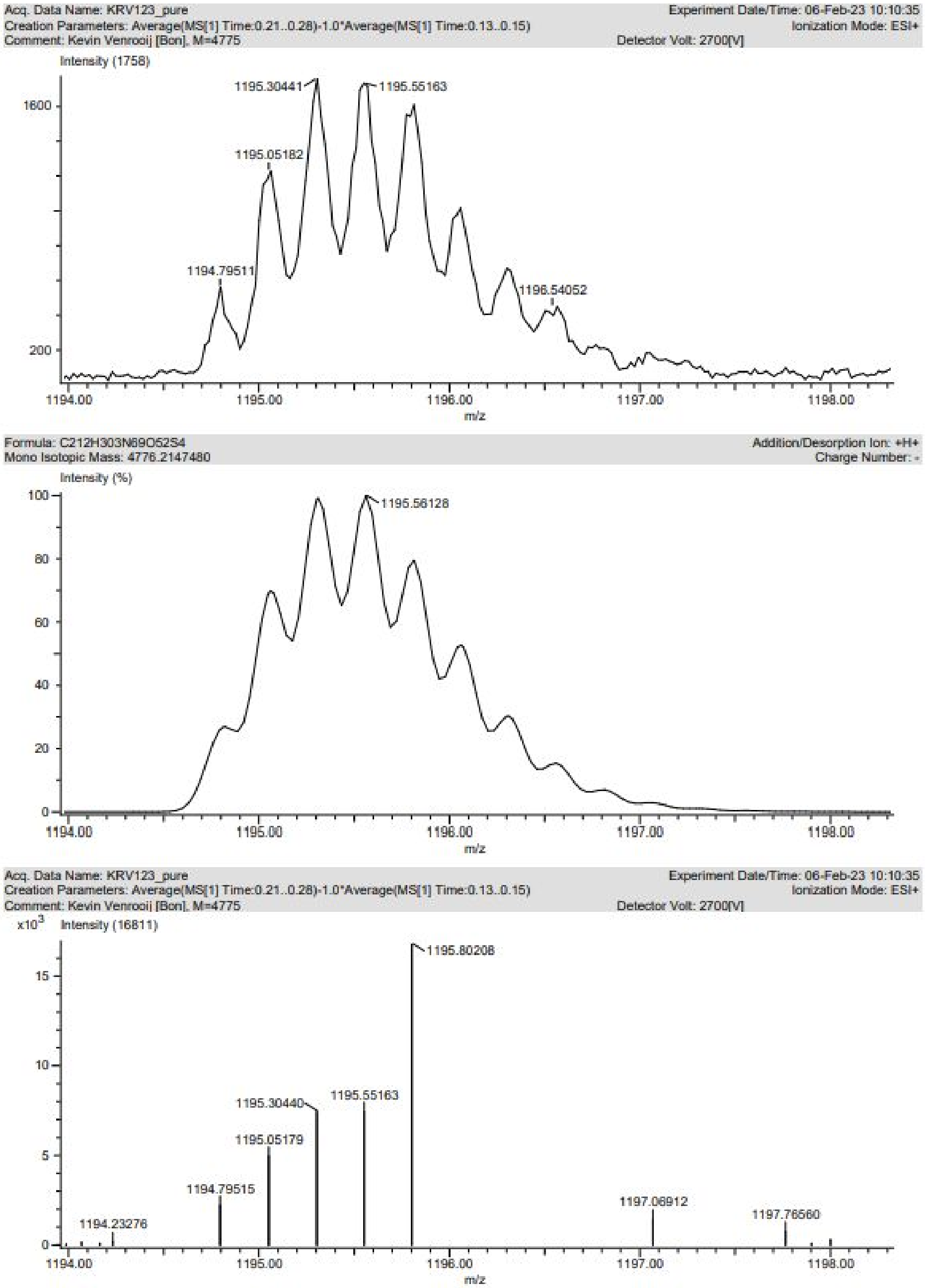
Acq. Data Name: KRV123_pure
Experiment Date/Time: 06-Feb-23 10:10:35
Creation Parameters: Average(MS[1] Time:0.21..0.28)-1.0*Average(MS[1] Time:0.13..0.15)
Ionization Mode: ESI+
Comment: Kevin Venrooij [Bon], M=4775
Detector Volt: 2700[V]
Intensity (1758)
1195.30441
1195.55163
1195.05182
1194.79511
1196.54052
m/z
Formula: C212H303N69O52S4
Addition/Desorption Ion: +H+
Mono Isotopic Mass: 4776.2147480
Charge Number: -
Intensity (%)
1195.56128
m/z
Acq. Data Name: KRV123_pure
Experiment Date/Time: 06-Feb-23 10:10:35
Creation Parameters: Average(MS[1] Time:0.21..0.28)-1.0*Average(MS[1] Time:0.13..0.15)
Ionization Mode: ESI+
Comment: Kevin Venrooij [Bon], M=4775
Detector Volt: 2700[V]
x10^3 Intensity (16811)
1195.80208
1195.30440
1195.55163
1195.05179
1194.79515
1194.23276
1197.06912
1197.76560
m/z

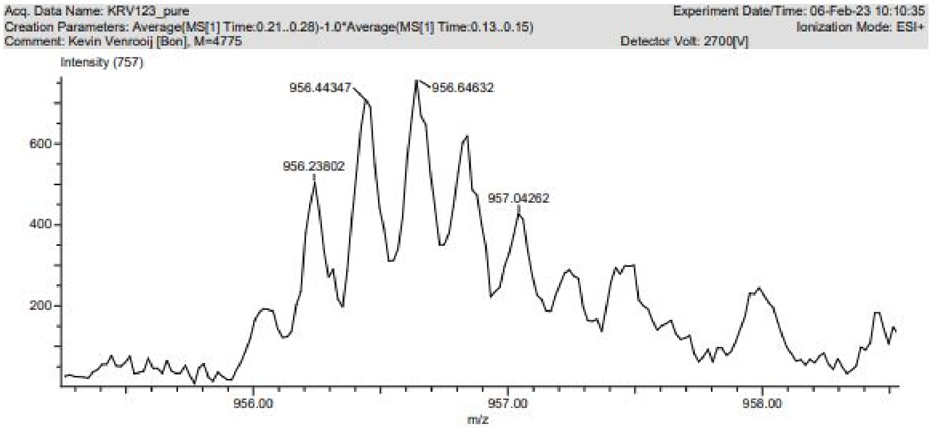
Acq. Data Name: KRV123_pure
Experiment Date/Time: 06-Feb-23 10:10:35
Creation Parameters: Average(MS[1] Time:0.21..0.28)-1.0*Average(MS[1] Time:0.13..0.15)
Ionization Mode: ESI+
Comment: Kevin Venrooij [Bon], M=4775
Detector Volt: 2700[V]
Intensity (757)
956.44347
956.64632
956.23802
957.04262
956.00
957.00
958.00
m/z

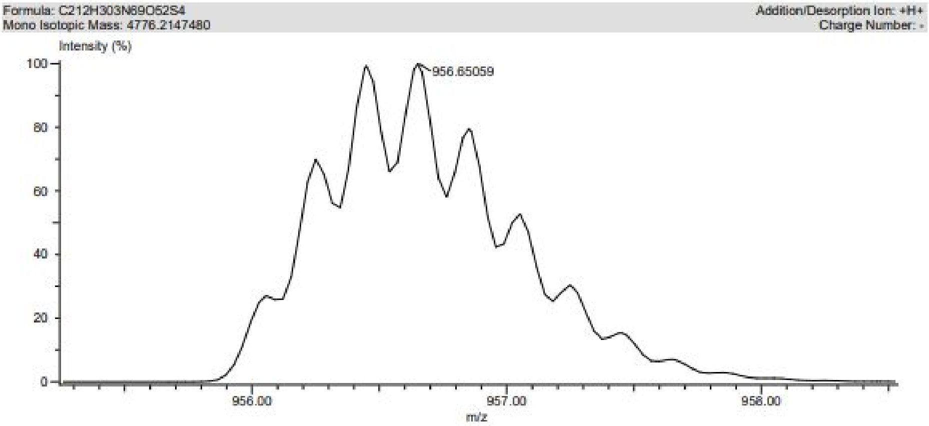
Formula: C212H303N69O52S4
Addition/Desorption Ion: +H+
Mono Isotopic Mass: 4776.2147480
Charge Number: -
Intensity (%)
956.65059
956.00
957.00
958.00
m/z

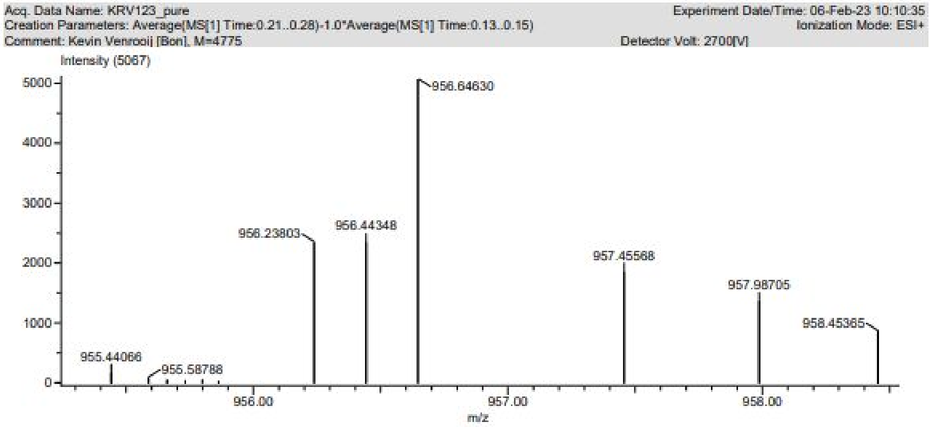
Acq. Data Name: KRV123_pure
Experiment Date/Time: 06-Feb-23 10:10:35
Creation Parameters: Average(MS[1] Time:0.21..0.28)-1.0*Average(MS[1] Time:0.13..0.15)
Ionization Mode: ESI+
Comment: Kevin Venrooij [Bon], M=4775
Detector Volt: 2700[V]
Intensity (5067)
956.64630
956.23803
956.44348
957.45568
957.98705
958.45365
955.44066
955.58788
956.00
957.00
958.00
m/z

LRMS analysis of CCP4(dimer)-AF594 (12).

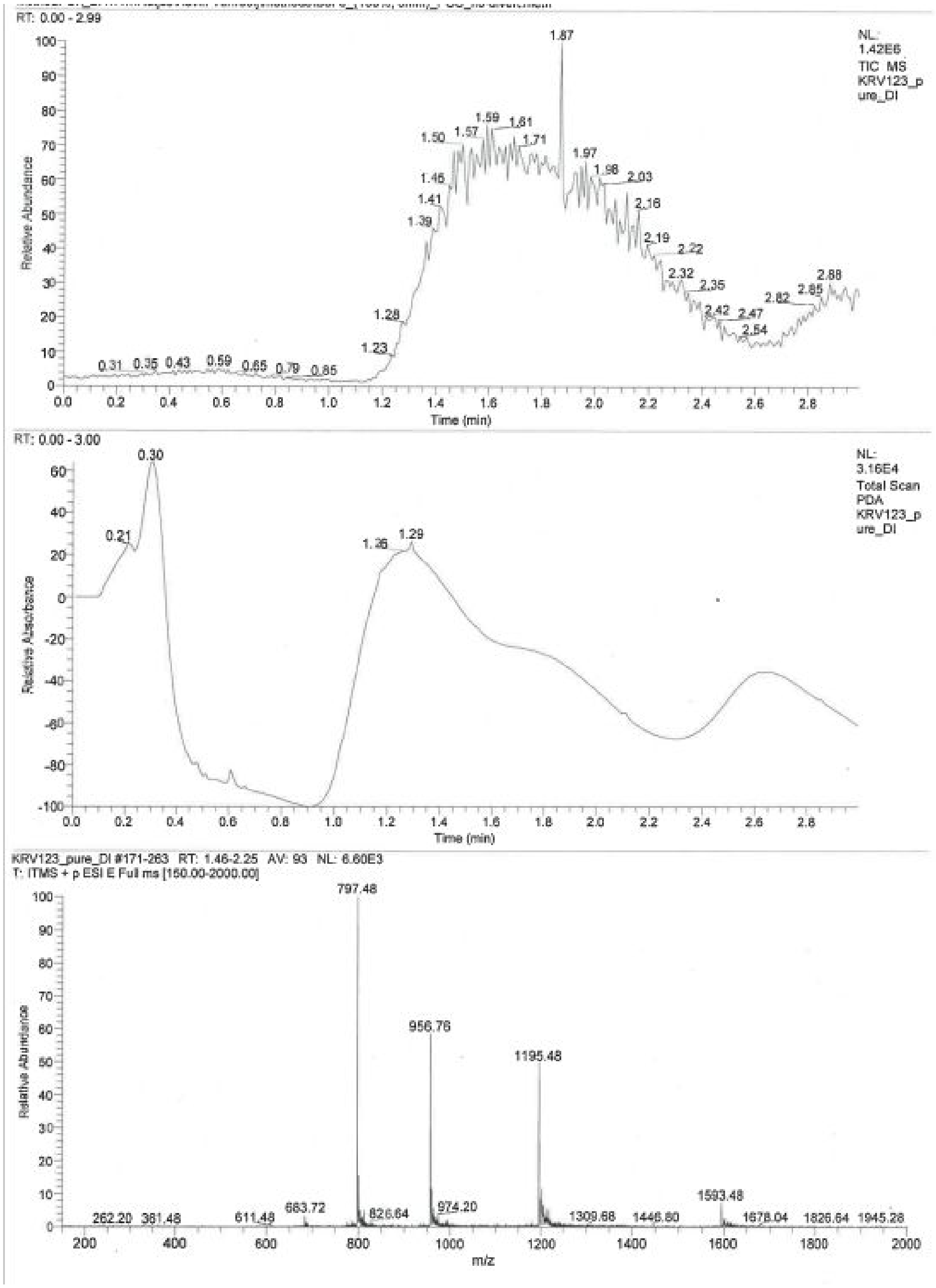

HPLC spectrum of CCP4(dimer)-SCy5 (13).

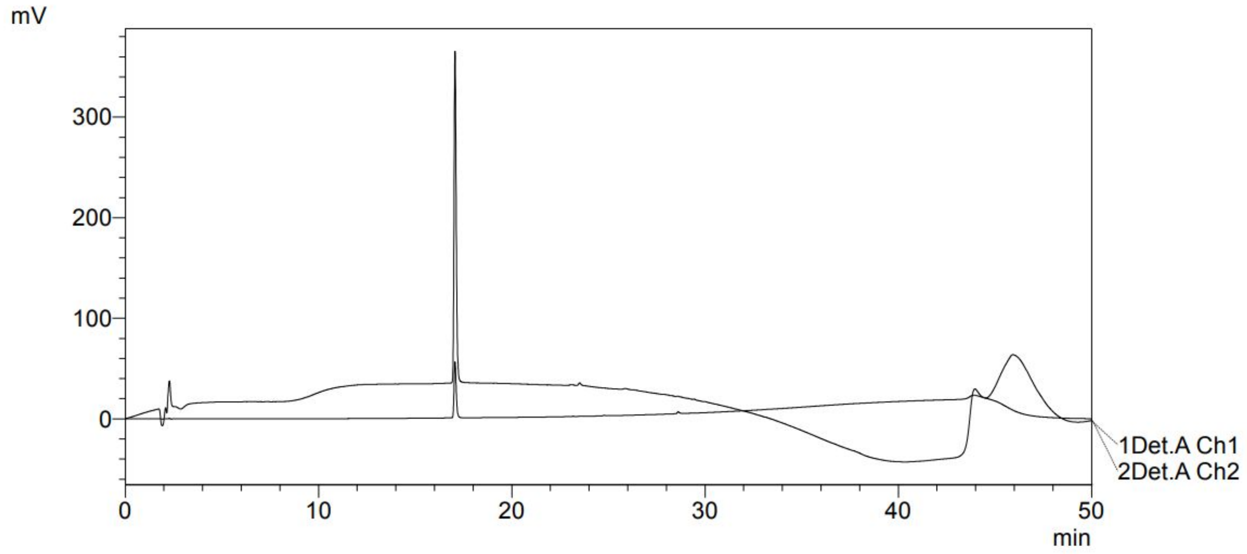

High resolution MS spectrum of CCP4(dimer)-SCy5 (13).

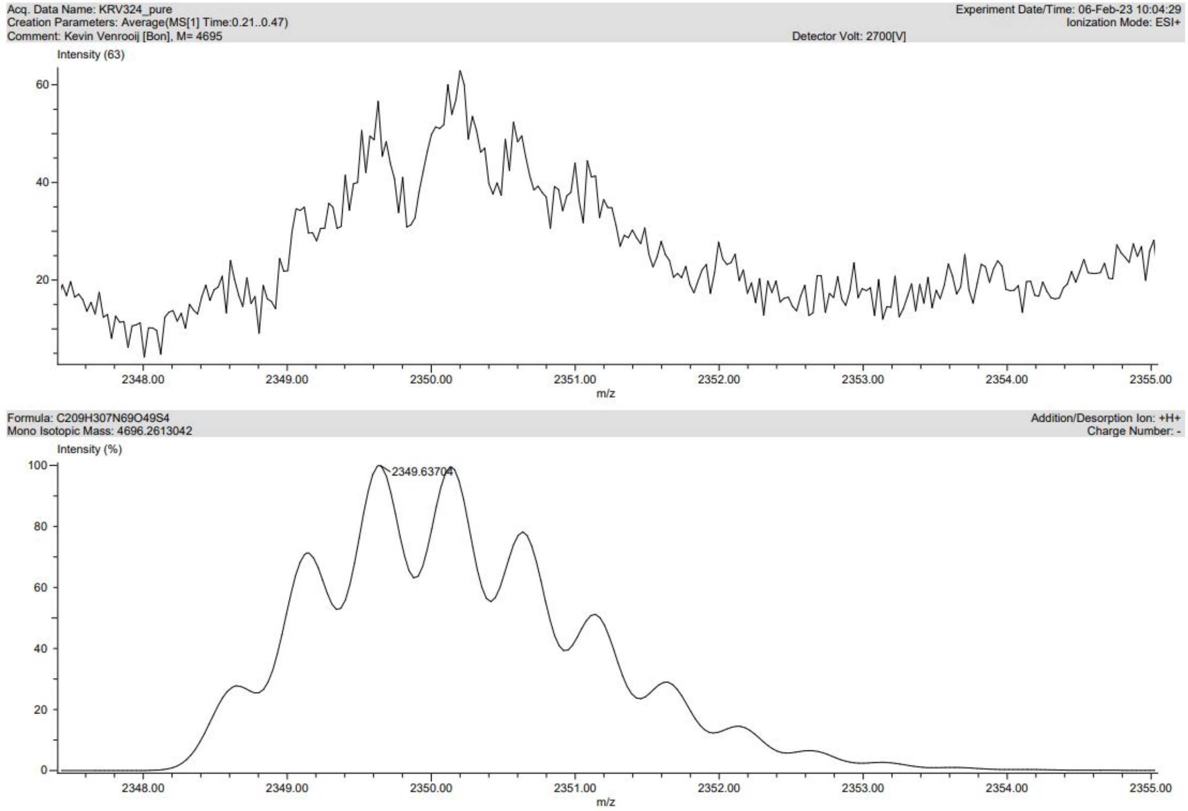

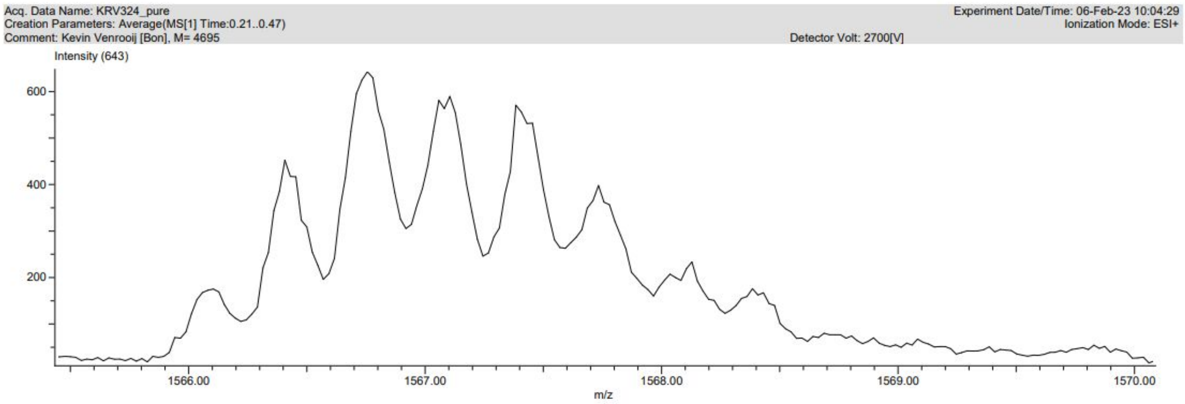

Acq. Data Name: KRV324_pure
Creation Parameters: Average(MS[1] Time:0.21..0.47)
Comment: Kevin Venrooij [Bon], M= 4695
Experiment Date/Time: 06-Feb-23 10:04:29
Ionization Mode: ESI+
Detector Volt: 2700[V]
Intensity (643)
600
400
200
1566.00
1567.00
1568.00
1569.00
1570.00
m/z

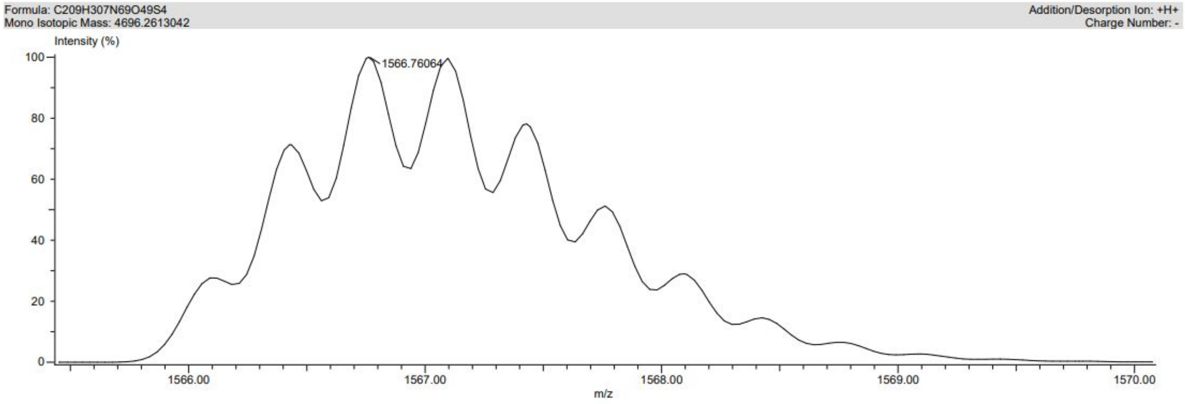

Formula: C209H307N69O49S4
Mono Isotopic Mass: 4696.2613042
Addition/Desorption Ion: +H+
Charge Number: -
Intensity (%)
100
80
60
40
20
0
1566.76064
1566.00
1567.00
1568.00
1569.00
1570.00
m/z

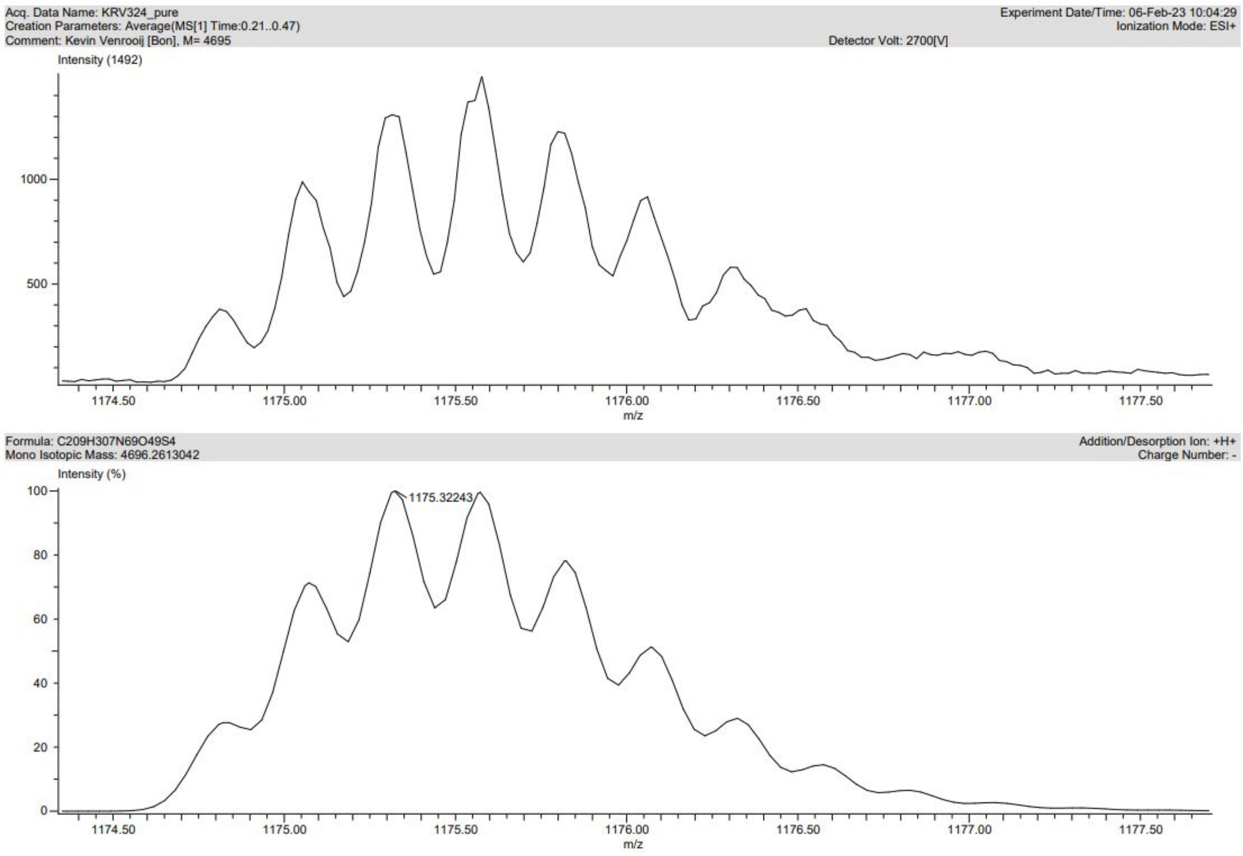
Acq. Data Name: KRV324_pure
Creation Parameters: Average(MS[1] Time:0.21..0.47)
Comment: Kevin Venrooij [Bon], M= 4695
Experiment Date/Time: 06-Feb-23 10:04:29
Ionization Mode: ESI+
Detector Volt: 2700[V]
Intensity (1492)
1000
500
1174.50
1175.00
1175.50
1176.00
1176.50
1177.00
1177.50
m/z
Formula: C209H307N69O49S4
Mono Isotopic Mass: 4696.2613042
Addition/Desorption Ion: +H+
Charge Number: -
Intensity (%)
100
80
60
40
20
0
1175.32243
1174.50
1175.00
1175.50
1176.00
1176.50
1177.00
1177.50
m/z

Acq. Data Name: KRV324_pure
Creation Parameters: Average(MS[1] Time:0.21..0.47)
Comment: Kevin Venrooij [Bon], M= 4695

Experiment Date/Time: 06-Feb-23 10:04:29
Ionization Mode: ESI+
Detector Volt: 2700[V]

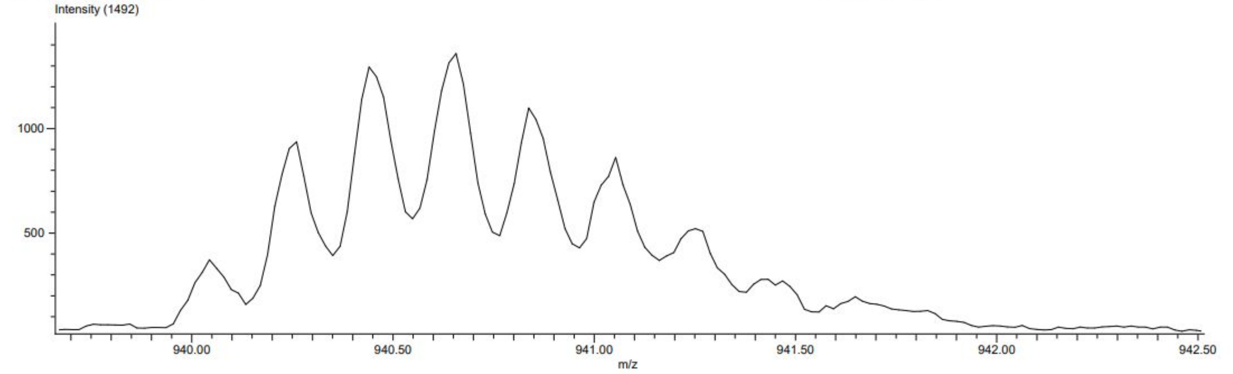

Formula: C209H307N69O49S4
Mono Isotopic Mass: 4696.2613042

Addition/Desorption Ion: +H+
Charge Number: -

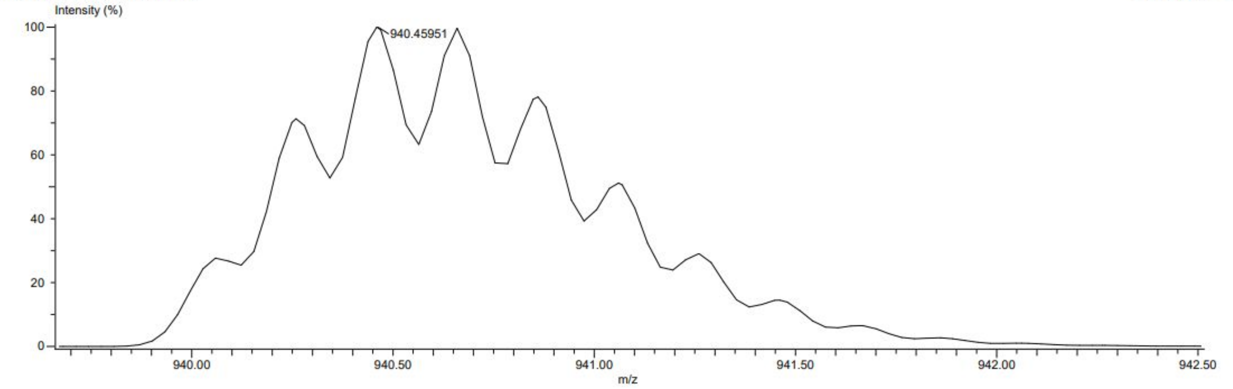

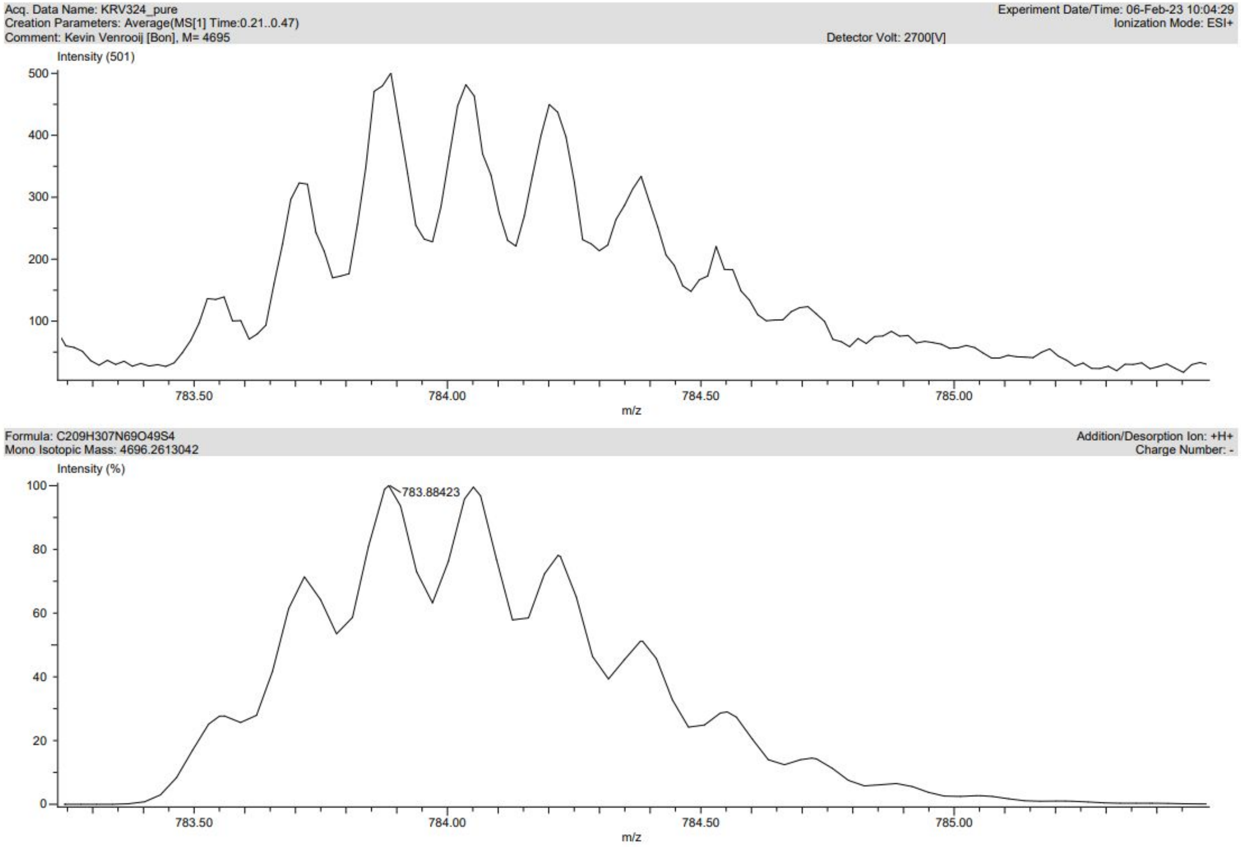

Acq. Data Name: KRV324_pure
Creation Parameters: Average(MS[1] Time:0.21..0.47)
Comment: Kevin Venrooij [Bon], M= 4695
Experiment Date/Time: 06-Feb-23 10:04:29
Ionization Mode: ESI+
Detector Volt: 2700[V]
Intensity (501)
500
400
300
200
100
783.50
784.00
784.50
785.00
m/z
Formula: C209H307N69O49S4
Mono Isotopic Mass: 4696.2613042
Addition/Desorption Ion: +H+
Charge Number: -
Intensity (%)
100
80
60
40
20
0
783.88423
783.50
784.00
784.50
785.00
m/z

LRMS analysis of CCP4(dimer)-SCy5 (13).

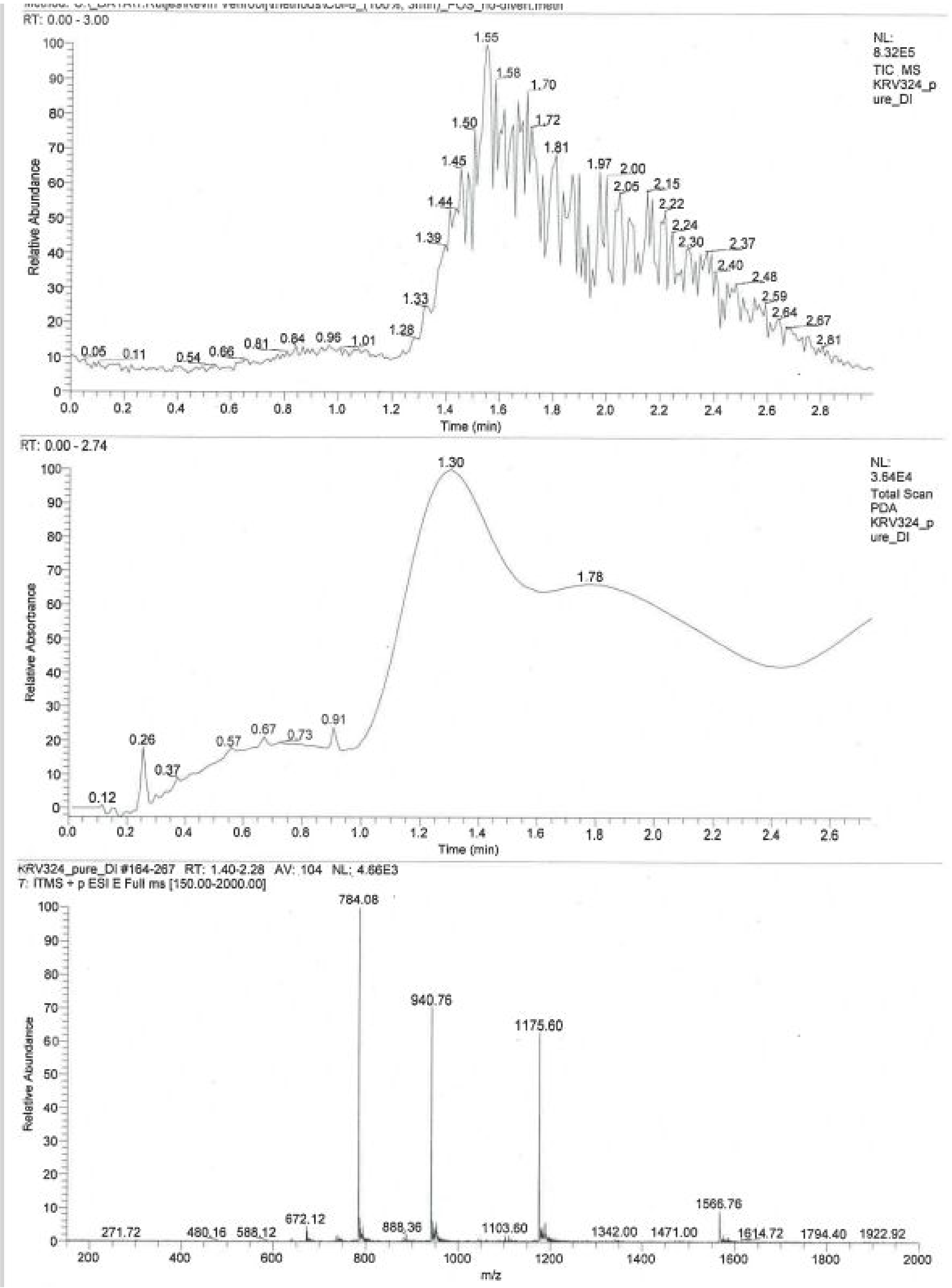

Supplement: Supplementary file 1 — mp3c00527_si_001.pdf [file mp3c00527_si_001.pdf]
